# Respectful delivery care and associated factors among mothers delivered in public health facilities of Dessie city, Northeast Ethiopia: a cross-sectional study

**DOI:** 10.1186/s12905-022-01713-1

**Published:** 2022-04-21

**Authors:** Melaku Yalew, Dabere Nigatu, Toyeb Yasin, Bereket Kefale, Yitayish Damtie

**Affiliations:** 1 Department of Reproductive and Family Health, School of Public Health, College of Medicine and Health Sciences, Wollo University, Dessie, Ethiopia; 2 Department of Epidemiology and Biostatistics, School of Public Health, College of Medicine and Health Sciences, Wollo University, Dessie, Ethiopia; 3 Department of Reproductive Health and Population Studies, School of Public Health, College of Medicine and Health Sciences, Bahir Dar University, Bahir Dar, Ethiopia; 4 Department of Health Service Management, School of Public Health, College of Medicine and Health Sciences, Wollo University, Dessie, Ethiopia

**Keywords:** Respect, Delivery care, Right of women, Dessie, Ethiopia

## Abstract

**Background:**

The government of Ethiopia has been implementing compassionate, respectful, and caring strategies to increase institutional delivery and decrease maternal mortality in recent years. There is limited evidence on respectful delivery care and associated factors in low-income countries like Ethiopia. Therefore, this study aimed to assess the proportion of respectful delivery care and associated factors among mothers delivered in the health facilities of Dessie city, Northeast Ethiopia.

**Methods:**

A health facility-based cross-sectional study was conducted among a total of 390 mothers from April 16 to May 30, 2018. A pretested structured interviewer-administered questionnaire was used to collect the data. The data were entered into Epidata and analyzed using Stata/SE 14. Binary logistic regression analysis was used to identify associated factors. Variables having P-value less than 0.2 in the bivariable regression were selected as a candidate for multi-variable regression. Adjusted odds ratio (AOR) with 95% confidence interval (CI) was estimated to measure the strength and direction of the association respectively.

**Results:**

The proportion of respectful delivery care among mothers delivered in public health facilities of Dessie city was 43.4%, 95% CI (39.1%, 47.6%). It was found to be 34.9% in hospital and 74.1 in health center. Respectful delivery care was associated with day time delivery [AOR = 2.23, 95% CI (1.30, 3.82)], any maternal and/or fetal complications [AOR = 0.50, 95% CI (0.27, 0.94)], gave birth in health center [AOR = 3.22, 95% CI (1.61, 6.46)] and educated mothers [AOR = 2.87, 95% CI (1.18, 7.01)].

**Conclusions:**

The proportion of respectful delivery care in the study area was low as compared to the government emphasis and other works of literature. This study indicated that any maternal and/or newborn complications, daytime delivery, giving birth in a health center, and maternal education were associated with respectful delivery care. Women empowerment through education could be a recalled intervention for respectful care.

**Supplementary Information:**

The online version contains supplementary material available at 10.1186/s12905-022-01713-1.

## Background

Respectful maternal care is an approach that focuses on the individual, based on principles of ethics and respect for human rights [1]. Since respect and dignity have been identified as essential components of good quality of care [2, 3], the World Health Organization (WHO) has launched a statement on prevention and elimination of disrespect and abuse during childbirth [4, 5]. Even every woman has the right to get respectful care, many women in the world have experienced the situation of abuse during childbirth [6–8]. A study conducted in India indicated that only 45.3% of women were treated respectfully [9]. It becomes worse in Nigeria as only 2% were respected and 86.1% in Malawi were treated respectfully [10, 11] while it was ranged between 22 and 78.9% in Ethiopia [12, 13].

Respectful delivery care is one of the opportunities for increasing maternal health service utilization [9, 14–19]. Sixty-one percent of mistreated women wanted to deliver by someone else and only 27% intend to deliver in a facility next time [20, 21]. Seventy-nine percent of maternal deaths were resulted due to substandard care [22]. Poor quality of delivery care constitutes a 10% case fatality rate [14] and 2.9 million neonatal deaths per year could be prevented through timely and skilled delivery care [23, 24]. Any form of disrespect from maternal health care providers (MHCPs) resulted in distress and fear among mothers and led to an absence of trust in providers [25].

Numerous studies indicated that maternal respect during childbirth could be influenced by maternal characteristics (age, educational status, occupation, residence) [1, 26, 27], health care provider’s characteristics (type of profession, estimated work hour per day, sex of profession) [25, 28–32] and health facilities characteristics (type of facility) [1, 13, 14, 25]. The government of Ethiopia also adopts SDG and Growth and Transformation Plan (GTP II). These strategies emphasize maternal health to decrease MMR (below 199 and 267/100,000 live birth respectively) and increase institutional delivery [33, 34]. In spite of the fact that 412/100,000 women were died due to pregnancy-related causes, only 62% of pregnant women had ANC visits at least one time and only 26% of them gave birth in health facilities [35]. Although there are some studies on respectful delivery care, those studies are limited and not large enough for policy development. There was no similar study conducted in the study setting as problems are non-stationary and Ethiopia is a multi-cultural country and local or context-specific evidence is needed for planning. In addition, the studies are more qualitative and less emphasized on the factors, especially health facility and health care provider factors (type of health facility, sex of health care provider, the profession of health care provider, provider experience, estimated work hour per day). As a result, this study aimed to assess the proportion of respectful delivery care and associated factors among mothers who delivered in the health facilities of Dessie city, Northeast Ethiopia.

## Methods

### Study setting, design, and population

A facility-based cross-sectional study design was conducted in the health facilities of Dessie city, Northeast Ethiopia. Dessie is the capital city of South Wollo Zone including its town administration, which is located 401 km to the North of Addis Ababa. The study was conducted from April 16 to May 30, 2018. Women who gave birth in the health facilities of Dessie city during the data collection time were included in the study and mothers who took general anesthesia during cesarean section delivery were excluded since they didn’t know in what way the provider treated them until full recovery that may lead to bias.

### Sample size determination and sampling procedure

Sample size was determined by single population proportion formula by considering the following assumptions. Proportion of respectful delivery care in Ethiopia from previous study was 64% [13], 95% of confidence level and allowed margin of error 5%.

$$\begin{aligned} {\text{n}} & = \frac{{\left( {{\text{Z}}\upalpha /2} \right)^{2} p\left( {1 - p} \right)^{{}} }}{{{\text{d}}^{2} }} \\ {\text{n}} & = \frac{{1.96^{2} \times 0.64\left( {1 - 0.64} \right)}}{{ \left( {0.05} \right)^{2} }} = 354 \\ \end{aligned}$$

**Fig. 1:**
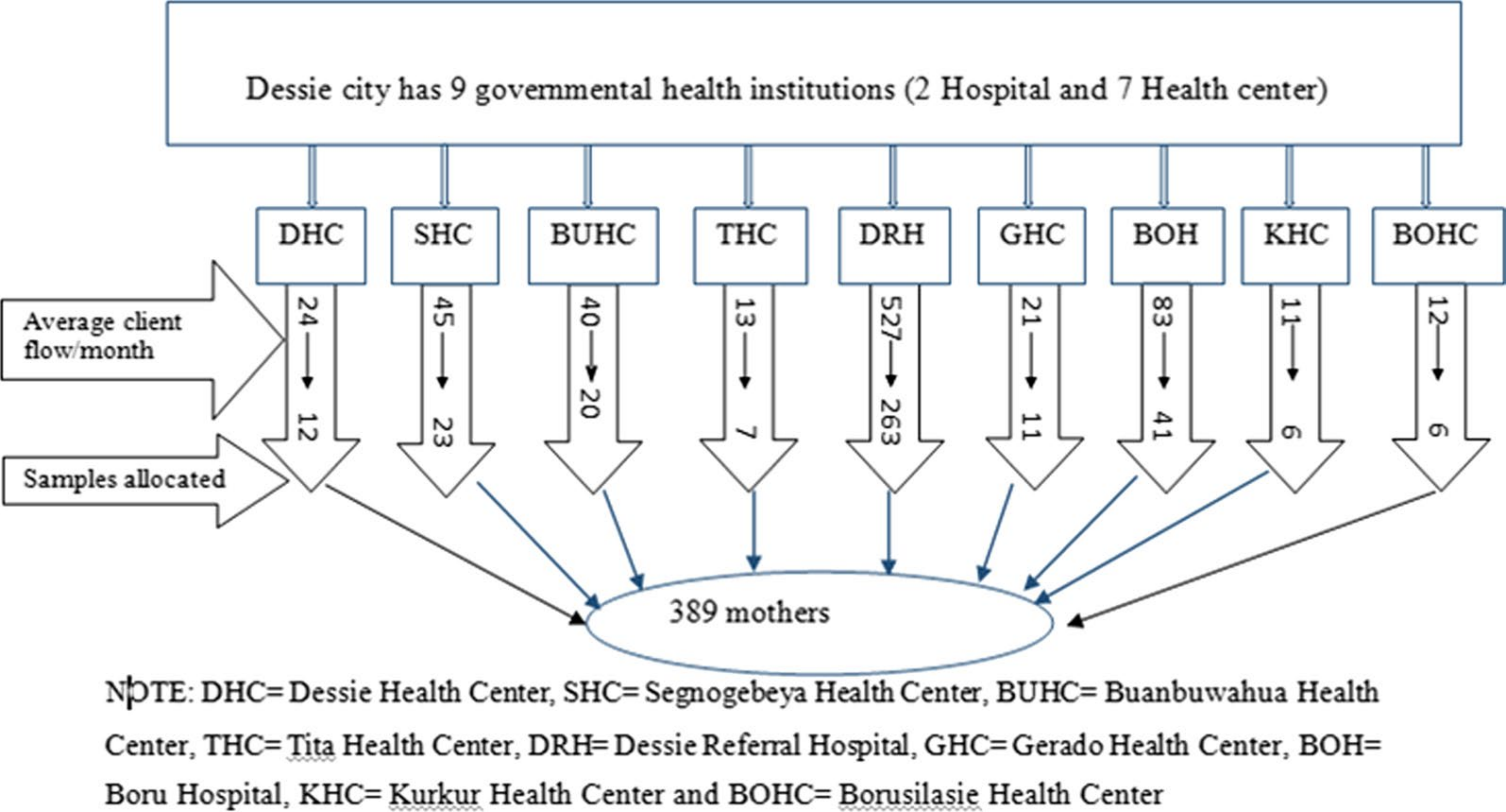
Schematic diagram showing sampling procedure for study on respectful delivery care and associated factors among mothers delivered in public health facilities of Dessie city, Ethiopia, 2018

Adding 10% for non-response the final sample size was n = 390. The sample size was also calculated based on the second objective for independent variables. By taking postpartum complication as an independent variable, percent of outcome in unexposed 69.3%, percent of outcome in exposed 78%, adjusted odds ratio 2, power 80%, confidence level 95%, and adding 10% non-response rate the sample size was 383. But, the sample size calculated using a single population proportion was greater than the one calculated for the factor so, the final sample size was (n = 390). The samples were initially proportionately allocated for each health facility based on their average number of delivery per month. Then, women were selected by systematic sampling procedure based on client flow and interviewed at the time of discharge (Fig. 1).

### Operational definition

Respectful delivery care was recorded as a dichotomous (yes or no) using a checklist that includes thirteen items as a desirable action of health providers. If a woman answered ‘yes’ for all of the thirteen questions, she was respected, otherwise disrespected [26, 36–39].

Wealth index: This is a composite variable that was calculated for urban and rural separately and it includes the following classification [35].

- Lowest: Includes women whose wealth was less than or equal to 20 percentile.
- Low: Includes women whose wealth was ranged 21 to 40 percentile.
- Medium: Includes women whose wealth was ranged 41 to 60 percentile.
- High: Includes women whose wealth was ranged 61 to 80 percentile.
- Highest: Includes women whose wealth was ranged 81 to 100 percentile.

Maternal complication: When a woman developed one or more of the following; post-partum hemorrhage, antepartum hemorrhage, chorioamnionites, preeclampsia, eclampsia, severe oligohydramnios, severe polyhydramnios, 3^rd^, and 4^th^-degree tear and abnormal adherent placenta/retained placenta, and any other complications [40]. Neonatal complication: If a neonate developed one or more of the following; mal-presentation, cord compression, distress, and any other complication to the newborn [40].

### Data collection tools, procedures, quality control, and analysis

The questionnaires were developed by reviewing the result of different studies and the national Compassionate, Respectful and Caring (CRC) guidelines [12, 13, 41]. The women in the labour ward were asked for the interview at the time of discharge for their valuable information and the data was filled in the questionnaire. Some of the questionnaires were also filled by observing the delivery card of mothers. Two BSc public health supervisors and six BSc nurses were employed as data collectors and they were doing in another health facility. The questionnaire was developed in English language and translated to Amharic again back-translated to English to check its consistency. Supervisors and data collectors were trained on the objective of the study, how to approach participants, and take informed consent. The tool was pre-tested on 5% samples in Kombolcha health center coming for the same service before entering the actual data collection and necessary modification was done according to the result of the pretest. The checklists used for measuring the outcome variable was checked for their reliability (Cronbach’s alpha value = 0.717) and the data were also continuously checked by supervisors and principal investigator.

The data were coded and entered into Epi-Data version 3.1 and exported to Stata/SE 14 for analysis. The result was presented using texts, frequency, percentage, and graph. First bi-variable binary logistic regression analysis was done and those variables with a p-value less than 0.2 were entered into multiple logistic regression to control confounding. Multi-colinearity between independent variables was checked using variance inflation factor as well as standard error and there is no multi-colinearity. Hosmer–Lemeshow test was used to check for model fitness and it was not significant (P-value = 0.791). In the final model, those variables with a p-value less than 0.05 were considered statistically significant. Odds ratio (OR) along with 95% confidence interval (CI) was estimated to measure the strength and direction of the association respectively.

## Results

### Socio-demographic characteristics of respondents

Three hundred ninety delivered women were participated, making the response rate of 99.7%. More than half (63.2%) were aged between 20 and 29 years. One hundred eighty (46.2%) were educated to secondary and above whereas 94 (24.1%) were not formally educated. About two-thirds of (68.9%) mothers were Muslims and 13 (3.3%) were protestants. In terms of ethnicity and occupation, 366 (94.1%) were Amhara and 262 (67.4%) were housewives (Table 1).

**Table 1:** Socio-demographic characteristics of mothers who delivered in health facilities of Dessie city, Ethiopia, 2018

Variables	Category	Frequency	Percentage
Age in years	< 20 years	15	3.85
	20–29 years	246	63.24
	30–40 years	128	32.91
Place of residence	Rural	131	33.67
	Urban	258	66.33
Marital status	Single	11	2.80
	Married	378	97.20
Educational status	Can’t read and write	53	13.60
	Can read and write only	41	10.50
	Primary	115	29.60
	Secondary	135	34.70
	College and above	45	11.60
Religion	Orthodox	108	27.80
	Muslim	268	68.90
	Others^a^	13	3.30
Ethnicity	Amhara	366	94.10
	Oromo	14	3.60
	Others^b^	9	2.30
Occupation	Government employee	38	9.80
	Merchant	32	8.20
	Private employee	12	3.10
	Housewife	262	67.30
	Others^c^	45	11.60
Wealth index	Lowest	78	20.10
	Low	77	19.80
	Medium	80	20.50
	High	76	19.50
	Highest	78	20.10

^a^Protestant and Catholic, ^b^Tigrie and Afar and ^c^student and jobless

### Proportion of respectful delivery care

**Fig. 2:**
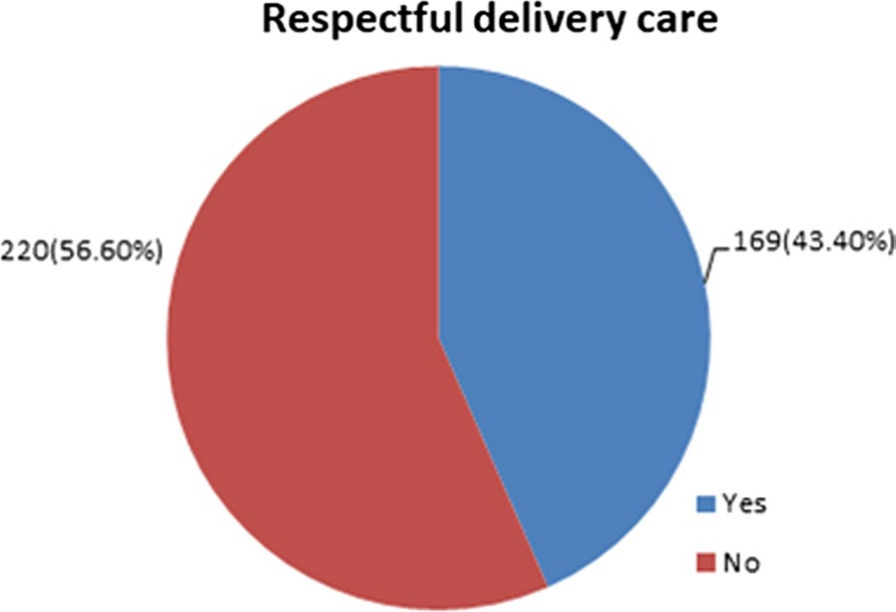
Over all proportion of respectful delivery care among mothers delivered in public health facilities of Dessie city, Ethiopia, 2018

The overall proportion of respectful delivery care among mothers who delivered in health facilities of Dessie city was 43.4% [95% CI (39.1, 47.6)] (Fig. 2). It was found to be 34.9% in hospitals and 74.1 in a health centers.

### Factors associated with respectful delivery care

The multivariable analysis result showed that the educational status of mothers, delivery time, and any fetal and/or maternal complications was found to have a statistical association with respectful delivery care. The odds of obtaining respectful delivery care for mothers educated up to grade 9–12 were almost 3 times higher as compared to mothers who couldn’t read and write [AOR = 2.87, 95% CI (1.18, 7.01)]. Those mothers who gave birth during day time were 2 times greater in receiving respectful delivery care than mothers who gave birth during night time [AOR = 2.23, 95% CI (1.30, 3.82)]. The odds of getting respectful delivery care for mothers who gave birth in a health center were 3.2 times more likely than those mothers who gave birth in a hospital [AOR = 3.22, 95% CI (1.61, 6.46)]. The odds of getting respectful delivery care were 50% less likely for mothers who faced any complication to her and/or her neonate as compared to mothers who didn’t face it [AOR = 0.50, 95% CI (0.27, 0.94)] (Table 2).

**Table 2:** Bivariable and multivariable logistic regression for respectful delivery care among mothers delivered in health facilities of Dessie city, 2018

Variables	Category	RDC	COR (95% CI)	AOR (95% CI)	
		Yes	No	n = 389	n = 371
Place of residence	Rural	41	90	1	1
	Urban	128	130	2.161 (1.388, 3.365)	1.446 (0.793, 2.639)
Educational status	Can’t read & write	12	41	1	1
	Read & write	13	28	1.586 (0.632, 3.981)	1.306 (0.437, 3.902)
	Primary	52	63	2.820 (1.345, 5.915)	2.156 (0.878, 5.296)
	Secondary	75	60	4.271 (2.064, 8.839)	2.871 (1.177, 7.005)*
	College & above	17	28	2.074 (0.859, 5.009)	0.499 (0.124, 2.011)
Occupation	Housewife	114	148	1	1
	Merchant	17	15	1.471 (0.705, 3.072)	1.025 (0.428, 2.457)
	Private employee	7	5	1.818 (0.562, 5.876)	1.484 (0.330, 6.675)
	Gov’t employee	20	18	1.442 (0.729, 2.853)	3.190 (0.916, 11.103)
	Others	11	34	0.42 (0.204, 0.865)	0.648 (0.263, 1.598)
Mode of delivery	SVD	109	100	1	1
	Episiotomy	24	31	0.71 (0.391, 1.292)	1.053 (0.506, 2.193)
	Instrumental	9	28	0.295 (0.133, 0.655)	1.193 (0.411, 3.459)
	C/S	27	61	0.406 (0.239, 0.689)	1.636 (0.680, 3.936)
Delivery time	Night	53	115	1	1
	Day	116	105	2.397 (1.577, 3.645)	2.225 (1.296, 3.820)**
Length of stay in health facility	< 1 day	112	95	1	1
	2–3 days	38	80	0.403 (0.251, 0.647)	1.091 (0.563, 2.116)
	> 4 days	19	45	0.358 (0.169, 0.654)	1.136 (0.427, 3.024)
Type of health facility	Hospital	106	198	1	1
	Health center	63	22	5.35 (3.12, 9.18)	3.22 (1.61, 6.46)**
Stage of labor arrived	First stage	133	150	1	1
	Second stage	31	56	0.624 (0.380, 1.026)	0.865 (0.466, 1.604)
Fetal/maternal complication	No	128	106	1	1
	Yes	41	114	0.298 (0.162, 0.462)	0.503 (0.268, 0.944)**
Referred	No	119	86	1	1
	Yes	50	134	0.27 (0.176, 0.413)	0.669 (0.347, 1.288)
Outcome of pregnancy	Alive	162	191	1	1
	Died	7	29	0.285 (0.121, 0.667)	0.415 (0.128, 1.345)
Time from labour started to reach health facility	< 1 h	40	34	1	1
	1–2 h	32	34	0.8 (0.411, 1.555)	0.673 (0.304, 1.493)
	> 2 h	92	139	0.563 (0.332, 0.954)	1.293 (0.650, 2.571)

The bold indicates the variable was statistically significant at α value of 0.05

*AOR* adjusted odds ratio, *COR* crude odds ratio, Others student and jobless, *RDC* respectful delivery care, *SVD* spontaneous vaginal delivery, *C/S* Caesarian Section, 1—reference, *—(P-value < 0.05) and **—(P-value < 0.01) in multivariable analysis respectively

## Discussion

In this study, the proportion of respectful delivery care was 43.4% and variables like time of delivery, maternal education, and occurrence of any maternal and/or fetal complication were significantly associated with respectful delivery care. The finding of this study is in line with a study conducted in Malawi (41.8%) [11]. But, it is lower than the previous study conducted in four regions of Ethiopia (64%) [41]. It is also lower than previous studies conducted in Kenya (80%) [42], Tanzania (85%) [43], and Nigeria (98%) [10]. The possible reason for this discrepancy may be due to time variation and differences in socio-cultural contexts of the study settings. Moreover, those previous studies were either observational or repeated client exit interviews whereas this study was only client exit interviews during immediate postpartum.

But, it is higher than another study conducted in Addis Ababa, Ethiopia (22%) [12], and India (22.7%) [36]. The discrepancy may be due to that the previous studies were used twenty-three items checklist to measure respectful delivery care but, this study used thirteen items. This implies that significant numbers of women were facing disrespect and abuse delivery care in the study setting. So, home delivery will persist high as far as respectful delivery care is not present in the groud and maternal and neonatal mortality will not be decreased as what is expected.

The higher educational status of mothers was positively associated with respectful delivery care. In contrast to this finding, studies conducted in Tanzania stated that educated women were more likely to experience disrespect [26]. The possible reason for this variation may be due to variation in the context like socio-demographic and cultural factors may attribute for the difference in the above two research’s. The possible justification for this association might be due to the reason that those educated mothers might understand their rights better than non-educated (counterparts) to follow physicians' orders.

This study revealed a significant negative relationship between complications and mothers’ respect. This finding is in line with studies conducted in Ethiopia [12], Tanzania [26], and India [36]. The possible reason for this association might be that complicated labour requires frequent and careful follow-up and monitoring that makes the provider more tiresome and keeping all her rights may endanger her life, especially during emergencies.

The finding also demonstrated that daytime childbirth was associated with respectful delivery care. The result of this study is similar to the previous study conducted in Ethiopia [13]. It is also similar to a study conducted in Kenya [44]. This might be due to that health providers during the nighttime may be disturbed and they may not act as normal because they awake from sleep. Additionally, the nighttime health workers might be overburdened by high numbers of client flow because of an incongruent number of providers assigned.

Type of health facility is also another significantly associated variable to respectful delivery care. Those mothers who gave birth in health centers were three times more likely to be respected than those mothers who gave birth in hospitals. The finding of the study is similar to the studies conducted in Ethiopia [13, 41]. It is also similar to a study conducted in Malawi [11]. The possible reason for this association may be due to the fact that high client flow in hospitals may make the providers become very busy and highly burdened [45, 46].

Despite this study addressed respectful delivery care quantitativelty, it is not without limitations. As the outcome was measured indirectly from mothers, this may make the study prone to recall bias since women may not be perfect in remembering or told in what way they were treated. Lastly, the study was only on governmental health facilities that may not represent the experiences of private health facilities delivery care.

## Conclusions

Even though CRC in Ethiopia is a current governmental agenda, the proportion of respectful delivery care is still lower as compared to the WHO recommendation. The study indicated that any maternal/newborn complication, time of delivery, and maternal education were associated with respectful delivery care. Respectful delivery care can be improved by giving especial emphasis to nighttime delivery, facing any complication to her and/or her child, giving birth in a hospital, and on uneducated mothers. The findings suggested that continue the pre-started CRC strategies, especially nighttime health workers to improve women’s experience of respectful delivery care. Women empowerment through education could also be the recalled intervention for respectful care. All delivery attendants should serve clients respectfully irrespective of mothers' educational status, maternal/newborn complications, and delivery time.

## Supplementary Information

**Additional file 1:** The dataset used or analysed in the current study.

**Additional file 2:** The English version questionaries.

## Data Availability

The datasets used and/or analyzed during this study are attached with the manuscript (see Additional file 1).

## Abbreviations

AIDS: Acquired immune deficiency syndrome
CRC: Compassionate respectful and caring
HIV: Human immunodeficiency virus
MHCPs: Maternal Health Care Provider
WHO: World Health Organization
