# Supplementary material for: Respectful delivery care and associated factors among mothers delivered in public health facilities of Dessie city, Northeast Ethiopia: a cross-sectional study
Source: BMC Womens Health. 2022 Apr 21;22:127. doi: 10.1186/s12905-022-01713-1 (PMC9026676; doi:10.1186/s12905-022-01713-1)
Supplement: Supplementary file 1 — Additional file 1: The dataset used or analysed in the current study. [file 12905_2022_1713_MOESM1_ESM.pdf]

Q100	Q101	Q102	Q103	Q104	Q105	Q106	Q107	Q201
1	22	urban	married	grade1to8	orthodox	amhara	housewife	yes
2	20	rural	married	grade9to12	muslim	amhara	housewife	yes
3	22	urban	married	grade9to12	orthodox	amhara	governmer	yes
4	28	urban	married	collegeand	muslim	amhara	governmer	yes
5	27	urban	married	onlyreadan	muslim	amhara	housewife	yes
6	21	urban	married	grade1to8	orthodox	amhara	housewife	yes
7	33	urban	married	collegeand	orthodox	amhara	governmer	yes
8	30	urban	married	cannotreac	orthodox	amhara	merchant	yes
9	30	urban	married	collegeand	orthodox	amhara	governmer	yes
10	30	rural	single	cannotreac	muslim	amhara	housewife	yes
11	22	rural	married	grade9to12	muslim	amhara	housewife	yes
12	30	urban	married	collegeand	muslim	amhara	governmer	yes
13	30	urban	married	onlyreadan	muslim	oromo	housewife	yes
14	24	urban	married	grade9to12	orthodox	amhara	housewife	yes
15	23	urban	married	collegeand	muslim	amhara	student	yes
16	25	rural	married	collegeand	orthodox	tigre	housewife	yes
17	28	urban	married	grade9to12	orthodox	amhara	housewife	yes
18	32	urban	married	grade9to12	muslim	oromo	merchant	yes
19	25	urban	married	collegeand	orthodox	amhara	housewife	yes
20	20	rural	married	cannotreac	muslim	amhara	student	yes
21	30	urban	married	collegeand	protestant	amhara	merchant	yes
22	20	rural	married	grade1to8	muslim	amhara	merchant	yes
23	30	urban	married	grade1to8	muslim	amhara	housewife	yes
24	35	urban	married	grade9to12	muslim	amhara	housewife	yes
25	24	urban	married	grade1to8	muslim	amhara	housewife	yes
26	27	urban	married	grade1to8	orthodox	amhara	housewife	yes
27	40	rural	married	cannotreac	muslim	amhara	student	yes
28	30	urban	married	grade1to8	orthodox	amhara	housewife	yes
29	20	rural	married	grade1to8	muslim	amhara	housewife	yes
30	28	urban	married	grade1to8	muslim	oromo	merchant	yes
31	32	urban	married	grade9to12	muslim	amhara	housewife	yes
32	24	urban	married	collegeand	muslim	amhara	governmer	yes
33	20	rural	married	cannotreac	muslim	amhara	housewife	yes
34	25	urban	married	grade9to12	orthodox	amhara	housewife	yes
35	24	urban	married	grade9to12	orthodox	amhara	student	yes
36	26	rural	married	grade9to12	muslim	amhara	housewife	yes
37	24	rural	married	collegeand	muslim	amhara	governmer	yes
38	40	urban	married	cannotreac	orthodox	amhara	housewife	yes
39	22	rural	married	grade1to8	muslim	oromo	housewife	yes
40	28	urban	married	collegeand	muslim	amhara	governmer	yes
41	27	rural	married	grade1to8	muslim	amhara	housewife	no
42	22	urban	married	collegeand	muslim	amhara	housewife	yes
43	29	urban	married	collegeand	muslim	amhara	governmer	yes
44	28	urban	married	collegeand	orthodox	amhara	student	yes
45	21	rural	married	cannotreac	muslim	amhara	housewife	no
46	24	urban	married	collegeand	muslim	amhara	housewife	no
47	20	rural	married	grade1to8	muslim	amhara	housewife	no
48	28	urban	married	collegeand	muslim	amhara	student	no
49	23	rural	married	collegeand	muslim	amhara	governmer	yes
50	22	urban	married	grade1to8	orthodox	amhara	housewife	no
51	28	rural	married	grade9to12	muslim	amhara	housewife	no

52	31	urban	married	cannotreac	muslim	amhara	housewife	yes
53	24	urban	married	collegeand	orthodox	amhara	housewife	no
54	30	urban	married	collegeand	orthodox	amhara	privateemp	no
55	31	urban	married	cannotreac	muslim	amhara	housewife	no
56	23	urban	married	collegeand	orthodox	amhara	student	yes
57	23	urban	married	grade1to8	muslim	amhara	housewife	no
58	27	urban	married	grade9to12	orthodox	amhara	housewife	yes
59	22	urban	married	grade1to8	orthodox	amhara	housewife	yes
60	27	urban	married	onlyreadan	orthodox	amhara	housewife	yes
61	24	urban	married	grade1to8	muslim	amhara	housewife	yes
62	28	urban	married	grade9to12	muslim	amhara	housewife	yes
63	22	urban	married	grade9to12	muslim	amhara	housewife	yes
64	29	urban	married	onlyreadan	muslim	amhara	housewife	yes
65	25	urban	married	grade9to12	muslim	amhara	housewife	yes
66	30	urban	married	grade9to12	orthodox	amhara	housewife	yes
67	25	rural	married	cannotreac	orthodox	amhara	governmer	yes
68	24	urban	married	grade9to12	muslim	amhara	housewife	yes
69	35	rural	married	cannotreac	muslim	amhara	governmer	yes
70	27	urban	married	grade9to12	muslim	amhara	housewife	yes
71	26	urban	married	collegeand	muslim	amhara	housewife	yes
72	28	urban	married	grade9to12	orthodox	amhara	housewife	yes
73	25	urban	married	grade9to12	muslim	amhara	housewife	yes
74	30	urban	married	grade1to8	muslim	amhara	housewife	yes
75	37	urban	married	grade9to12	muslim	oromo	housewife	yes
76	26	urban	single	grade1to8	muslim	amhara	privateemp	yes
77	22	urban	married	collegeand	muslim	amhara	student	yes
78	26	urban	married	grade9to12	orthodox	amhara	governmer	yes
79	25	urban	single	onlyreadan	muslim	amhara	merchant	yes
80	30	rural	married	cannotreac	muslim	amhara	housewife	yes
81	25	rural	married	grade9to12	muslim	amhara	housewife	yes
82	25	urban	married	grade9to12	muslim	amhara	housewife	yes
83	25	rural	married	grade1to8	muslim	amhara	housewife	yes
84	26	rural	married	grade1to8	muslim	amhara	housewife	yes
85	26	rural	married	grade9to12	muslim	amhara	housewife	yes
86	39	rural	married	cannotreac	muslim	amhara	student	yes
87	30	urban	married	collegeand	muslim	amhara	governmer	yes
88	35	urban	married	grade1to8	protestant	amhara	housewife	yes
89	25	urban	married	grade1to8	muslim	amhara	housewife	yes
90	27	rural	married	grade1to8	muslim	amhara	housewife	yes
91	27	urban	married	grade9to12	muslim	amhara	housewife	yes
92	20	rural	married	grade1to8	muslim	oromo	housewife	yes
93	23	rural	married	onlyreadan	muslim	amhara	housewife	yes
94	22	urban	married	grade1to8	muslim	amhara	merchant	yes
96	24	rural	married	grade9to12	muslim	amhara	privateemp	yes
96	28	urban	married	cannotreac	muslim	amhara	housewife	yes
97	34	rural	married	cannotreac	muslim	amhara	housewife	yes
98	26	urban	married	grade9to12	muslim	amhara	housewife	yes
99	27	urban	single	cannotreac	muslim	amhara	housewife	yes
100	27	urban	married	onlyreadan	muslim	amhara	housewife	no
101	19	rural	married	grade1to8	muslim	amhara	housewife	yes
102	35	urban	married	grade9to12	muslim	amhara	merchant	yes
103	32	urban	married	grade9to12	muslim	amhara	merchant	yes

104	30	urban	married	cannotreac	orthodox	amhara	merchant	yes
105	30	urban	married	grade9to12	muslim	amhara	housewife	yes
106	32	rural	married	onlyreadan	muslim	amhara	merchant	yes
107	28	urban	married	grade9to12	muslim	amhara	housewife	yes
108	25	rural	married	grade1to8	muslim	amhara	housewife	yes
109	22	urban	married	grade1to8	muslim	amhara	housewife	yes
110	19	urban	married	grade9to12	protestant	amhara	merchant	yes
111	25	rural	married	cannotreac	muslim	amhara	housewife	yes
112	26	urban	married	collegeand	muslim	amhara	housewife	yes
113	40	rural	married	cannotreac	muslim	amhara	housewife	yes
114	28	urban	married	grade9to12	muslim	amhara	housewife	no
115	26	urban	married	grade9to12	muslim	amhara	governmer	yes
116	33	urban	married	grade1to8	muslim	amhara	housewife	yes
117	28	urban	married	grade9to12	muslim	amhara	housewife	no
118	22	urban	married	grade9to12	muslim	amhara	housewife	no
119	25	urban	married	grade1to8	muslim	amhara	housewife	no
120	28	urban	married	collegeand	orthodox	amhara	privateemp	yes
121	24	rural	married	cannotreac	muslim	amhara	student	yes
122	30	rural	married	cannotreac	orthodox	amhara	student	yes
123	20	urban	married	grade1to8	muslim	amhara	housewife	yes
124	28	rural	married	grade9to12	muslim	amhara	privateemp	yes
125	27	urban	married	grade1to8	muslim	amhara	housewife	yes
126	24	rural	married	grade1to8	orthodox	amhara	housewife	no
127	27	urban	married	onlyreadan	orthodox	amhara	privateemp	yes
128	30	urban	married	grade1to8	protestant	oromo	merchant	no
129	27	urban	married	grade9to12	protestant	amhara	privateemp	no
130	39	urban	single	onlyreadan	muslim	oromo	governmer	yes
131	27	urban	married	grade1to8	muslim	amhara	housewife	yes
132	30	rural	married	cannotreac	muslim	oromo	student	no
133	21	rural	married	onlyreadan	muslim	amhara	housewife	yes
134	30	rural	married	cannotreac	orthodox	amhara	housewife	yes
135	36	rural	married	onlyreadan	muslim	amhara	housewife	no
136	36	rural	married	onlyreadan	muslim	amhara	housewife	no
137	25	urban	married	grade1to8	muslim	amhara	housewife	yes
138	30	urban	married	cannotreac	muslim	amhara	housewife	yes
139	27	rural	married	grade9to12	muslim	amhara	housewife	yes
140	24	rural	married	grade9to12	orthodox	amhara	housewife	yes
141	30	urban	married	cannotreac	orthodox	amhara	housewife	yes
142	30	rural	married	cannotreac	muslim	amhara	student	yes
143	25	rural	married	grade1to8	muslim	amhara	student	yes
144	29	urban	married	grade9to12	muslim	amhara	housewife	yes
145	23	urban	married	grade9to12	orthodox	amhara	housewife	yes
146	25	urban	married	grade1to8	muslim	amhara	housewife	yes
147	27	urban	married	grade1to8	muslim	amhara	housewife	yes
148	24	urban	married	grade9to12	muslim	amhara	housewife	yes
149	37	rural	married	grade1to8	muslim	amhara	housewife	yes
150	24	rural	married	cannotreac	orthodox	amhara	housewife	yes
151	24	rural	married	cannotreac	muslim	amhara	housewife	no
152	29	urban	married	grade1to8	muslim	amhara	housewife	yes
153	20	rural	married	grade1to8	orthodox	amhara	housewife	no
154	30	urban	married	grade9to12	orthodox	amhara	housewife	yes
155	27	urban	married	collegeand	muslim	amhara	governmer	yes

156	25	urban	married	grade9to12	orthodox	amhara	student	yes
157	39	rural	married	cannotreac	muslim	amhara	housewife	yes
158	23	urban	married	grade9to12	orthodox	tigre	student	yes
159	32	urban	married	grade9to12	muslim	amhara	housewife	yes
160	34	urban	married	grade1to8	muslim	amhara	housewife	yes
161	34	urban	married	grade9to12	protestant	amhara	housewife	yes
162	29	urban	married	grade1to8	muslim	amhara	merchant	yes
163	23	urban	married	grade9to12	orthodox	tigre	housewife	yes
164	22	urban	married	cannotreac	orthodox	amhara	housewife	yes
165	25	rural	married	grade1to8	muslim	amhara	housewife	yes
166	24	urban	married	onlyreadan	muslim	amhara	merchant	yes
167	21	urban	married	grade9to12	orthodox	amhara	housewife	yes
168	33	urban	married	collegeand	muslim	amhara	governmer	yes
169	34	urban	married	collegeand	orthodox	amhara	governmer	yes
170	22	urban	married	collegeand	muslim	amhara	governmer	yes
171	31	rural	married	cannotreac	muslim	amhara	student	yes
172	35	urban	married	grade1to8	muslim	amhara	housewife	yes
173	23	urban	married	grade9to12	muslim	amhara	housewife	yes
174	22	urban	married	grade9to12	orthodox	oromo	governmer	yes
175	24	urban	married	grade9to12	orthodox	amhara	housewife	yes
176	30	rural	married	cannotreac	muslim	amhara	housewife	yes
177	24	urban	married	cannotreac	orthodox	amhara	housewife	yes
178	23	urban	married	grade1to8	muslim	amhara	housewife	yes
179	28	urban	married	grade9to12	muslim	amhara	housewife	yes
180	32	urban	married	grade1to8	muslim	amhara	housewife	yes
181	18	urban	married	grade9to12	muslim	amhara	housewife	yes
182	18	urban	married	grade1to8	muslim	amhara	housewife	yes
183	28	urban	married	grade9to12	orthodox	amhara	housewife	yes
184	26	urban	married	grade9to12	orthodox	amhara	housewife	yes
185	25	urban	married	collegeand	orthodox	amhara	governmer	yes
186	25	rural	married	grade9to12	muslim	amhara	housewife	yes
187	24	urban	married	collegeand	orthodox	amhara	governmer	yes
188	20	urban	married	grade9to12	muslim	amhara	housewife	yes
189	26	urban	married	collegeand	protestant	amhara	governmer	no
190	29	urban	married	grade9to12	muslim	amhara	housewife	yes
191	33	rural	married	grade1to8	muslim	amhara	housewife	yes
192	22	urban	married	grade1to8	muslim	amhara	housewife	yes
193	22	urban	married	grade9to12	muslim	amhara	housewife	yes
194	18	urban	married	grade1to8	muslim	amhara	housewife	yes
195	22	urban	single	grade1to8	muslim	amhara	privateemp	yes
196	35	urban	married	cannotreac	muslim	amhara	housewife	yes
197	34	urban	married	grade9to12	muslim	amhara	merchant	yes
198	36	urban	married	onlyreadan	protestant	amhara	housewife	yes
199	26	urban	married	grade1to8	orthodox	amhara	housewife	yes
200	25	rural	married	grade9to12	muslim	amhara	housewife	yes
201	30	rural	married	grade1to8	muslim	amhara	housewife	yes
202	30	urban	married	cannotreac	muslim	amhara	housewife	no
203	23	rural	married	grade1to8	muslim	amhara	housewife	yes
204	23	rural	married	grade9to12	muslim	amhara	housewife	yes
205	26	urban	married	collegeand	orthodox	amhara	governmer	yes
206	30	urban	married	grade9to12	muslim	amhara	housewife	yes
207	20	rural	married	grade1to8	muslim	amhara	housewife	yes

208	30	rural	married	cannotreac	muslim	amhara	housewife	yes
209	21	rural	married	grade1to8	muslim	amhara	student	yes
210	26	rural	married	onlyreadan	muslim	amhara	housewife	yes
211	30	urban	married	grade1to8	muslim	amhara	housewife	yes
212	18	urban	single	grade1to8	muslim	amhara	student	yes
213	37	rural	married	onlyreadan	muslim	amhara	housewife	yes
214	39	urban	single	grade1to8	protestant	tigre	housewife	yes
215	22	rural	married	grade9to12	muslim	amhara	housewife	yes
216	25	urban	married	grade9to12	muslim	amhara	housewife	yes
217	30	urban	married	grade9to12	orthodox	amhara	student	yes
218	27	rural	married	grade9to12	muslim	amhara	housewife	yes
219	38	rural	married	onlyreadan	orthodox	amhara	housewife	yes
220	28	urban	married	grade9to12	muslim	amhara	merchant	yes
221	22	urban	married	grade1to8	muslim	amhara	privateemp	yes
222	38	urban	married	cannotreac	muslim	amhara	housewife	yes
223	19	urban	single	grade9to12	protestant	amhara	student	yes
224	28	urban	married	grade1to8	muslim	amhara	housewife	yes
225	26	rural	married	grade9to12	orthodox	amhara	housewife	yes
226	25	rural	married	cannotreac	orthodox	amhara	student	yes
227	32	rural	married	cannotreac	orthodox	amhara	student	no
228	32	rural	married	grade9to12	orthodox	amhara	student	yes
229	31	rural	married	cannotreac	orthodox	amhara	student	yes
230	28	urban	married	collegeand	orthodox	amhara	governmer	yes
231	30	urban	married	grade9to12	orthodox	amhara	housewife	yes
232	27	urban	married	collegeand	orthodox	amhara	privateemp	no
233	25	rural	married	grade9to12	orthodox	amhara	housewife	no
234	31	rural	married	onlyreadan	orthodox	amhara	housewife	no
235	32	urban	married	cannotreac	muslim	amhara	housewife	yes
236	24	urban	married	grade9to12	orthodox	amhara	housewife	yes
237	24	urban	single	grade9to12	orthodox	amhara	student	yes
238	40	urban	married	cannotreac	muslim	amhara	housewife	yes
239	30	urban	married	collegeand	orthodox	amhara	governmer	yes
240	28	urban	married	collegeand	muslim	amhara	governmer	yes
241	29	rural	married	onlyreadan	muslim	amhara	housewife	yes
242	24	urban	married	grade9to12	muslim	amhara	student	yes
243	22	urban	married	grade9to12	protestant	amhara	housewife	yes
244	29	urban	married	grade1to8	muslim	amhara	merchant	yes
245	27	rural	married	cannotreac	muslim	amhara	housewife	yes
246	19	rural	married	onlyreadan	muslim	amhara	housewife	yes
247	34	rural	married	cannotreac	orthodox	oromo	housewife	yes
248	38	rural	married	onlyreadan	muslim	amhara	housewife	yes
249	31	urban	married	grade1to8	muslim	amhara	housewife	yes
250	30	urban	married	grade9to12	muslim	amhara	housewife	yes
251	30	rural	married	cannotreac	muslim	amhara	student	yes
252	24	urban	married	collegeand	muslim	amhara	governmer	yes
253	28	rural	married	grade9to12	muslim	amhara	merchant	yes
254	26	rural	married	onlyreadan	orthodox	amhara	student	yes
255	23	urban	married	grade9to12	muslim	amhara	housewife	yes
256	34	urban	married	grade9to12	muslim	amhara	housewife	yes
257	39	urban	married	grade9to12	muslim	amhara	housewife	yes
258	28	rural	married	grade1to8	muslim	amhara	housewife	yes
259	34	rural	married	cannotreac	muslim	amhara	student	yes

260	23	rural	married	onlyreadan	muslim	amhara	housewife	yes
261	26	urban	married	grade1to8	muslim	amhara	merchant	yes
262	29	urban	married	grade1to8	orthodox	amhara	student	yes
263	39	urban	married	grade9to12	muslim	amhara	housewife	yes
264	38	urban	married	grade9to12	protestant	amhara	housewife	yes
265	33	urban	married	collegeand	orthodox	amhara	governmer	yes
266	38	urban	married	grade1to8	muslim	amhara	housewife	yes
267	28	urban	married	grade1to8	orthodox	amhara	housewife	yes
268	24	rural	married	grade9to12	muslim	amhara	student	yes
269	38	rural	married	cannotreac	orthodox	amhara	student	yes
270	32	urban	married	grade1to8	orthodox	tigre	merchant	yes
271	34	urban	married	grade1to8	muslim	amhara	housewife	no
272	27	urban	married	grade9to12	muslim	amhara	housewife	yes
273	23	urban	married	grade1to8	muslim	oromo	student	yes
274	19	urban	married	grade9to12	muslim	amhara	student	yes
275	35	urban	married	grade9to12	orthodox	amhara	housewife	yes
276	39	rural	married	onlyreadan	orthodox	amhara	housewife	yes
277	21	rural	married	grade1to8	muslim	amhara	student	yes
278	29	urban	married	grade9to12	muslim	tigre	housewife	yes
279	26	urban	married	grade9to12	orthodox	amhara	housewife	yes
280	19	urban	married	grade9to12	muslim	amhara	student	yes
280	32	urban	married	grade9to12	muslim	amhara	merchant	yes
282	19	urban	married	grade9to12	muslim	amhara	student	yes
283	21	urban	married	grade1to8	muslim	amhara	student	yes
284	37	urban	married	grade1to8	muslim	oromo	housewife	yes
285	24	urban	married	grade9to12	muslim	amhara	housewife	yes
286	29	rural	married	onlyreadan	orthodox	amhara	housewife	yes
287	26	rural	married	onlyreadan	muslim	amhara	housewife	yes
288	22	rural	married	grade9to12	orthodox	amhara	student	yes
289	30	urban	married	grade1to8	muslim	amhara	housewife	yes
290	28	urban	married	grade9to12	muslim	amhara	housewife	yes
291	31	urban	married	collegeand	orthodox	amhara	governmer	yes
292	32	urban	married	grade9to12	muslim	amhara	merchant	yes
293	37	rural	married	cannotreac	muslim	amhara	housewife	yes
294	39	urban	married	grade1to8	orthodox	amhara	housewife	yes
295	36	urban	married	grade1to8	muslim	amhara	housewife	yes
296	27	urban	married	grade9to12	muslim	amhara	merchant	yes
297	30	rural	married	grade1to8	orthodox	amhara	housewife	yes
298	35	urban	married	grade9to12	muslim	tigre	merchant	yes
299	32	urban	married	grade9to12	muslim	amhara	housewife	yes
300	34	rural	married	onlyreadan	muslim	amhara	housewife	yes
301	27	urban	married	grade9to12	muslim	amhara	housewife	yes
302	21	urban	married	grade1to8	muslim	amhara	housewife	yes
303	24	rural	married	grade9to12	muslim	amhara	housewife	yes
304	25	urban	single	cannotreac	orthodox	amhara	privateemp	yes
305	24	urban	married	collegeand	muslim	amhara	governmer	yes
306	30	urban	married	grade9to12	muslim	amhara	housewife	no
307	32	urban	married	onlyreadan	muslim	amhara	housewife	yes
308	20	rural	married	grade1to8	muslim	amhara	housewife	yes
309	28	rural	married	cannotreac	muslim	amhara	housewife	no
310	36	urban	married	onlyreadan	muslim	amhara	housewife	yes
311	28	urban	married	grade1to8	muslim	amhara	housewife	yes

312	28	urban	married	grade9to12	muslim	amhara	merchant	yes
313	28	urban	married	grade9to12	orthodox	amhara	housewife	yes
314	31	urban	married	grade1to8	muslim	amhara	merchant	yes
316	29	urban	married	grade9to12	orthodox	amhara	housewife	yes
316	30	urban	married	grade1to8	orthodox	amhara	housewife	yes
317	27	urban	married	grade9to12	muslim	amhara	merchant	yes
318	26	urban	married	collegeand	muslim	amhara	governmer	yes
319	24	urban	married	grade9to12	orthodox	amhara	governmer	yes
320	26	urban	married	grade1to8	muslim	amhara	housewife	yes
321	29	urban	married	grade9to12	muslim	amhara	housewife	yes
322	21	urban	married	grade1to8	muslim	amhara	student	yes
323	26	rural	married	grade9to12	muslim	amhara	merchant	yes
324	29	urban	married	collegeand	muslim	amhara	governmer	yes
325	30	urban	married	grade9to12	orthodox	amhara	housewife	yes
326	25	urban	married	grade9to12	muslim	amhara	student	yes
327	28	urban	married	grade9to12	orthodox	amhara	housewife	yes
328	19	urban	married	grade1to8	muslim	amhara	student	yes
329	28	urban	married	collegeand	muslim	amhara	governmer	yes
330	27	urban	married	grade9to12	orthodox	amhara	housewife	yes
331	32	urban	married	grade1to8	muslim	amhara	housewife	yes
332	28	urban	married	grade1to8	muslim	amhara	merchant	yes
333	31	rural	married	cannotreac	muslim	amhara	housewife	yes
334	27	urban	married	grade1to8	muslim	amhara	housewife	yes
335	24	urban	married	grade9to12	muslim	amhara	privateemp	no
336	35	rural	married	onlyreadan	muslim	amhara	housewife	yes
337	21	rural	married	grade9to12	muslim	amhara	housewife	no
338	24	rural	married	grade1to8	muslim	amhara	housewife	yes
339	28	urban	married	grade9to12	protestant	amhara	housewife	yes
340	34	rural	married	grade1to8	orthodox	amhara	housewife	yes
341	24	urban	married	grade1to8	orthodox	amhara	housewife	yes
342	19	urban	married	grade9to12	muslim	amhara	student	yes
343	28	urban	married	grade1to8	muslim	amhara	housewife	yes
344	26	urban	married	grade9to12	orthodox	amhara	housewife	yes
345	21	rural	married	grade1to8	muslim	amhara	housewife	yes
346	24	rural	married	grade1to8	muslim	amhara	housewife	yes
347	21	urban	married	grade1to8	muslim	amhara	merchant	yes
348	26	rural	married	onlyreadan	muslim	amhara	housewife	yes
349	32	rural	married	onlyreadan	muslim	amhara	housewife	yes
350	29	rural	married	onlyreadan	orthodox	amhara	housewife	yes
351	24	rural	married	grade1to8	muslim	amhara	housewife	yes
352	24	urban	married	grade9to12	muslim	amhara	housewife	yes
353	29	rural	married	onlyreadan	muslim	amhara	housewife	yes
354	31	rural	married	grade1to8	muslim	amhara	housewife	yes
355	32	urban	married	grade1to8	orthodox	amhara	housewife	yes
356	36	rural	married	grade1to8	muslim	amhara	housewife	yes
357	33	urban	married	grade1to8	orthodox	amhara	housewife	yes
358	28	rural	married	grade1to8	muslim	amhara	housewife	yes
359	23	urban	married	grade9to12	muslim	amhara	housewife	yes
360	29	urban	married	collegeand	muslim	amhara	governmer	yes
361	34	rural	married	onlyreadan	orthodox	amhara	housewife	yes
362	32	urban	married	grade9to12	muslim	amhara	housewife	yes
363	28	rural	married	onlyreadan	orthodox	amhara	housewife	yes

364	26	urban	married	grade1to8	muslim	amhara	housewife	yes
365	24	urban	married	grade9to12	muslim	amhara	housewife	yes
366	23	rural	married	grade1to8	muslim	amhara	housewife	yes
367	31	urban	married	grade1to8	orthodox	amhara	housewife	yes
368	28	rural	married	onlyreadan	muslim	amhara	housewife	yes
369	28	rural	married	cannotreac	muslim	tigre	student	yes
370	32	urban	married	grade9to12	muslim	amhara	housewife	yes
371	28	rural	married	onlyreadan	muslim	oromo	governmer	yes
372	29	urban	married	grade1to8	muslim	amhara	housewife	yes
373	28	rural	married	onlyreadan	orthodox	amhara	housewife	yes
374	32	urban	married	grade1to8	orthodox	amhara	housewife	yes
375	29	rural	married	cannotreac	orthodox	amhara	housewife	yes
376	30	urban	married	grade1to8	orthodox	amhara	merchant	yes
377	26	urban	married	grade9to12	muslim	amhara	housewife	yes
378	25	rural	married	grade1to8	orthodox	tigre	housewife	yes
379	36	rural	married	onlyreadan	orthodox	amhara	housewife	yes
380	28	rural	married	grade1to8	muslim	amhara	housewife	yes
381	22	rural	married	grade1to8	muslim	amhara	housewife	yes
382	26	rural	married	grade9to12	orthodox	amhara	housewife	yes
383	19	urban	married	grade9to12	muslim	amhara	housewife	yes
384	21	rural	married	grade1to8	muslim	amhara	housewife	yes
385	21	urban	married	grade9to12	muslim	amhara	housewife	yes
386	24	urban	married	grade9to12	muslim	amhara	housewife	yes
387	27	urban	married	grade9to12	muslim	amhara	housewife	yes
388	18	urban	married	grade1to8	muslim	amhara	housewife	yes
389	28	urban	married	grade9to12	muslim	amhara	housewife	yes

Q202	Q203	Q204	Q205	Q206	Q207	Q301	Q302	Q303
no	no	no	no	yes	nonreactive	2	yes	6
no	no	no	yes	yes	nonreactive	1	yes	4
no	no	yes	no	yes	nonreactive	1	yes	5
no	no	no	yes	yes	nonreactive	1	yes	3
no	no	yes	no	yes	nonreactive	2	yes	3
no	no	no	no	yes	nonreactive	1	yes	4
no	yes	yes	no	yes	reactive	3	yes	3
no	no	no	yes	yes	nonreactive	1	yes	3
no	no	no	no	yes	nonreactive	2	yes	4
yes	yes	yes	yes	yes	nonreactive	3	yes	3
no	no	no	yes	yes	nonreactive	2	yes	3
yes	no	no	yes	yes	nonreactive	2	yes	4
no	yes	yes	yes	yes	nonreactive	2	yes	2
no	no	no	no	yes	nonreactive	1	yes	3
no	no	yes	yes	yes	nonreactive	1	yes	7
no	no	yes	no	yes	nonreactive	1	yes	4
no	yes	no	no	yes	nonreactive	2	yes	4
no	no	yes	no	yes	nonreactive	2	yes	4
	no	yes	yes	yes	nonreactive	1	no	4
no	no	yes	yes	yes	nonreactive	2	yes	5
no	yes	yes	no	yes	nonreactive	2	yes	3
no	no	no	yes	yes	nonreactive	1	yes	5
no	no	no	yes	yes	nonreactive	4	yes	4
no	no	no	yes	yes	nonreactive	2	yes	6
no	no	yes	yes	yes	nonreactive	1	yes	3
no	no	yes	yes	yes	nonreactive	1	yes	4
no	no	yes	yes	yes	nonreactive	4	yes	2
yes	yes	no	yes	yes	nonreactive	2	yes	2
no	no	no	yes	yes	nonreactive	1	yes	4
no	no	yes	yes	yes	nonreactive	2	yes	3
no	yes	no	yes	yes	nonreactive	3	yes	4
no	no	yes	no	yes	nonreactive	1	yes	4
no	no	no	yes	yes	nonreactive	1	yes	4
no	no	yes	yes	yes	nonreactive	1	yes	4
no	no	no	yes	yes	nonreactive	1	yes	2
no	yes	yes	yes	yes	nonreactive	2	yes	4
no	yes	no	yes	yes	nonreactive	1	yes	4
no	yes	yes	yes	yes	nonreactive	2	yes	4
	no	no	yes	yes	nonreactive	1	no	
no	no	no	no	yes	nonreactive	2	yes	5
no	no	no	yes	yes	nonreactive	1	yes	4
no	no	yes	no	yes	nonreactive	1	yes	4
no	no	yes	no	yes	nonreactive	1	yes	4
no	no	no	no	yes	nonreactive	1	yes	3
no	no	no	yes	yes	nonreactive	1	yes	4
no	no	no	no	yes	nonreactive	1	yes	5
no	no	no	yes	yes	nonreactive	1	yes	1
no	yes	yes	yes	yes	nonreactive	2	yes	5
no	no	yes	no	yes	nonreactive	1	yes	4
no	no	no	no	no	nonreactive	2	yes	6
no	no	no	no	yes	nonreactive	3	yes	3

no	no	no	yes	yes	nonreactive	1	yes	3
no	yes	no	no	yes	nonreactive	2	yes	3
no	yes	no	no	yes	nonreactive	3	yes	3
no	no	no	yes	yes	nonreactive	1	yes	4
no	no	no	no	yes	nonreactive	1	yes	4
yes	no	no	no	yes	nonreactive	1	yes	5
no	no	no	no	yes	nonreactive	3	yes	3
no	no	yes	no	yes	nonreactive	1	yes	3
no	no	no	no	yes	nonreactive	2	yes	3
no	no	no	no	yes	nonreactive	1	yes	3
no	no	no	no	yes	nonreactive	2	yes	3
no	no	no	no	yes	nonreactive	1	yes	3
no	no	no	no	yes	nonreactive	4	yes	3
no	yes	yes	no	yes	nonreactive	2	yes	4
no	no	yes	no	yes	reactive	1	yes	4
no	no	no	no	yes	nonreactive	3	yes	2
no	no	no	yes	yes	nonreactive	1	yes	3
no	no	no	yes	yes	nonreactive	3	yes	4
no	no	no	no	yes	nonreactive	1	yes	4
yes	yes	yes	no	yes	nonreactive	2	yes	4
no	no	no	yes	yes	nonreactive	1	yes	4
no	yes	no	no	yes	nonreactive	2	yes	4
no	no	no	no	yes	nonreactive	2	yes	4
no	no	no	no	yes	nonreactive	3	yes	4
no	yes	yes	no	yes	nonreactive	2	yes	4
no	no	yes	yes	yes	nonreactive	2	yes	2
no	yes	yes	no	yes	nonreactive	3	yes	4
no	yes	no	no	yes	nonreactive	2	yes	1
no	no	no	no	yes	nonreactive	2	yes	4
no	no	yes	no	yes	nonreactive	1	yes	3
no	no	no	no	yes	nonreactive	1	yes	3
no	no	no	no	yes	nonreactive	2	yes	3
no	no	no	no	yes	nonreactive	1	yes	3
no	no	no	no	yes	nonreactive	1	yes	3
no	yes	yes	yes	yes	nonreactive	4	yes	3
no	yes	yes	no	yes	nonreactive	3	yes	1
no	yes	yes	no	yes	nonreactive	5	yes	3
no	no	yes	no	yes	nonreactive	1	yes	5
no	no	yes	no	yes	nonreactive	1	yes	3
no	no	yes	yes	yes	nonreactive	2	yes	3
no	no	no	yes	no	reactive	1	yes	1
no	no	no	yes	yes	nonreactive	1	yes	3
yes	yes	yes	yes	yes	nonreactive	1	yes	4
no	no	no	no	yes	nonreactive	1	yes	4
no	yes	yes	yes	yes	nonreactive	2	yes	3
no	no	yes	yes	yes	nonreactive	1	yes	2
no	no	no	yes	yes	nonreactive	1	yes	4
no	no	no	no	yes	nonreactive	2	yes	3
no	no	yes	no	no		3	yes	4
no	no	no	yes	yes	nonreactive	1	yes	4
no	no	no	yes	yes	nonreactive	6	yes	4
no	yes	yes	no	yes	nonreactive	2	yes	3

no	no	yes	yes	no	reactive	4	yes	4
no	yes	no	no	yes	nonreactive	2	yes	4
no	yes	yes	yes	yes	nonreactive	3	yes	4
no	yes	yes	no	yes	nonreactive	2	yes	4
no	yes	yes	yes	yes	nonreactive	2	yes	3
no	no	no	no	yes	nonreactive	2	yes	5
no	no	yes	no	yes	nonreactive	1	yes	4
no	yes	yes	yes	yes	nonreactive	5	yes	2
no	yes	yes	yes	yes	nonreactive	2	yes	4
no	no	no	yes	yes	nonreactive	10	yes	3
no	no	no	no	yes	nonreactive	2	yes	4
no	no	no	yes	yes	nonreactive	2	yes	4
no	yes	yes	yes	yes	nonreactive	4	yes	4
no	no	no	no	yes	nonreactive	2	yes	2
no	no	yes	yes	yes	nonreactive	1	yes	4
no	no	no	yes	yes	nonreactive	1	yes	2
no	no	no	yes	yes	nonreactive	3	yes	5
no	yes	no	yes	yes	nonreactive	3	yes	3
no	yes	no	yes	yes	nonreactive	1	yes	5
no	no	no	yes	yes	nonreactive	2	yes	2
no	yes	no	yes	yes	nonreactive	3	yes	3
no	yes	no	yes	yes	nonreactive	4	yes	2
	no	no	yes	yes	nonreactive	1	no	
no	yes	yes	no	yes	nonreactive	2	yes	3
no	no	yes	no	yes	nonreactive	3	yes	3
no	no	yes	yes	yes	nonreactive	2	yes	3
no	no	yes	no	yes	nonreactive	4	yes	2
yes	no	no	no	yes	nonreactive	6	yes	4
	no	no	yes	no		4	no	
yes	no	no	yes	yes	nonreactive	1	yes	4
no	no	yes	yes	yes	nonreactive	1	yes	3
no	yes	yes	yes	yes	nonreactive	4	yes	3
yes	no	no	yes	yes	nonreactive	4	yes	3
no	no	no	no	yes	nonreactive	1	yes	3
no	yes	no	yes	yes	reactive	3	yes	3
no	no	no	yes	yes	nonreactive	1	yes	4
	no	no	yes	yes	nonreactive	1	no	
no	no	no	yes	yes	nonreactive	5	yes	3
no	no	no	yes	yes	nonreactive	3	yes	3
no	no	no	yes	yes	nonreactive	2	yes	4
no	no	no	yes	yes	nonreactive	2	yes	4
no	no	yes	yes	yes	nonreactive	1	yes	3
yes	no	yes	yes	yes	nonreactive	1	yes	2
no	no	no	yes	yes	nonreactive	1	yes	3
no	no	yes	yes	yes	nonreactive	1	yes	4
no	no	no	yes	yes	nonreactive	4	yes	4
yes	no	yes	yes	yes	nonreactive	1	yes	3
no	yes	no	yes	yes	nonreactive	2	yes	4
no	yes	yes	no	yes	reactive	3	yes	4
no	no	yes	yes	yes	nonreactive	2	yes	2
no	yes	yes	no	yes	nonreactive	3	yes	4
no	no	yes	no	yes	nonreactive	1	yes	4

no	yes	no	yes	yes	nonreactive	2	yes	4
yes	yes	no	yes	yes	nonreactive	2	yes	2
no	no	no	yes	yes	nonreactive	1	yes	4
no	no	yes	no	yes	nonreactive	3	yes	3
no	no	no	no	yes	nonreactive	3	yes	3
no	yes	yes	no	yes	reactive	2	yes	5
no	yes	no	no	yes	nonreactive	2	yes	4
no	no	no	no	yes	nonreactive	2	yes	4
no	no	yes	no	yes	nonreactive	1	yes	2
no	no	yes	no	yes	nonreactive	2	yes	4
no	yes	no	no	yes	nonreactive	3	yes	4
no	no	no	no	yes	nonreactive	1	yes	4
no	yes	no	no	yes	nonreactive	3	yes	4
no	no	yes	no	yes	nonreactive	4	yes	4
no	no	no	no	yes	nonreactive	1	yes	4
no	no	yes	yes	yes	nonreactive	5	yes	4
no	no	yes	no	yes	nonreactive	3	yes	4
no	yes	yes	yes	yes	nonreactive	2	yes	4
no	no	no	yes	yes	nonreactive	1	yes	4
no	no	yes	no	yes	nonreactive	2	yes	4
no	no	no	yes	yes	nonreactive	3	yes	5
no	no	no	yes	yes	nonreactive	1	yes	4
no	no	no	yes	yes	nonreactive	4	yes	3
no	no	no	yes	yes	nonreactive	1	yes	4
no	no	no	yes	yes	nonreactive	3	yes	3
no	no	no	yes	yes	nonreactive	1	yes	4
no	no	yes	yes	yes	reactive	1	yes	5
no	no	no	yes	yes	nonreactive	1	yes	5
yes	no	no	yes	yes	nonreactive	1	yes	6
no	no	no	no	yes	nonreactive	2	yes	3
no	no	no	no	yes	nonreactive	1	yes	4
no	no	yes	no	yes	nonreactive	1	yes	4
no	no	yes	yes	yes	nonreactive	1	yes	4
no	no	yes	no	yes	nonreactive	1	yes	4
no	no	no	no	yes	nonreactive	1	yes	3
no	no	yes	no	yes	nonreactive	1	yes	3
yes	no	no	no	yes	nonreactive	1	yes	3
no	no	no	no	yes	nonreactive	1	yes	3
no	yes	yes	no	yes	nonreactive	1	yes	4
no	no	no	no	yes	nonreactive	1	yes	2
no	yes	no	no	yes	nonreactive	1	yes	3
yes	no	no	no	yes	nonreactive	2	yes	2
no	no	no	no	yes	nonreactive	3	yes	3
no	yes	yes	no	yes	nonreactive	2	yes	4
	no	no	no	yes	nonreactive	1	no	
no	yes	no	no	yes	nonreactive	4	yes	4
no	no	no	no	yes	nonreactive	5	yes	3
no	yes	no	yes	yes	nonreactive	1	yes	5
no	no	yes	no	yes	nonreactive	1	yes	4
no	no	no	no	yes	nonreactive	2	yes	4
no	no	yes	no	yes	nonreactive	1	yes	3
no	no	yes	no	yes	reactive	3	yes	3

no	yes	yes	yes	yes	nonreactive	3	yes		5
no	no	yes	no	yes	nonreactive	1	yes		5
no	no	yes	no	yes	nonreactive	1	yes		3
no	yes	no	no	yes	nonreactive	6	yes		4
	no	yes	yes	yes	nonreactive	1	no		
no	yes	no	no	yes	nonreactive	4	yes		5
no	no	no	no	yes	nonreactive	7	yes		4
no	no	no	no	yes	nonreactive	1	yes		4
no	yes	yes	no	yes	nonreactive	3	yes		4
no	no	yes	no	yes	nonreactive	3	yes		4
no	no	yes	no	yes	nonreactive	2	yes		3
no	no	no	yes	yes	nonreactive	4	yes		3
no	no	no	no	yes	nonreactive	1	yes		3
no	no	no	yes	yes	nonreactive	1	yes		1
no	no	yes	no	yes	nonreactive	5	yes		6
no	no	no	no	yes	nonreactive	1	yes		3
no	no	no	no	yes	nonreactive	2	yes		4
no	no	no	yes	yes	reactive	3	yes		3
	no	yes	yes	yes	nonreactive	5	no		
	no	yes	yes	yes	nonreactive	5	no		
no	no	no	yes	yes	nonreactive	1	yes		3
	no	yes	yes	yes	nonreactive	5	no		
no	no	no	no	yes	nonreactive	2	yes		2
no	no	no	yes	yes	nonreactive	2	yes		3
no	no	no	no	yes	nonreactive	2	yes		4
yes	no	yes	yes	yes	nonreactive	1	yes		4
yes	no	no	yes	yes	nonreactive	2	yes		3
no	yes	yes	yes	yes	nonreactive	3	yes		3
no	no	no	yes	yes	nonreactive	1	yes		3
yes	yes	yes	yes	yes	reactive	1	yes		4
no	no	no	yes	yes	nonreactive	4	yes		3
	no	no	no	no		2	no		
no	yes	no	yes	yes	nonreactive	2	yes		3
no	no	no	yes	yes	nonreactive	2	yes		3
no	no	no	no	yes	nonreactive	1	yes		4
no	no	no	no	yes	nonreactive	1	yes		2
no	no	no	yes	yes	nonreactive	1	yes		4
no	no	no	yes	yes	nonreactive	1	yes		3
	no	no	yes	yes	nonreactive	1	no		
no	no	no	yes	yes	reactive	1	yes		3
no	no	yes	yes	yes	nonreactive	3	yes		2
no	no	no	yes	yes	reactive	2	yes		2
no	yes	no	no	yes	nonreactive	2	yes		3
	no	no	yes	yes	nonreactive	2	no		
no	yes	yes	no	yes	nonreactive	1	yes		4
yes	no	no	yes	yes	nonreactive	1	yes		1
no	no	no	yes	yes	nonreactive	1	yes		3
no	no	no	no	yes	nonreactive	1	yes		4
no	no	no	no	yes	nonreactive	4	yes		2
no	yes	no	no	yes	nonreactive	3	yes		4
no	no	no	yes	yes	nonreactive	2	yes		3
yes	no	yes	yes	yes	nonreactive	3	yes		2

no	no	no	yes	yes	nonreactive	1	yes	2
no	no	no	yes	yes	nonreactive	1	yes	4
no	no	no	no	yes	nonreactive	1	yes	4
no	yes	yes	no	yes	nonreactive	4	yes	3
yes	no	no	yes	yes	nonreactive	5	yes	3
no	yes	yes	no	yes	nonreactive	2	yes	4
no	no	no	yes	yes	nonreactive	6	yes	3
no	no	no	yes	yes	nonreactive	1	yes	1
	no	no	yes	yes	nonreactive	1	no	
yes	no	no	yes	yes	nonreactive	6	yes	2
no	no	no	no	yes	nonreactive	3	yes	3
no	no	no	yes	yes	reactive	2	yes	5
no	no	yes	yes	yes	nonreactive	2	yes	4
yes	no	no	yes	yes	nonreactive	1	yes	3
no	no	no	yes	yes	nonreactive	1	yes	3
no	no	no	no	yes	nonreactive	4	yes	4
no	no	yes	yes	yes	nonreactive	7	yes	3
no	no	no	yes	yes	nonreactive	1	yes	2
yes	no	no	yes	yes	nonreactive	2	yes	2
no	no	no	no	yes	nonreactive	1	yes	5
no	no	no	yes	yes	nonreactive	1	yes	2
no	no	no	yes	yes	nonreactive	3	yes	5
no	no	no	yes	yes	nonreactive	1	yes	3
no	no	no	yes	yes	nonreactive	1	yes	2
no	yes	no	yes	yes	reactive	2	yes	6
no	no	yes	no	yes	nonreactive	1	yes	4
no	no	no	yes	yes	nonreactive	2	yes	3
no	no	no	yes	yes	nonreactive	2	yes	2
no	no	no	yes	yes	nonreactive	1	yes	3
no	yes	no	yes	yes	nonreactive	3	yes	3
yes	no	yes	no	yes	nonreactive	1	yes	3
no	no	no	no	yes	nonreactive	1	yes	4
no	no	no	no	yes	nonreactive	2	yes	4
no	yes	no	yes	yes	nonreactive	6	yes	2
no	no	no	yes	yes	nonreactive	4	yes	3
no	yes	no	no	yes	nonreactive	3	yes	3
no	no	no	yes	yes	nonreactive	1	yes	2
no	yes	no	yes	yes	nonreactive	3	yes	2
no	no	yes	no	yes	nonreactive	3	yes	4
no	yes	yes	no	yes	nonreactive	3	yes	3
no	no	yes	yes	yes	nonreactive	6	yes	3
yes	no	no	no	yes	nonreactive	2	yes	3
no	no	no	no	yes	nonreactive	1	yes	4
no	no	yes	no	yes	nonreactive	2	yes	2
no	no	yes	no	yes	nonreactive	1	yes	3
no	no	yes	yes	yes	nonreactive	1	yes	4
no	yes	no	no	yes	nonreactive	3	yes	3
no	no	yes	no	yes	nonreactive	3	yes	4
no	yes	yes	no	yes	nonreactive	1	yes	4
no	yes	yes	no	yes	nonreactive	4	yes	3
no	yes	no	no	yes	nonreactive	4	yes	3
no	no	no	no	yes	nonreactive	1	yes	2

no	no	no	no	yes	nonreactive	2	yes	3
no	no	yes	no	yes	nonreactive	2	yes	4
no	no	no	no	yes	nonreactive	3	yes	2
no	no	yes	no	yes	nonreactive	2	yes	3
no	no	yes	no	yes	nonreactive	3	yes	3
yes	no	yes	no	yes	nonreactive	2	yes	2
no	no	no	no	yes	nonreactive	1	yes	3
no	no	no	no	yes	nonreactive	1	yes	2
no	no	no	no	yes	nonreactive	2	yes	3
no	no	yes	no	yes	nonreactive	2	yes	2
no	no	no	no	yes	nonreactive	1	yes	3
yes	no	no	no	yes	nonreactive	1	yes	3
no	yes	yes	no	yes	nonreactive	2	yes	2
no	no	yes	no	yes	reactive	2	yes	3
no	no	no	no	yes	nonreactive	1	yes	2
no	no	yes	no	yes	nonreactive	2	yes	3
no	no	no	no	yes	nonreactive	1	yes	3
no	no	yes	no	yes	nonreactive	1	yes	3
no	no	yes	no	yes	nonreactive	1	yes	1
no	no	yes	no	yes	nonreactive	3	yes	2
no	no	yes	no	yes	nonreactive	1	yes	3
no	no	yes	no	yes	nonreactive	2	yes	3
no	yes	yes	no	yes	nonreactive	3	yes	3
no	no	yes	no	yes	nonreactive	1	yes	3
no	yes	yes	no	yes	nonreactive	3	yes	4
no	no	yes	no	yes	nonreactive	2	yes	4
yes	no	yes	no	yes	nonreactive	2	yes	2
yes	no	no	no	yes	nonreactive	2	yes	2
no	no	yes	no	yes	nonreactive	3	yes	2
yes	no	no	no	yes	reactive	1	yes	5
no	no	no	no	yes	nonreactive	1	yes	3
no	no	yes	no	yes	nonreactive	2	yes	3
no	no	yes	no	yes	nonreactive	2	yes	4
no	no	no	yes	yes	nonreactive	1	yes	2
no	no	no	yes	yes	nonreactive	1	yes	2
no	no	yes	no	yes	nonreactive	1	yes	3
no	no	yes	no	yes	nonreactive	1	yes	2
no	yes	yes	no	yes	nonreactive	4	yes	3
no	no	yes	no	yes	nonreactive	3	yes	2
no	yes	yes	no	yes	nonreactive	2	yes	2
no	no	no	no	yes	nonreactive	1	yes	3
no	yes	no	yes	yes	nonreactive	2	yes	2
no	no	yes	yes	yes	nonreactive	3	yes	2
yes	yes	yes	no	yes	nonreactive	3	yes	3
no	no	no	yes	yes	nonreactive	5	yes	2
no	yes	yes	no	yes	nonreactive	3	yes	3
no	no	no	yes	yes	nonreactive	2	yes	2
no	no	yes	no	yes	nonreactive	1	yes	3
no	no	no	yes	yes	nonreactive	1	yes	3
no	no	yes	yes	yes	nonreactive	4	yes	2
no	yes	no	no	yes	reactive	2	yes	4
no	no	no	yes	yes	nonreactive	2	yes	1

yes	no	yes	no	yes	nonreactive	1	yes	2
no	no	no	yes	yes	nonreactive	1	yes	2
no	no	no	yes	yes	nonreactive	1	yes	2
no	no	no	yes	yes	nonreactive	3	yes	3
no	no	no	yes	yes	nonreactive	2	yes	2
no	no	no	yes	yes	nonreactive	2	yes	1
no	yes	yes	no	yes	nonreactive	2	yes	5
no	no	yes	yes	yes	nonreactive	2	yes	3
no	yes	yes	yes	yes	reactive	2	yes	5
no	no	no	yes	yes	nonreactive	2	yes	2
no	no	no	yes	yes	nonreactive	2	yes	2
no	no	no	yes	yes	nonreactive	2	yes	2
no	yes	yes	yes	yes	nonreactive	3	yes	3
no	yes	yes	no	yes	nonreactive	3	yes	3
no	yes	no	yes	yes	nonreactive	3	yes	4
no	yes	yes	no	yes	nonreactive	4	yes	2
no	no	yes	no	yes	nonreactive	2	yes	3
no	no	yes	no	yes	nonreactive	1	yes	3
no	yes	yes	no	yes	nonreactive	2	yes	2
no	no	yes	no	yes	nonreactive	1	yes	2
no	no	yes	no	yes	nonreactive	2	yes	3
no	no	yes	no	yes	nonreactive	1	yes	3
yes	no	yes	no	yes	reactive	2	yes	4
no	no	no	yes	yes	nonreactive	1	yes	5
no	no	no	yes	yes	nonreactive	1	yes	4
yes	no	yes	yes	yes	nonreactive	1	yes	3

Q304	Q305	Q306	Q307	Q308	Q309	Q310	Q311	Q312
2	firststage	no		yes	others	alive	night	episiotomy
24	firststage	yes	highdegree	no		alive	day	episiotomy
4	secondstag	no		no		alive	night	SVD
12	firststage	no		no		alive	day	cs
2	firststage	no		yes	others	alive	night	instrumenta
3	secondstag	no		no		alive	day	SVD
3	secondstag	no		no		alive	day	SVD
2	secondstag	no		no		alive	day	SVD
1	firststage	no		no		alive	day	SVD
10	firststage	no		no		alive	day	episiotomy
24	firststage	yes	aph	no		alive	night	cs
8	secondstag	yes	others	no		alive	day	instrumenta
10	secondstag	no		no		alive	night	SVD
8	secondstag	no		no		alive	day	SVD
24	secondstag	no		no		alive	day	SVD
10	secondstag	no		yes	distress	alive	day	instrumenta
1	secondstag	no		no		alive	day	SVD
6	secondstag	no		no		alive	night	SVD
2	secondstag	yes	others	no		alive	night	cs
24	secondstag	yes	pph	no		alive	night	SVD
3	firststage	no		no		alive	day	SVD
24	secondstag	no		no		alive	night	cs
24	secondstag	yes	others	no		alive	night	cs
1	secondstag	no		no		alive	night	cs
15	secondstag	no		no		alive	day	SVD
12	secondstag	no		no		alive	day	SVD
12	secondstag	yes	pph	no		alive	day	SVD
24	secondstag	yes	pph	yes	physicalinju	died	night	SVD
24	secondstag	no		no		alive	night	SVD
4	firststage	yes	hypertensio	yes	others	died	night	instrumenta
8	secondstag	no		no		alive	day	SVD
8	secondstag	no		no		alive	day	SVD
24	firststage	no		no		alive	day	SVD
8	firststage	no		no		alive	day	SVD
10	firststage	no		no		alive	day	SVD
10	firststage	yes	others	no		alive	day	cs
24	firststage	yes	others	no		alive	day	cs
5	secondstag	no		no		alive	night	cs
10	firststage	no		no		alive	night	SVD
2	secondstag	yes	others	no		alive	day	cs
1	firststage	no		yes	distress	alive	day	episiotomy
1	firststage	no		no		alive	day	SVD
5	firststage	no		no		alive	day	episiotomy
8	firststage	no		no		alive	day	episiotomy
12	firststage	no		yes	distress	alive	day	cs
1	firststage	no		yes	others	alive	night	cs
1	firststage	yes	hypertensio	yes	physicalinju	died	night	instrumenta
8	firststage	no		no		alive	day	SVD
8	secondstag	yes	highdegree	no		alive	day	SVD
1	firststage	no		no		alive	night	SVD
5	firststage	no		no		alive	day	instrumenta

15	firststage	yes	highdegree	no		alive	day	episiotomy
2	firststage	yes	others	no		alive	day	cs
2	firststage	no		no		alive	day	cs
24	secondstag	yes	pph	yes	distress	alive	day	episiotomy
2	firststage	yes	others	no		alive	day	cs
1	firststage	no		no		alive	day	SVD
4	firststage	no		no		alive	night	SVD
5	firststage	no		yes	distress	alive	night	SVD
6	firststage	no		no		alive	night	SVD
3	firststage	no		no		alive	day	SVD
2	firststage	no		no		alive	night	SVD
48	secondstag	yes	hypertensio	no		alive	night	SVD
43	secondstag	no		no		alive	night	SVD
12	firststage	yes	highdegree	no		alive	day	episiotomy
2	firststage	yes	highdegree	no		alive	day	episiotomy
6	firststage	no		yes	physicalinju	died	day	instrumenta
8	firststage	yes	hypertensio	no		alive	night	cs
10	firststage	no		yes	others	died	day	instrumenta
4	firststage	yes	hypertensio	no		alive	day	SVD
24	secondstag	no		no		alive	day	SVD
15	secondstag	no		no		alive	day	episiotomy
2	firststage	no		no		alive	day	SVD
3	firststage	no		no		alive	day	cs
1	firststage	no		yes	others	alive	day	cs
2	firststage	no		no		alive	day	SVD
4	firststage	no		no		alive	day	episiotomy
1	firststage	no		no		alive	day	SVD
4	firststage	no		yes	malpresent	alive	day	SVD
3	firststage	no		no		alive	day	episiotomy
2	firststage	no		no		alive	day	episiotomy
7	firststage	no		no		alive	day	episiotomy
1	firststage	no		yes	distress	alive	night	cs
1	firststage	no		no		alive	night	episiotomy
6	firststage	no		no		alive	night	SVD
12	firststage	no		no		alive	day	SVD
1	firststage	no		no		alive	day	SVD
12	secondstag	no		no		alive	night	SVD
48	firststage	yes	highdegree	no		alive	night	episiotomy
1	firststage	no		no		alive	day	SVD
7	firststage	yes	others	no		alive	night	cs
4	firststage	yes	others	yes	physicalinju	alive	night	cs
15	secondstag	no		no		alive	day	SVD
5	firststage	no		no		alive	day	SVD
1	firststage	no		no		alive	day	episiotomy
6	firststage	no		yes	others	died	day	instrumenta
12	firststage	yes	pph	no		alive	night	cs
18	secondstag	no		no		alive	day	cs
1	firststage	no		no		alive	day	SVD
1	firststage	no		no		alive	day	SVD
1	firststage	no		no		died	day	SVD
	elective	yes	hypertensio	no		alive	day	cs
2	firststage	no		no		alive	night	SVD

8	secondstag	no		no		alive	night	SVD
6	secondstag	no		no		alive	day	SVD
9	secondstag	no		no		alive	day	SVD
2	firststage	no		no		alive	day	SVD
12	firststage	no		no		alive	night	SVD
72	secondstag	no		no		alive	day	instrumenta
11	firststage	no		yes	distress	alive	day	cs
2	firststage	no		no		alive	night	episiotomy
	elective	no		no		alive	day	cs
16	secondstag	yes	pph	yes	others	died	day	SVD
6	firststage	no		no		alive	day	SVD
13	secondstag	no		no		alive	day	instrumenta
	elective	no		yes	malpresent	alive	night	cs
0.5	firststage	no		yes	distress	alive	night	cs
10	firststage	no		yes	malpresent	alive	day	cs
	elective	yes	hypertensio	yes	physicalinju	died	day	SVD
	elective	yes	others	no		alive	day	cs
10	firststage	no		yes	malpresent	died	night	cs
24	firststage	no		yes	distress	alive	day	cs
16	firststage	no		yes	others	died	night	cs
	elective	no		yes	others	died	day	SVD
	elective	yes	hypertensio	yes	others	died	night	cs
3	firststage	yes	pph	yes	distress	died	night	SVD
0.5	firststage	no		no		alive	night	SVD
1	secondstag	no		no		alive	night	instrumenta
0.5	firststage	no		no		alive	day	SVD
0.7	firststage	no		no		alive	night	instrumenta
0.5	firststage	no		yes	others	died	day	SVD
1	firststage	no		no		alive	night	SVD
10	firststage	no		no		alive	night	SVD
10	secondstag	no		no		alive	day	SVD
3	firststage	yes	hypertensio	yes	physicalinju	died	day	instrumenta
24	firststage	yes	pph	no		alive	day	episiotomy
1	firststage	no		no		alive	day	SVD
	elective	no		yes	distress	died	night	cs
24	secondstag	yes	others	yes	others	died	night	cs
	elective	yes	pph	yes	others	died	night	episiotomy
15	secondstag	yes	pph	yes	malpresent	died	day	cs
16	secondstag	no		yes	physicalinju	died	day	instrumenta
15	secondstag	yes	pph	no		died	day	SVD
15	secondstag	no		yes	malpresent	died	day	cs
	elective	yes	hypertensio	yes	others	alive	night	cs
4	firststage	yes	hypertensio	no		alive	night	SVD
12	firststage	no		no		alive	night	cs
6	firststage	no		yes	others	alive	night	cs
3	firststage	no		no		alive	night	cs
24	firststage	yes	retainedpla	no		alive	day	episiotomy
12	secondstag	no		no		alive	day	episiotomy
8	firststage	no		no		alive	day	cs
6	secondstag	no		no		alive	day	cs
4	firststage	no		no		alive	day	SVD
11	secondstag	no		no		alive	day	SVD

	elective	yes	others	no		alive	night	cs
3	firststage	yes	others	no		alive	day	cs
	elective	yes	others	yes	distress	alive	night	cs
2	firststage	no		no		alive	day	episiotomy
5	firststage	no		yes	distress	alive	night	SVD
1	firststage	no		no		alive	night	SVD
3	firststage	no		no		alive	day	SVD
0.5	firststage	no		yes	distress	alive	night	episiotomy
24	firststage	no		no		alive	night	episiotomy
0.5	firststage	no		no		alive	day	SVD
2	secondstag	no		no		alive	night	episiotomy
1	firststage	no		no		alive	day	episiotomy
1	firststage	no		no		alive	night	SVD
3	firststage	no		no		alive	day	SVD
2	firststage	no		no		alive	day	episiotomy
	firststage	no		no		alive	day	SVD
10	firststage	no		no		alive	day	episiotomy
	elective	yes	others	no		alive	day	cs
16	secondstag	yes	others	no		alive	day	cs
	elective	no		no		alive	night	cs
4	firststage	no		yes	malpresent	alive	night	cs
3.5	firststage	yes	others	yes	distress	alive	night	cs
18	secondstag	no		no		alive	day	SVD
12	secondstag	yes	others	no		alive	night	cs
6	firststage	no		no		alive	day	SVD
	elective	yes	hypertensio	no		died	night	SVD
	elective	no		yes	malpresent	alive	night	cs
3	firststage	yes	hypertensio	no		alive	night	instrumenta
	elective	yes	hypertensio	no		alive	day	cs
2	firststage	no		no		alive	night	instrumenta
2	firststage	no		no		alive	day	instrumenta
13	secondstag	no		yes	distress	alive	day	SVD
1	firststage	no		no		alive	night	SVD
5	secondstag	no		no		alive	day	SVD
2	firststage	no		yes	distress	alive	day	instrumenta
1	secondstag	no		no		alive	day	SVD
1	firststage	no		no		alive	day	SVD
1	firststage	no		no		alive	day	SVD
0.5	firststage	no		no		alive	night	SVD
2	firststage	yes	pph	no		alive	night	SVD
24	firststage	no		no		alive	day	episiotomy
2	firststage	no		no		alive	night	episiotomy
2	firststage	no		yes	distress	died	day	SVD
3	firststage	no		no		alive	day	SVD
9	firststage	no		no		alive	day	SVD
8	firststage	no		no		alive	night	SVD
1	firststage	no		yes	others	alive	day	SVD
7	secondstag	no		no		alive	day	SVD
1.5	firststage	no		no		alive	night	cs
4	firststage	no		no		alive	day	cs
2	firststage	no		no		alive	night	cs
2	firststage	no		no		alive	day	cs

2	firststage	no		no		alive	day	SVD
8	secondstag	no		no		alive	night	SVD
10	firststage	no		no		alive	day	SVD
3	firststage	no		no		alive	night	SVD
3	firststage	no		no		alive	night	SVD
8	secondstag	no		no		alive	day	SVD
1	firststage	no		no		alive	night	SVD
3	firststage	no		no		alive	night	episiotomy
3	secondstag	no		no		alive	day	SVD
5	secondstag	no		no		alive	day	SVD
6	firststage	no		no		alive	night	SVD
18	secondstag	yes	others	yes	distress	died	night	instrumenta
2	firststage	no		no		alive	day	SVD
2.5	firststage	no		no		alive	day	SVD
4	firststage	no		no		alive	day	SVD
3	firststage	no		no		alive	day	SVD
10	firststage	yes	pph	no		alive	day	SVD
15	secondstag	no		no		alive	night	episiotomy
11	firststage	no		no		alive	day	SVD
13	firststage	no		no		alive	day	SVD
1.5	firststage	no		yes	malpresent	alive	day	cs
1	firststage	no		no		alive	day	SVD
2	firststage	no		no		alive	night	episiotomy
9	firststage	no		no		alive	night	SVD
1	firststage	no		no		alive	day	SVD
7	firststage	no		yes	distress	alive	day	SVD
4	firststage	yes	hypertensio	no		alive	day	SVD
8	secondstag	no		no		alive	day	SVD
14	secondstag	no		yes	distress	alive	night	cs
10	secondstag	yes	retainedpla	no		alive	night	SVD
13	secondstag	no		no		alive	night	SVD
10	secondstag	no		no		alive	day	SVD
1	firststage	no		no		alive	day	SVD
15	secondstag	yes	aph	yes	malpresent	alive	night	episiotomy
2	firststage	no		no		alive	day	SVD
3	firststage	no		no		alive	day	episiotomy
5	firststage	yes	others	no		alive	day	instrumenta
10	firststage	no		no		alive	day	SVD
3	firststage	yes	hypertensio	no		alive	night	cs
10	firststage	yes	others	yes	malpresent	alive	night	cs
10	secondstag	yes	retainedpla	yes	distress	died	night	instrumenta
4	firststage	yes	others	no		alive	night	cs
2	firststage	no		no		alive	day	SVD
12	firststage	yes	aph	no		alive	night	cs
1	firststage	no		no		alive	day	SVD
10	firststage	no		no		alive	day	SVD
24	firststage	yes	others	yes	distress	alive	night	cs
1	firststage	no		no		alive	day	SVD
3	firststage	no		no		alive	night	SVD
2	firststage	no		no		alive	day	SVD
2	firststage	no		yes	malpresent	alive	night	cs
24	secondstag	yes	others	no		alive	night	episiotomy

15	secondstag	yes	others	no		alive	night	episiotomy
10	firststage	no		no		alive	day	SVD
5	firststage	no		no		alive	night	episiotomy
5	firststage	no		no		died	night	instrumenta
10	secondstag	yes	hypertensio	no		alive	day	cs
1	firststage	no		no		alive	day	SVD
26	secondstag	yes	hypertensio	no		alive	day	SVD
9	firststage	yes	pph	no		died	night	SVD
10	firststage	no		yes	malpresent	alive	night	cs
24	firststage	yes	others	yes	others	alive	night	cs
2	firststage	no		yes	distress	alive	day	episiotomy
3	firststage	no		yes	others	alive	night	SVD
13	secondstag	yes	others	no		alive	night	cs
11	firststage	no		no		died	night	episiotomy
5	firststage	no		yes	malpresent	alive	night	cs
1	firststage	no		no		alive	day	SVD
16	secondstag	yes	others	no		alive	night	cs
15	firststage	yes	others	yes	distress	alive	night	cs
20	secondstag	yes	hypertensio	no		alive	night	cs
2	firststage	no		no		alive	night	episiotomy
11	firststage	no		yes	distress	alive	day	instrumenta
5	firststage	yes	others	yes	malpresent	alive	day	cs
10	firststage	yes	others	no		alive	night	episiotomy
13	firststage	yes	others	no		alive	night	instrumenta
	elective	no		no		alive	night	cs
2	firststage	no		no		alive	day	SVD
24	secondstag	yes	others	no		alive	day	instrumenta
12	firststage	no		no		alive	night	instrumenta
7	firststage	yes	aph	no		alive	night	SVD
10	firststage	no		yes	distress	alive	day	cs
3	firststage	no		no		alive	day	SVD
4	firststage	no		no		alive	day	SVD
1	firststage	no		no		alive	night	SVD
15	firststage	yes	others	no		alive	night	cs
12	firststage	no		yes	distress	alive	day	cs
4	firststage	no		no		alive	day	SVD
10	firststage	yes	others	no		alive	night	instrumenta
2	firststage	yes	others	no		alive	day	SVD
1	firststage	no		no		alive	day	SVD
2	firststage	no		no		alive	day	SVD
17	secondstag	yes	pph	no		alive	night	cs
2	firststage	no		no		alive	night	episiotomy
5	firststage	no		no		alive	day	SVD
8	firststage	no		no		died	day	SVD
0.5	firststage	no		no		alive	day	SVD
1	firststage	no		no		alive	day	episiotomy
0.5	firststage	no		no		alive	day	SVD
4	firststage	no		no		alive	day	SVD
2	secondstag	no		no		alive	day	SVD
1	firststage	no		no		alive	day	SVD
2	firststage	no		no		alive	day	SVD
0.5	firststage	no		no		alive	night	SVD

3	firststage	no		no		alive	day	SVD
4	firststage	no		no		alive	day	SVD
4	firststage	no		no		alive	night	SVD
5	firststage	no		no		alive	night	SVD
2	firststage	no		no		alive	day	SVD
4	firststage	no		yes	distress	alive	day	SVD
6	firststage	no		no		alive	night	episiotomy
3	firststage	no		no		alive	day	SVD
4	firststage	no		no		alive	day	SVD
5	firststage	no		yes	others	died	day	SVD
1	firststage	no		no		alive	night	SVD
1	firststage	no		no		alive	day	SVD
2	firststage	no		no		alive	day	SVD
5	firststage	no		no		alive	day	SVD
2	firststage	no		no		alive	day	SVD
2	firststage	no		no		alive	night	episiotomy
2	firststage	no		no		alive	day	SVD
1	firststage	no		no		alive	day	SVD
2	firststage	no		no		alive	night	episiotomy
2	firststage	no		no		alive	day	SVD
0.5	firststage	no		no		alive	day	SVD
0.5	firststage	yes	others	no		alive	day	SVD
0.5	firststage	no		no		alive	day	SVD
0.5	firststage	no		no		alive	day	episiotomy
5	firststage	no		no		alive	night	SVD
6	firststage	no		no		alive	day	SVD
1	firststage	no		no		alive	night	SVD
6	firststage	no		no		alive	day	SVD
5	firststage	no		no		alive	night	SVD
2	firststage	no		no		alive	night	SVD
2	firststage	no		no		alive	day	SVD
1	firststage	no		yes	distress	alive	night	SVD
1	firststage	no		no		alive	day	SVD
4	firststage	yes	others	no		alive	night	instrumenta
4	firststage	yes	hypertensio	no		alive	night	cs
2	firststage	no		no		alive	day	SVD
2	firststage	no		no		alive	day	SVD
3	firststage	no		no		alive	day	SVD
2	firststage	no		no		alive	day	episiotomy
4	firststage	no		no		alive	day	SVD
0.5	firststage	no		yes	others	alive	night	episiotomy
8	firststage	yes	others	no		alive	night	instrumenta
6	firststage	yes	others	yes	distress	alive	night	cs
2	firststage	no		yes	distress	alive	night	SVD
3	firststage	yes	others	yes	others	alive	night	cs
4	firststage	yes	hypertensio	no		alive	night	cs
8	secondstag	yes	others	no		alive	night	instrumenta
1	firststage	no		no		alive	day	SVD
1	firststage	no		no		alive	day	SVD
10	secondstag	yes	others	no		alive	night	instrumenta
1	firststage	no		no		alive	night	SVD
5	firststage	no		yes	malpresent	died	night	instrumenta

2	firststage	yes	pph	no		alive	night	SVD
2	firststage	yes	others	yes	others	alive	day	SVD
12	secondstag	yes	others	no		alive	night	episiotomy
24	secondstag	yes	others	yes	distress	died	night	instrumenta
6	firststage	yes	others	no		alive	night	SVD
10	secondstag	yes	aph	yes	others	died	night	instrumenta
1	firststage	no		no		alive	night	SVD
2	firststage	yes	hypertensio	no		alive	night	cs
2	firststage	no		no		alive	night	SVD
3	firststage	yes	hypertensio	no		alive	night	instrumenta
4	firststage	yes	others	no		died	night	instrumenta
15	secondstag	yes	others	no		alive	night	cs
0.5	firststage	no		no		alive	night	SVD
0.5	firststage	no		no		alive	night	SVD
14	firststage	no		no		alive	night	cs
2	firststage	no		no		alive	day	SVD
2	firststage	no		no		alive	day	SVD
1	firststage	no		no		alive	day	SVD
3	firststage	no		no		alive	day	SVD
2	firststage	no		no		alive	day	SVD
1	firststage	no		no		alive	day	SVD
0.5	firststage	no		no		alive	day	SVD
2	firststage	no		no		alive	day	SVD
6	firststage	yes	hypertensio	no		alive	night	episiotomy
2	firststage	no		no		alive	night	SVD
1	firststage	no		no		alive	night	episiotomy

Q313	Q401	Q402	Q403	Q404	Q405	Q406	Q501	Q502
2	hospital	male	midwife	6	8	33	no	yes
2	hospital	male	midwife	4	8	28	no	yes
1	hospital	male	midwife	4	8	30	yes	yes
4	hospital	male	midwife	2	24	30	no	yes
1	hospital	male	midwife	5	8	30	no	yes
1	hospital	male	midwife	3	8	28	no	yes
1	hospital	male	midwife	4	8	29	no	yes
1	hospital	female	midwife	3	8	28	no	yes
1	hospital	male	doctor	6	24	38	yes	yes
1	hospital	male	midwife	4	8	28	no	yes
2	hospital	male	midwife	3	8	29	no	yes
1	hospital	male	doctor	3	24	29	no	yes
1	hospital	male	midwife	3	8	28	no	yes
1	hospital	male	doctor	1	8	30	no	yes
1	hospital	male	midwife	3	24	30	no	yes
1	hospital	male	midwife	5	8	30	yes	yes
1	hospital	male	doctor	3	8	28	no	yes
1	hospital	female	midwife	3	8	27	no	yes
2	hospital	male	doctor	3	24	30	yes	yes
3	healthcente	female	midwife	4	8	26	no	yes
1	hospital	male	midwife	2	8	30	no	yes
6	hospital	male	doctor	3	24	27	no	yes
2	hospital	female	doctor	3	24	28	no	yes
2	hospital	male	doctor	2	24	30	no	yes
1	hospital	female	midwife	3	8	26	no	yes
1	hospital	male	midwife	4	8	29	no	yes
1	healthcente	female	midwife	2	8	27	yes	no
1	hospital	male	doctor	6	12	30	yes	yes
2	hospital	male	midwife	3	8	28	no	yes
2	hospital	female	midwife	3	8	27	no	yes
1	hospital	male	midwife	4	8	31	yes	yes
1	hospital	female	midwife	5	8	31	yes	yes
2	hospital	male	midwife	1	8	30	no	yes
2	hospital	male	midwife	3	8	28	no	yes
2	hospital	male	midwife	4	8	28	no	yes
2	hospital	male	doctor	2	24	28	no	yes
2	hospital	male	doctor	3	8	30	no	yes
1	hospital	male	doctor	2	24	28	no	yes
1	hospital	male	midwife	3	8	28	no	yes
4	hospital	male	doctor	2	24	29	no	yes
3	hospital	male	midwife	1	24	29	no	yes
1	hospital	female	midwife	5	8	27	yes	yes
1	hospital	male	doctor	2	8	27	no	yes
3	hospital	male	doctor	1	24	28	no	no
3	hospital	male	doctor	2	8	32	no	yes
5	hospital	male	doctor	3	24	32	no	yes
2	hospital	male	midwife	3	8	30	no	no
1	hospital	female	midwife	12	8	45	no	no
1	hospital	female	midwife	6	8	30	no	yes
3	hospital	male	doctor	1	24	28	no	yes
2	healthcente	male	others	4	24	36	no	yes

1	hospital	male	midwife	6	24	32	no	yes
4	hospital	male	midwife	6	24	33	no	yes
3	hospital	male	doctor	12	24	45	yes	yes
2	hospital	male	midwife	4	8	29	no	yes
1	hospital	male	doctor	6	8	35	no	yes
1	healthcente	female	midwife	6	15	33	no	yes
1	healthcente	male	midwife	1	17	37	yes	yes
1	healthcente	male	midwife	2	17	39	yes	yes
1	healthcente	male	midwife	4	17	37	yes	yes
1	healthcente	female	midwife	2	17	24	yes	yes
1	healthcente	female	midwife	2	17	24	yes	yes
1	healthcente	male	midwife	4	17	37	yes	yes
1	healthcente	female	midwife	10	12	30	no	yes
1	hospital	male	midwife	4	8	32	no	yes
2	hospital	male	midwife	3	16	28	no	yes
2	hospital	male	doctor	3	24	32	no	yes
2	hospital	male	doctor	3	12	32	no	no
3	hospital	male	midwife	3	8	30	no	yes
3	hospital	male	doctor	2	24	30	no	yes
2	hospital	male	midwife	3	8	33	yes	yes
1	hospital	female	midwife	3	8	36	no	yes
1	hospital	female	doctor	8	8	33	no	yes
1	hospital	male	doctor	5	24	36	no	yes
1	hospital	male	doctor	7	24	38	no	yes
1	hospital	female	midwife	4	8	28	no	yes
1	hospital	female	midwife	4	8	28	no	yes
1	hospital	female	midwife	8	12	35	yes	yes
1	hospital	female	midwife	8	8	35	no	yes
1	hospital	female	midwife	2	8	24	no	yes
3	hospital	female	midwife	2	8	24	no	yes
1	hospital	female	doctor	5	8	29	yes	yes
5	hospital	male	doctor	7	18	36	no	yes
1	hospital	female	midwife	8	8	28	no	yes
3	hospital	female	midwife	7	8	28	no	yes
2	hospital	female	midwife	2	8	24	no	yes
2	hospital	female	midwife	5	8	32	no	yes
2	hospital	female	midwife	2	8	24	no	yes
9	hospital	female	doctor	8	24	35	no	yes
2	hospital	male	midwife	3	8	26	no	yes
2	hospital	male	midwife	2	8	27	no	yes
3	hospital	male	midwife	3	8	29	no	yes
2	hospital	male	doctor	3	8	33	no	yes
1	hospital	male	others	3	8	26	yes	yes
1	hospital	female	doctor	4	8	30	no	yes
1	hospital	male	doctor	11	24	38	no	yes
3	hospital	male	doctor	3	8	30	no	yes
2	hospital	female	doctor	2	24	29	no	yes
1	hospital	male	midwife	3	8	28	yes	yes
1	hospital	male	midwife	5	8	35	no	yes
1	hospital	male	midwife	1	8	26	no	yes
5	hospital	male	doctor	3	24	30	no	yes
1	hospital	male	midwife	2	24	27	no	yes

1	hospital	female	midwife	3	8	28	no	yes
1	hospital	male	midwife	5	8	32	no	yes
1	hospital	male	midwife	3	8	28	no	yes
1	hospital	female	midwife	6	8	35	no	yes
2	hospital	male	midwife	3	8	28	no	yes
3	hospital	male	midwife	5	8	28	no	yes
3	hospital	male	doctor	8	24	39	yes	yes
9	hospital	male	midwife	4	8	29	no	yes
3	hospital	male	doctor	7	24	36	no	yes
2	hospital	male	midwife	5	8	29	no	no
1	hospital	female	midwife	4	8	28	no	yes
1	hospital	female	doctor	4	8	28	no	yes
45	hospital	male	doctor	10	24	45	yes	yes
3	hospital	male	doctor	4	24	32	no	yes
3	hospital	male	doctor	10	24	45	no	yes
20	hospital	male	midwife	4	8	28	no	yes
8	hospital	male	doctor	4	24	32	no	yes
5	hospital	male	midwife	10	24	45	no	yes
3	hospital	male	midwife	3	24	29	no	yes
5	hospital	female	doctor	3	24	31	no	yes
4	hospital	male	doctor	4	24	32	yes	yes
6	hospital	male	doctor	4	24	33	no	yes
6	hospital	male	midwife	4	8	28	no	yes
1	hospital	male	doctor	8	8	32	no	yes
2	hospital	female	doctor	8	12	35	no	yes
1	hospital	female	midwife	4	8	32	yes	yes
2	hospital	male	doctor	10	18	39	no	yes
1	hospital	male	midwife	7	8	35	no	yes
1	hospital	male	doctor	1	12	35	no	yes
1	hospital	male	midwife	7	8	29	no	yes
1	hospital	female	midwife	4	8	29	no	yes
2	hospital	male	midwife	5	12	29	no	yes
3	hospital	female	midwife	3	12	24	no	yes
1	hospital	male	doctor	2	8	30	no	yes
4	hospital	male	midwife	6	24	35	no	yes
3	hospital	male	doctor	5	24	35	no	yes
3	hospital	male	midwife	6	24	35	no	yes
5	hospital	male	doctor	4	24	29	no	yes
2	hospital	male	doctor	4	24	35	no	yes
1	hospital	male	midwife	4	8	28	no	yes
3	hospital	female	doctor	3	24	28	no	yes
5	hospital	male	doctor	4	24	29	no	yes
2	hospital	female	midwife	5	8	32	no	yes
2	hospital	female	doctor	2	24	30	no	yes
2	hospital	male	doctor	3	24	28	no	yes
2	hospital	male	doctor	2	24	29	no	yes
1	hospital	female	midwife	7	8	28	yes	yes
2	hospital	female	doctor	3	12	28	yes	yes
3	hospital	male	doctor	5	12	35	yes	yes
2	hospital	male	doctor	6	8	35	yes	yes
2	hospital	male	midwife	5	8	29	yes	yes
2	hospital	male	midwife	5	8	30	no	yes

4	hospital	male	midwife	3	24	29	yes	yes
4	hospital	male	midwife	6	24	35	no	yes
3	hospital	male	doctor	6	24	32	no	yes
2	healthcente	male	midwife	6	15	28	yes	yes
1	healthcente	female	midwife	3	15	27	yes	yes
2	healthcente	female	midwife	5	15	29	no	yes
2	healthcente	female	midwife	5	15	29	yes	yes
1	healthcente	female	midwife	4	18	28	no	yes
1	healthcente	male	midwife	7	8	28	yes	yes
1	healthcente	male	midwife	7	8	28	no	yes
1	healthcente	female	midwife	4	12	27	yes	yes
1	hospital	female	midwife	10	8	32	no	yes
1	healthcente	male	midwife	7	8	28	yes	yes
1	healthcente	male	midwife	7	8	28	yes	yes
1	hospital	male	midwife	5	8	25	no	yes
3	hospital	male	midwife	4	8	28	no	yes
1	hospital	male	midwife	4	8	28	no	yes
4	hospital	male	doctor	4	24	30	no	yes
4	hospital	female	doctor	3	24	28	no	yes
3	hospital	female	doctor	3	24	29	no	yes
3	hospital	male	doctor	2	24	34	no	yes
3	hospital	female	doctor	3	24	28	no	yes
2	hospital	male	midwife	5	12	28	no	yes
5	hospital	male	doctor	3	24	32	no	yes
2	hospital	male	doctor	2	24	35	no	yes
3	hospital	male	doctor	2	12	35	no	yes
3	hospital	female	doctor	3	24	29	no	yes
6	hospital	male	doctor	3	24	31	no	yes
11	hospital	female	doctor	3	24	29	no	yes
1	hospital	male	midwife	4	8	28	yes	yes
1	hospital	male	midwife	3	8	26	yes	yes
10	hospital	male	midwife	8	8	30	no	yes
1	hospital	female	midwife	6	8	28	no	yes
1	hospital	female	midwife	6	8	28	yes	yes
2	hospital	male	healthoffice	6	24	37	no	yes
1	healthcente	female	midwife	6	15	30	no	yes
1	healthcente	female	midwife	6	15	30	no	yes
1	healthcente	female	midwife	8	15	32	no	yes
1	healthcente	female	midwife	8	15	32	no	yes
1	healthcente	female	midwife	6	15	30	yes	yes
1	healthcente	female	midwife	8	15	32	yes	yes
1	healthcente	female	midwife	6	8	28	no	yes
2	healthcente	male	midwife	3	8	28	no	yes
2	healthcente	female	midwife	3	15	28	yes	yes
1	hospital	female	midwife	5	8	26	yes	yes
1	hospital	female	midwife	6	15	28	yes	yes
1	hospital	male	midwife	5	10	26	no	yes
1	hospital	female	midwife	8	24	26	no	yes
5	hospital	male	others	6	24	32	no	yes
5	hospital	male	others	6	24	32	yes	yes
4	hospital	male	others	4	24	32	yes	yes
9	hospital	male	others	6	24	32	yes	yes

1	hospital	male	midwife	8	17	27	no	yes
1	hospital	male	midwife	3	8	28	no	yes
1	hospital	female	midwife	5	8	25	no	yes
1	hospital	female	midwife	5	8	25	no	yes
1	hospital	male	midwife	5	8	25	no	no
1	hospital	female	midwife	4	8	28	yes	yes
9	hospital	female	midwife	4	8	28	yes	yes
1	hospital	male	midwife	3	8	26	yes	yes
1	hospital	male	midwife	3	8	26	no	yes
1	hospital	female	midwife	3	8	26	yes	yes
1	hospital	male	midwife	4	14	29	no	yes
2	hospital	male	midwife	4	24	30	no	yes
1	hospital	female	midwife	5	8	28	yes	yes
1	hospital	female	midwife	4	8	29	no	yes
1	hospital	female	midwife	3	8	27	no	yes
1	hospital	male	midwife	3	8	28	no	yes
1	hospital	male	midwife	6	8	32	no	yes
7	hospital	male	midwife	3	24	29	no	yes
1	hospital	female	midwife	5	8	28	yes	yes
2	hospital	female	midwife	3	8	27	yes	yes
3	hospital	female	doctor	3	8	29	no	yes
3	hospital	female	midwife	3	8	27	yes	yes
4	hospital	female	midwife	3	8	27	yes	yes
8	hospital	male	midwife	4	8	28	yes	yes
1	hospital	male	midwife	6	8	32	no	yes
1	hospital	male	midwife	4	8	28	no	yes
12	hospital	female	midwife	3	8	29	no	no
2	hospital	male	midwife	3	8	30	no	no
4	hospital	female	doctor	6	12	34	yes	yes
4	hospital	female	midwife	4	8	27	yes	yes
3	hospital	male	midwife	4	8	27	no	yes
2	hospital	male	midwife	3	8	28	yes	no
1	hospital	male	midwife	2	8	27	no	yes
2	hospital	male	doctor	6	24	32	no	yes
1	hospital	female	midwife	2	8	25	no	yes
2	hospital	male	midwife	2	8	25	no	yes
1	hospital	male	midwife	2	8	25	no	yes
2	hospital	female	midwife	2	8	25	no	yes
3	hospital	female	doctor	2	24	25	no	yes
4	hospital	male	doctor	6	24	31	no	yes
2	hospital	male	doctor	2	12	29	no	yes
4	hospital	male	doctor	3	24	29	no	yes
1	hospital	female	midwife	5	8	28	no	yes
4	hospital	male	doctor	2	24	29	no	yes
1	hospital	male	midwife	3	8	28	no	yes
2	hospital	female	midwife	5	8	28	no	yes
7	hospital	female	doctor	3	24	29	no	yes
1	hospital	female	midwife	5	8	28	no	yes
1	hospital	male	doctor	2	24	28	no	yes
1	hospital	female	midwife	2	8	25	yes	yes
6	hospital	male	doctor	4	24	32	no	yes
2	hospital	male	doctor	4	24	32	no	yes

2	hospital	male	doctor	3	24	30	no	yes
1	hospital	female	midwife	2	8	25	no	yes
2	hospital	female	midwife	5	8	29	no	yes
2	hospital	male	doctor	4	24	32	no	yes
8	hospital	male	doctor	4	24	32	no	yes
1	hospital	female	midwife	5	8	28	yes	yes
5	hospital	male	doctor	8	24	36	no	yes
2	hospital	female	midwife	5	8	28	yes	yes
7	hospital	male	doctor	6	24	34	no	yes
5	hospital	male	doctor	6	24	34	no	yes
2	hospital	female	midwife	8	8	33	no	yes
2	hospital	female	midwife	2	8	25	no	yes
8	hospital	female	doctor	8	24	34	no	yes
2	hospital	female	midwife	5	8	28	no	yes
6	hospital	male	doctor	8	24	34	no	yes
1	hospital	male	midwife	4	8	29	no	yes
6	hospital	male	doctor	8	18	34	no	yes
4	hospital	male	doctor	6	24	32	no	no
6	hospital	female	doctor	6	12	32	no	yes
2	hospital	female	midwife	5	8	28	no	yes
2	hospital	male	midwife	4	12	28	no	yes
4	hospital	male	doctor	7	24	32	no	yes
2	hospital	male	midwife	6	8	30	no	yes
2	hospital	male	doctor	4	24	28	no	yes
5	hospital	male	doctor	4	24	30	no	yes
1	hospital	female	midwife	5	8	29	no	yes
2	hospital	female	midwife	5	8	29	no	yes
2	hospital	male	midwife	4	8	30	no	yes
3	hospital	female	doctor	6	24	30	no	yes
6	hospital	male	doctor	3	24	28	no	yes
1	hospital	female	midwife	5	8	29	no	yes
1	hospital	male	doctor	3	24	28	no	yes
1	hospital	female	midwife	4	8	28	no	yes
5	hospital	male	doctor	4	24	29	no	yes
4	hospital	female	doctor	3	24	28	no	yes
1	hospital	female	midwife	3	8	27	no	yes
2	hospital	female	midwife	3	8	27	no	yes
2	hospital	female	doctor	3	24	28	no	yes
1	hospital	male	midwife	3	8	27	no	yes
1	hospital	female	midwife	5	12	28	no	yes
4	hospital	male	doctor	3	12	29	no	yes
1	healthcente	female	midwife	6	15	30	yes	yes
1	healthcente	female	midwife	4	15	28	no	no
1	healthcente	male	midwife	4	8	29	no	yes
1	healthcente	male	midwife	6	8	30	no	yes
1	healthcente	female	midwife	5	15	29	no	yes
1	healthcente	female	midwife	6	8	29	no	yes
1	healthcente	female	midwife	4	8	25	yes	yes
1	healthcente	female	midwife	2	8	21	no	yes
1	healthcente	female	midwife	2	8	21	no	yes
1	healthcente	female	midwife	4	8	25	no	yes
1	healthcente	female	midwife	4	15	25	no	yes

1	healthcente	female	midwife	5	15	29	no	yes
1	healthcente	male	healthoffice	4	8	28	no	yes
1	healthcente	female	midwife	5	15	29	yes	yes
1	healthcente	female	midwife	4	15	29	no	yes
1	healthcente	male	midwife	4	8	28	no	yes
1	healthcente	female	midwife	5	8	29	no	yes
1	healthcente	female	midwife	5	15	29	no	yes
1	healthcente	female	midwife	5	8	29	yes	yes
1	healthcente	female	healthoffice	5	8	29	no	yes
1	healthcente	female	midwife	6	15	28	no	yes
1	healthcente	female	midwife	6	8	28	no	yes
1	healthcente	female	midwife	6	15	28	no	yes
1	healthcente	female	midwife	6	15	28	no	yes
1	healthcente	female	midwife	4	8	26	no	yes
1	healthcente	female	midwife	6	8	28	no	yes
1	healthcente	female	healthoffice	5	15	31	no	no
1	healthcente	female	midwife	6	8	28	no	yes
1	healthcente	male	midwife	4	15	29	no	yes
1	healthcente	male	midwife	3	15	28	no	yes
1	healthcente	female	midwife	6	8	29	no	yes
1	healthcente	female	midwife	4	15	27	no	yes
1	healthcente	female	midwife	6	8	28	yes	yes
1	healthcente	male	midwife	5	14	27	yes	yes
1	healthcente	female	midwife	1	14	24	yes	yes
1	healthcente	female	others	5	8	24	yes	yes
1	healthcente	female	midwife	2	15	23	yes	yes
2	healthcente	male	midwife	5	14	27	yes	yes
1	healthcente	male	healthoffice	6	8	32	no	yes
1	healthcente	male	healthoffice	4	15	28	no	yes
1	healthcente	female	midwife	5	15	29	no	yes
1	healthcente	female	healthoffice	5	8	29	yes	yes
1	hospital	male	midwife	4	8	28	no	yes
1	hospital	female	midwife	5	8	29	no	yes
4	hospital	male	midwife	4	8	29	no	yes
3	hospital	male	doctor	4	12	28	no	yes
1	healthcente	female	midwife	4	8	28	no	yes
1	healthcente	male	midwife	3	8	31	no	yes
1	healthcente	female	midwife	3	15	28	no	yes
1	healthcente	female	midwife	4	8	28	yes	yes
1	healthcente	female	midwife	4	15	28	yes	yes
1	hospital	male	midwife	4	8	29	no	yes
1	hospital	male	midwife	4	8	29	no	yes
5	hospital	male	doctor	4	12	29	no	yes
1	hospital	male	midwife	5	8	29	no	yes
4	hospital	male	doctor	6	12	31	yes	yes
4	hospital	male	doctor	5	12	30	no	yes
1	hospital	male	doctor	4	24	29	no	yes
1	hospital	female	midwife	5	8	29	no	yes
1	hospital	female	midwife	5	8	29	yes	yes
2	hospital	male	doctor	4	24	29	no	yes
1	hospital	male	doctor	4	24	29	no	yes
1	hospital	male	doctor	4	24	32	no	yes

1	hospital	male	doctor	4	24	32	no	no
1	hospital	male	midwife	2	8	27	no	yes
2	hospital	female	doctor	4	12	29	no	yes
2	hospital	male	doctor	3	12	28	no	yes
1	hospital	male	midwife	5	8	29	no	yes
2	hospital	male	midwife	4	8	28	no	yes
1	hospital	male	midwife	4	8	28	no	yes
1	hospital	male	doctor	4	12	29	no	yes
1	hospital	male	doctor	4	24	29	no	yes
1	hospital	male	midwife	5	8	29	no	yes
2	hospital	male	midwife	2	8	28	no	yes
4	hospital	male	doctor	5	12	32	no	yes
1	hospital	male	midwife	6	8	30	no	yes
1	hospital	male	midwife	5	8	28	no	yes
2	hospital	male	doctor	3	24	28	no	yes
1	healthcente	female	midwife	4	8	28	no	yes
1	healthcente	female	midwife	4	8	28	yes	yes
1	healthcente	female	midwife	4	15	28	no	yes
1	healthcente	female	midwife	3	8	27	no	yes
1	healthcente	female	midwife	3	8	27	no	yes
1	healthcente	female	midwife	3	8	27	no	yes
1	healthcente	female	midwife	3	15	27	no	yes
1	healthcente	female	healthoffice	4	8	31	no	yes
2	hospital	male	midwife	6	8	32	no	yes
1	hospital	male	midwife	4	8	28	no	yes
1	hospital	female	midwife	3	8	30	no	yes

Q503	Q504	Q505	Q506	Q507	Q508	Q509	Q510	Q511
yes	yes	no	yes	yes		yes	yes	yes
no	yes	no	no	yes	yes	yes	no	no
yes	yes	no	no	yes		no	yes	yes
yes	yes	yes	no	yes		no	yes	yes
yes	no	yes	no	yes		no	yes	yes
yes	yes	no	no	yes		yes	yes	yes
yes	yes	no	no	yes		no	yes	yes
yes	yes	yes	yes	yes		no	yes	yes
yes	yes	no	yes	yes		no	yes	yes
yes	yes	no	yes	yes		yes	yes	yes
yes	yes	yes	yes	yes		no	yes	yes
yes	yes	yes	yes	yes		no	yes	yes
yes	yes	yes	yes	yes		no	yes	yes
yes	yes	no	no	yes		no	yes	yes
yes	yes	no	yes	yes		no	yes	yes
yes	yes	yes	no	yes		no	no	yes
yes	yes	yes	yes	yes		yes	yes	yes
no	yes	yes	yes	yes		no	yes	yes
yes	yes	yes	yes	yes		no	yes	yes
yes	yes	no	yes	yes		yes	no	yes
yes	yes	yes	yes	yes		no	yes	yes
yes	yes	yes	yes	yes		yes	yes	yes
yes	yes	yes	yes	yes		yes	yes	yes
yes	yes	yes	yes	yes	yes	yes	yes	yes
yes	no	yes	yes	yes		yes	yes	yes
yes	no	yes	yes	yes		no	no	yes
no	no	no	yes	no		yes	yes	yes
no	yes	yes	yes	yes		yes	yes	yes
yes	yes	no	yes	yes		no	no	yes
yes	yes	yes	yes	yes		no	yes	yes
yes	yes	no	no	yes		no	no	yes
no	yes	yes	yes	yes		no	yes	yes
yes	yes	yes	no	yes	no	no	yes	yes
no	yes	no	no	yes		no	yes	yes
yes	yes	no	no	yes		no	yes	yes
yes	yes	yes	yes	yes		no	yes	yes
yes	yes	yes	no	yes		no	yes	yes
yes	yes	yes	yes	yes		no	yes	yes
yes	yes	yes	no	yes		no	yes	yes
yes	yes	yes	yes	yes	yes	no	yes	yes
yes	yes	yes	no	yes	no	no	no	yes
yes	yes	yes	yes	yes		yes	yes	no
yes	yes	yes	yes	yes		yes	yes	yes
yes	yes	no	yes	yes	yes	yes	yes	yes
yes	yes	yes	yes	yes		yes	no	yes
yes	yes	yes	no	yes		yes	no	yes
no	no	yes	no	no		no	yes	yes
yes	yes	no	no	yes		no	yes	yes
yes	yes	yes	yes	yes		yes	yes	yes
yes	yes	yes	yes	yes		yes	no	yes
yes	yes	yes	no	no		no	yes	yes

yes	yes	yes	no	yes	yes	no	no	yes
yes	yes	yes	yes	yes		no	yes	yes
yes	yes	yes	yes	yes		yes	yes	yes
yes	yes	yes	yes	yes	no	no	yes	yes
yes	yes	yes	no	yes		no	no	yes
yes	yes	yes	yes	yes		yes	yes	yes
yes	yes	yes	yes	yes		yes	yes	yes
yes	yes	yes	yes	yes		yes	yes	yes
yes	yes	yes	yes	yes		yes	yes	yes
yes	yes	yes	yes	yes		yes	yes	yes
yes	yes	yes	yes	yes		yes	yes	yes
yes	yes	yes	yes	yes		yes	yes	yes
yes	yes	yes	yes	yes		yes	yes	yes
yes	yes	no	no	yes	no	no	yes	yes
yes	yes	yes	yes	yes	no	yes	no	yes
yes	yes	no	no	yes		no	yes	yes
yes	no	no	no	no		no	yes	yes
yes	yes	yes	no	yes	yes	no	yes	yes
yes	yes	no	yes	yes		yes	yes	yes
yes	yes	no	yes	yes		no	yes	yes
yes	yes	no	yes	yes		yes	yes	yes
yes	yes	yes	yes	yes		yes	yes	yes
yes	yes	no	yes	yes		no	yes	yes
yes	yes	yes	yes	yes		yes	yes	yes
yes	yes	yes	yes	yes		yes	yes	yes
yes	yes	no	no	no	no	yes	yes	no
yes	yes	yes	yes	yes		yes	yes	yes
yes	yes	no	no	yes		yes	yes	yes
yes	yes	yes	no	yes	no	yes	yes	yes
yes	yes	yes	no	no	no	yes	yes	yes
yes	yes	yes	yes	yes	yes	yes	yes	yes
yes	yes	yes	yes	yes		yes	yes	yes
yes	yes	yes	no	yes	no	yes	yes	yes
yes	yes	yes	yes	yes		yes	no	yes
yes	yes	no	yes	yes		yes	yes	yes
yes	yes	no	yes	yes		yes	yes	yes
yes	yes	yes	no	yes		yes	yes	yes
yes	yes	yes	no	yes	no	yes	no	yes
yes	yes	yes	no	no		yes	yes	yes
yes	yes	no	no	yes		no	yes	yes
yes	no	no	no	yes		no	yes	yes
yes	yes	no	yes	yes		yes	yes	yes
yes	yes	yes	yes	yes		yes	no	yes
yes	yes	yes	yes	yes		yes	yes	yes
yes	yes	yes	yes	yes		yes	yes	yes
no	yes	no	no	no		yes	yes	yes
yes	yes	no	yes	yes		yes	yes	yes
yes	yes	no	yes	yes		yes	yes	yes
yes	yes	yes	yes	yes		yes	yes	no
no	yes	yes	yes	no		no	yes	yes
yes	yes	yes	yes	yes	no	no	yes	yes
yes	yes	no	no	no		no	yes	yes

yes	yes	no	no	yes		no	yes	yes
yes	yes	no	no	yes		no	yes	yes
yes	yes	yes	yes	no		no	yes	yes
yes	yes	no	no	yes		no	yes	yes
yes	yes	no	yes	yes		yes	yes	yes
yes	yes	yes	yes	yes		yes	yes	yes
yes	yes	yes	no	yes		yes	yes	yes
yes	yes	yes	no	no		yes	yes	yes
yes	yes	yes	yes	yes		no	yes	yes
yes	yes	yes	no	yes		yes	yes	yes
yes	yes	yes	yes	yes		yes	yes	yes
yes	yes	yes	yes	yes		yes	yes	yes
yes	yes	yes	no	yes		yes	yes	yes
yes	yes	yes	yes	yes		yes	yes	yes
yes	yes	yes	yes	yes		no	yes	yes
yes	yes	no	no	yes		yes	yes	yes
yes	no	no	yes	yes		no	no	yes
yes	yes	no	yes	no		no	yes	yes
yes	yes	no	no	yes		yes	yes	yes
yes	yes	yes	yes	yes		no	no	yes
yes	yes		yes	yes		yes	yes	yes
yes	yes	no	yes	yes		yes	yes	yes
yes	yes	yes	no	no		yes	yes	yes
yes	yes	no	yes	yes		yes	yes	yes
yes	yes	no	yes	yes		yes	yes	yes
yes	yes	yes	yes	yes		yes	yes	yes
yes	yes	yes	yes	yes		yes	yes	yes
yes	yes	yes	yes	no		yes	yes	yes
yes	yes	yes	yes	yes		yes	yes	yes
yes	yes	yes	yes	no		yes	no	yes
yes	yes	yes	yes	yes		yes	yes	yes
yes	yes	yes	yes	yes		yes	no	yes
yes	yes	no	yes	no	no	yes	yes	yes
yes	yes	yes	yes	yes		yes	no	no
yes	yes	yes	yes	yes		yes	yes	yes
yes	yes		yes	yes		no	yes	yes
yes	yes		yes	no	no	yes	yes	yes
yes	yes		no	yes		yes	yes	yes
yes	no	no	yes	yes		no	yes	yes
yes	yes	yes	yes	yes		yes	yes	yes
yes	yes	yes	yes	yes		yes	yes	yes
yes	yes		yes	yes		yes	yes	yes
yes	yes	no	yes	yes		yes	yes	yes
yes	yes	no	yes	yes		yes	yes	yes
yes	yes	yes	no	yes		no	no	no
yes	yes	no	yes	yes		yes	yes	yes
no	no	yes	yes	yes	yes	yes	yes	yes
yes	yes	no	yes	yes		yes	yes	yes
yes	yes	yes	yes	yes		yes	yes	yes
yes	yes	no	yes	yes		yes	yes	yes
yes	yes	no	yes	yes		yes	yes	yes
yes	yes	yes	yes	yes		yes	yes	yes

yes	yes		yes	yes		yes	no	yes
yes	yes	yes	no	no		yes	yes	yes
no	no		no	yes		yes	no	yes
yes	yes	yes	yes	yes	yes	yes	yes	yes
yes	yes	yes	yes	yes		yes	yes	yes
yes	yes	yes	no	no		yes	yes	yes
yes	yes	yes	yes	yes		yes	yes	yes
yes	yes	yes	yes	yes	yes	yes	yes	yes
yes	yes	yes	yes	yes		yes	yes	yes
yes	yes	yes	yes	yes		yes	no	yes
yes	yes	yes	yes	yes	yes	yes	yes	yes
yes	yes	yes	yes	yes	yes	yes	yes	yes
yes	yes	yes	yes	yes		yes	yes	yes
yes	yes	yes	yes	yes		yes	yes	yes
yes	yes	yes	yes	yes	no	yes	yes	yes
yes	yes	no	yes	yes		yes	no	yes
yes	yes	no	yes	yes	yes	yes	yes	yes
yes	yes		yes	yes		yes	no	yes
yes	yes		yes	no		no	no	yes
yes	no		yes	yes		yes	no	yes
yes	yes		yes	no		yes	yes	yes
yes	yes		yes	yes		yes	yes	yes
yes	yes	yes	yes	yes		yes	yes	yes
yes	yes		yes	yes		yes	yes	yes
yes	yes		yes	yes		yes	yes	yes
yes	yes		no	yes		yes	yes	yes
yes	yes		yes	yes		yes	yes	yes
yes	no		no	no		yes	yes	yes
yes	yes		yes	yes		yes	yes	yes
yes	yes	yes	yes	yes		no	no	no
yes	yes	yes	yes	yes		no	no	no
yes	yes	yes	yes	yes		yes	yes	yes
yes	yes	yes	yes	yes		yes	yes	yes
yes	yes	yes	yes	yes		yes	yes	yes
yes	yes		yes	yes		yes	yes	yes
yes	yes	yes	yes	yes		yes	yes	yes
yes	yes	yes	yes	yes		yes	yes	yes
yes	yes	yes	yes	yes		yes	yes	yes
yes	yes	yes	yes	yes		no	yes	yes
yes	yes	yes	no	yes		yes	yes	yes
yes	yes	yes	yes	yes		yes	yes	yes
no	no		no	no		yes	yes	yes
yes	no	yes	no	no		yes	yes	yes
yes	yes	yes	yes	yes		yes	yes	yes
yes	yes	yes	yes	yes		yes	yes	yes
yes	yes	yes	yes	yes		yes	yes	yes
yes	yes	yes	yes	yes		yes	no	no
yes	yes	yes	no	yes		yes	no	no
yes	yes	yes	yes	yes	yes	yes	no	no
yes	yes	yes	yes	yes		yes	yes	yes
yes	yes	yes	yes	yes		yes	yes	yes
yes	yes	yes	yes	yes		yes	no	no

yes	yes		yes	no		yes	no	no
yes	yes	no	yes	no		yes	yes	no
yes	yes	no	yes	no		yes	yes	no
yes	yes	yes	yes	yes		yes	yes	yes
yes	yes	no	yes	yes		yes	no	no
yes	yes	yes	yes	yes		yes	yes	yes
yes	yes	yes	yes	yes		yes	yes	yes
yes	yes	yes	yes	yes		yes	yes	yes
yes	yes	yes	yes	yes		yes	yes	yes
yes	yes	yes	yes	yes		yes	yes	yes
yes	yes	yes	yes	yes		yes	yes	yes
yes	no		yes	no		no	yes	yes
yes	yes	yes	yes	yes		no	yes	yes
yes	no		yes	yes		yes	yes	yes
yes	yes	yes	no	no		no	yes	yes
yes	yes	yes	yes	yes		yes	yes	yes
yes	yes		yes	yes		no	yes	yes
yes	yes	yes	yes	yes	yes	yes	yes	yes
yes	yes	yes	yes	yes		yes	yes	yes
yes	yes	yes	yes	yes		yes	yes	yes
yes	yes	yes	yes	yes		yes	yes	yes
yes	yes	yes	yes	yes		yes	yes	yes
yes	yes	yes	no	yes	yes	yes	yes	yes
yes	yes	yes	no	yes		yes	yes	yes
yes	yes	yes	no	yes		yes	yes	yes
yes	yes	yes	yes	yes		yes	yes	yes
yes	yes	yes	no	yes		yes	yes	yes
yes	yes	no	no	no		no	no	yes
yes	yes	yes	yes	yes		yes	yes	yes
yes	yes	yes	no	yes		yes	yes	yes
yes	yes	yes	no	no		yes	yes	yes
yes	yes	no	yes	no		yes	yes	yes
yes	yes	yes	yes	no		no	no	yes
yes	no	yes	yes	yes	yes	yes	yes	yes
yes	yes	yes	yes	yes		yes	yes	yes
yes	yes	yes	yes	yes	yes	yes	yes	yes
yes	yes	yes	yes	yes		yes	yes	yes
yes	yes	yes	yes	yes		yes	yes	yes
yes	no		yes	yes		yes	yes	yes
yes	yes		yes	yes		yes	yes	yes
yes	no		no	no		yes	yes	yes
yes	yes		no	yes		no	yes	yes
yes	no		yes	yes		yes	yes	yes
no	no		no	no		yes	yes	yes
yes	yes	yes	yes	yes		yes	yes	yes
yes	yes	yes	yes	yes		yes	yes	yes
yes	no	no	no	no		yes	yes	yes
yes	yes	yes	yes	yes		yes	yes	yes
yes	yes	no	yes	yes		no	yes	yes
yes	yes	yes	yes	yes		yes	yes	yes
yes	yes	yes	no	no		yes	yes	yes
yes	yes		yes	yes	no	no	yes	yes

yes	no		no	yes	no	no	yes	yes
yes	yes	yes	yes	yes	yes	yes	yes	yes
yes	no		no	no	no	yes	yes	yes
yes	yes	no	yes	yes		no	yes	yes
yes	yes	yes	yes	yes		yes	yes	yes
yes	yes	yes	yes	yes		yes	yes	yes
yes	yes	yes	yes	yes		yes	yes	yes
yes	yes	yes	yes	yes		yes	yes	yes
yes	no	yes	no	yes		yes	yes	yes
yes	no	yes	no	no		yes	yes	yes
yes	yes	yes	yes	yes		yes	yes	yes
yes	yes		no	yes		no	yes	yes
yes	yes	yes	yes	yes		yes	yes	yes
yes	yes	no	yes	yes	no	no	yes	yes
yes	yes	yes	yes	yes		no	yes	yes
yes	yes	yes	yes	yes		yes	yes	yes
yes	yes		no	yes		no	yes	yes
yes	no		yes	no		yes	yes	yes
yes	yes		yes	yes		no	yes	yes
yes	yes	no	yes	yes	no	yes	yes	yes
yes	yes	yes	yes	yes		yes	yes	yes
yes	yes	yes	yes	yes		yes	yes	yes
yes	no	yes	no	no	no	yes	yes	yes
yes	yes	yes	yes	yes		no	yes	yes
yes	no	yes	yes	yes		no	yes	yes
yes	yes	yes	yes	yes		yes	yes	yes
yes	yes	yes	yes	yes		yes	yes	yes
yes	yes	yes	yes	yes		no	yes	yes
yes	yes	yes	yes	yes		yes	yes	yes
yes	yes	yes	no	no		no	yes	yes
yes	yes	yes	yes	yes		yes	yes	yes
yes	yes	yes	yes	yes		yes	yes	yes
yes	yes	yes	yes	yes		yes	yes	yes
yes	yes	no	yes	yes		no	yes	yes
yes	yes	yes	yes	yes		yes	yes	yes
yes	yes	yes	yes	yes		yes	yes	yes
yes	yes	no	no	no		no	yes	yes
yes	yes	yes	yes	yes		no	yes	yes
yes	yes	yes	yes	yes		yes	yes	yes
yes	yes	yes	yes	yes		yes	yes	yes
yes	yes	yes	no	yes		yes	yes	yes
yes	yes	yes	yes	yes	yes	yes	yes	yes
no	no	yes	no	no		yes	no	yes
yes	yes	yes	yes	yes		yes	yes	yes
yes	yes	yes	no	no		yes	yes	yes
yes	yes	yes	yes	yes	yes	yes	yes	yes
yes	yes	yes	no	yes		yes	yes	yes
yes	yes	yes	yes	yes		yes	yes	yes
yes	yes	yes	no	yes		yes	yes	yes
yes	yes	yes	yes	yes		yes	yes	yes
yes	yes	yes	yes	no		yes	yes	no
yes	yes	yes	yes	yes		yes	yes	yes

yes	yes	yes	yes	yes		yes	yes	yes
yes	yes	yes	yes	yes		yes	yes	yes
yes	yes	yes	yes	yes		yes	yes	yes
yes	yes	yes	yes	yes		yes	yes	yes
yes	yes	no	yes	yes		yes	yes	yes
yes	yes	yes	yes	yes		yes	yes	yes
yes	yes	yes	yes	yes	yes	yes	yes	yes
yes	yes	yes	yes	yes		yes	yes	yes
yes	yes	yes	yes	yes		yes	yes	yes
yes	yes	yes	yes	yes		yes	yes	yes
yes	yes	yes	yes	no		yes	yes	yes
yes	yes	yes	yes	yes		yes	yes	yes
yes	yes	yes	yes	yes		yes	yes	yes
yes	yes	yes	yes	yes		yes	yes	yes
yes	yes	yes	yes	yes		yes	yes	yes
yes	no	yes	no	no	no	yes	yes	yes
yes	yes	yes	yes	yes		yes	yes	yes
yes	yes	yes	yes	yes		yes	yes	yes
yes	yes	yes	no	no	no	yes	yes	yes
yes	yes	yes	yes	yes		yes	yes	yes
yes	yes	yes	yes	yes		yes	yes	yes
yes	yes	yes	yes	yes		yes	yes	yes
yes	yes	yes	yes	yes		yes	yes	yes
yes	yes	yes	yes	yes	yes	yes	yes	yes
yes	yes	yes	yes	yes		yes	yes	yes
yes	yes	yes	yes	no		yes	yes	yes
yes	yes	yes	yes	yes		yes	yes	yes
yes	yes	yes	yes	yes		yes	yes	yes
yes	yes	yes	yes	yes		yes	yes	yes
no	yes	no	no	no		yes	yes	yes
yes	yes	yes	yes	yes		yes	yes	yes
yes	yes	yes	yes	no		no	yes	yes
yes	yes	no	yes	yes		yes	yes	no
yes	yes	no	yes	no		no	yes	yes
yes	yes	no	yes	no		no	yes	yes
yes	yes	yes	yes	yes		yes	yes	yes
yes	yes	yes	yes	yes		yes	yes	yes
yes	yes	yes	yes	yes		yes	yes	yes
yes	yes	yes	yes	yes	no	yes	yes	yes
yes	yes	yes	yes	yes		yes	yes	yes
yes	yes	yes	yes	no	no	no	yes	yes
yes	yes	yes	yes	no		no	yes	yes
yes	yes	yes	yes	no		no	yes	yes
yes	yes	no	yes	no		no	yes	yes
yes	yes	yes	yes	no		no	yes	yes
yes	yes	no	yes	no		no	yes	yes
yes	yes	yes	yes	no		no	yes	yes
yes	yes	yes	yes	yes		yes	yes	yes
yes	yes	yes	yes	yes		yes	yes	yes
yes	yes	yes	yes	yes		no	yes	yes
yes	yes	yes	no	no		no	yes	yes
yes	yes	yes	yes	yes		no	yes	yes

yes	yes	yes	yes	yes		no	yes	yes
yes	yes	yes	yes	yes		yes	yes	yes
yes	yes	no	no	yes	no	no	yes	yes
yes	yes	yes	no	no		no	yes	yes
yes	yes	yes	no	yes		no	yes	yes
yes	yes	no	yes	no		no	yes	yes
yes	yes	no	yes	no		no	yes	yes
yes	yes	no	yes	no		no	yes	yes
yes	yes	no	yes	yes		yes	yes	yes
yes	yes	no	no	no		no	yes	yes
yes	yes	no	no	no		no	yes	yes
yes	yes	no	yes	no		no	yes	yes
yes	yes	yes	no	yes		no	yes	yes
yes	yes	no	no	yes		no	yes	yes
yes	yes	yes	no	yes		yes	no	yes
yes	yes	yes	yes	yes		yes	yes	yes
yes	yes	yes	yes	yes		yes	yes	yes
yes	yes	no	yes	no		yes	yes	yes
yes	yes	yes	yes	yes		yes	yes	yes
yes	yes	yes	yes	yes		yes	yes	yes
yes	yes	yes	yes	yes		yes	yes	yes
yes	yes	yes	yes	yes		yes	yes	yes
yes	yes	yes	yes	yes		yes	yes	yes
yes	yes	no	no	yes	no	yes	yes	yes
yes	yes	no	no	yes		yes	yes	yes
yes	yes	yes	no	yes	no	no	yes	yes

Q512	Q513	Q514	Q515	Q516	Q517	Q518	Q519	Q520
yes	yes	yes	no	yes	yes	yes		no
yes	yes	yes	no	yes	yes	yes	no	yes
yes	yes	yes	no	yes	yes	yes		yes
yes	yes	yes	yes	yes	yes	yes		yes
yes	yes	no	no	yes	yes	yes		yes
yes	yes	yes	no	yes	yes	yes		yes
yes	yes	yes	no	yes	yes	yes		yes
yes	yes	yes	yes	yes	yes	yes	no	yes
yes	no	yes	yes	yes	yes	yes		no
yes	yes	yes	no	yes	yes	yes		yes
no	yes	yes	yes	yes	yes	yes		yes
yes	yes	yes	no	yes	yes	yes		yes
yes	yes	yes	yes	yes	yes	yes		yes
yes	yes	yes	no	yes	yes	yes		yes
yes	yes	yes	no	yes	yes	yes		yes
yes	yes	yes	yes	yes	yes	yes		yes
yes	yes	yes	no	yes	yes	yes		yes
yes	yes	yes	no	yes	yes	yes	no	yes
yes	yes	no	no	yes	yes	yes		yes
yes	yes	yes	yes	yes	yes	yes		yes
yes	yes	yes	no	yes	yes	yes		yes
yes	yes	yes	no	yes	yes	yes		no
yes	yes	yes	yes	yes	yes	yes		yes
yes	yes	yes	no	yes	yes	yes		yes
yes	yes	yes	no	yes	yes	yes		yes
yes	yes	yes	no	yes	yes	yes		yes
yes	no	yes	no	yes	yes	yes		no
yes	yes	yes	yes	yes	yes	yes		yes
yes	yes	yes	yes	yes	yes	yes		yes
yes	yes	yes	no	yes	yes	yes		no
yes	yes	yes	no	yes	yes	yes		yes
yes	yes	yes	no	yes	yes	yes		yes
yes	yes	yes	yes	yes	yes	yes		yes
yes	yes	yes	no	yes	yes	yes		yes
yes	yes	yes	no	yes	yes	yes		yes
yes	yes	yes	no	yes	yes	yes		yes
yes	yes	yes	no	yes	yes	yes		yes
yes	yes	yes	no	yes	yes	yes		yes
no	yes	yes	yes	yes	yes	yes		yes
yes	yes	yes	yes	yes	yes	yes	no	yes
no	no	no	no	yes	yes	yes	yes	yes
yes	yes	yes	yes	yes	yes	yes		yes
yes	yes	yes	no	yes	yes	yes		yes
yes	yes	yes	yes	yes	yes	yes	yes	yes
yes	yes	yes	no	yes	yes	yes		
yes	yes	yes	no	yes	yes	yes		
yes	yes	yes	no	yes	yes	yes	no	no
yes	no	no	no	yes	yes	yes		
yes	yes	yes	no	yes	yes	yes		yes
no	yes	no	no	yes	yes	yes		yes
yes	yes	yes	no	yes	yes	yes		yes

yes	yes	yes	no	yes	yes	yes	no	yes
yes	yes	yes	no	yes	yes	yes		yes
yes	yes	yes	no	yes	yes	yes		yes
no	yes	yes	yes	yes	yes	yes	no	
yes	yes	yes	yes	yes	yes	yes		yes
yes	yes	yes	yes	yes	yes	yes		yes
yes	yes	yes	yes	yes	yes	yes		yes
yes	yes	yes	yes	yes	yes	yes		yes
yes	yes	yes	yes	yes	yes	yes		yes
yes	yes	yes	yes	yes	yes	yes		yes
yes	yes	yes	yes	yes	yes	yes		yes
yes	yes	yes	yes	yes	yes	yes		yes
yes	yes	yes	yes	yes	yes	yes		yes
yes	yes	yes	no	yes	yes	yes	no	yes
yes	yes	yes	yes	yes	yes	yes	no	yes
yes	yes	yes	no	yes	yes	yes		no
yes	no	no	no	yes	yes	yes		yes
yes	yes	yes	no	yes	yes	yes		no
yes	yes	yes	yes	yes	yes	yes		yes
yes	yes	yes	yes	yes	yes	yes		yes
yes	yes	yes	no	yes	yes	yes		no
yes	yes	yes	yes	yes	yes	yes		yes
yes	yes	yes	no	yes	yes	yes		yes
yes	yes	yes	yes	yes	yes	yes		no
yes	yes	yes	no	yes	yes	yes		yes
yes	yes	no	no	yes	yes	yes	yes	yes
yes	yes	yes	yes	yes	yes	yes		
yes	yes	yes	no	yes	yes	yes		
yes	yes	yes	no	yes	yes	yes	no	yes
yes	yes	yes	no	yes	yes	yes	yes	yes
yes	yes	yes	yes	yes	yes	yes	no	
yes	yes	yes	yes	yes	yes	yes		yes
yes	yes	yes	no	yes	yes	yes	yes	yes
yes	yes	yes	no	yes	yes	yes		yes
yes	no	no	no	yes	yes	yes		no
yes	yes	yes	no	yes	yes	yes		no
yes	yes	yes	no	yes	yes	yes		yes
yes	yes	yes	no	yes	yes	yes	no	yes
yes	yes	yes	no	yes	yes	yes		yes
yes	yes	yes	no	yes	yes	yes	no	no
yes	yes	yes	no	no	no	no		yes
yes	yes	yes	no	no	no	no		yes
yes	yes	yes	no	no	no	yes		yes
yes	yes	yes	yes	yes	yes	yes		yes
yes	yes	yes	yes	yes	yes	yes		yes
yes	yes	yes	no	yes	yes	no		yes
yes	yes	yes	no	yes	yes	yes		yes
yes	yes	yes	no	yes	yes	yes		yes
yes	yes	yes	no	yes	yes	yes		yes
yes	yes	yes	no	yes	yes	yes		yes
yes	yes	yes	no	yes	yes	yes		yes
yes	yes	yes	no	yes	yes	yes		no

yes	yes	yes	no	yes	yes	yes		yes
yes	yes	yes	no	yes	yes	yes		yes
yes	yes	yes	no	yes	yes	yes		yes
yes	yes	yes	no	yes	yes	yes		no
yes	yes	yes	no	yes	yes	yes		yes
yes	yes	yes	no	yes	yes	yes		yes
yes	yes	yes	no	yes	yes	yes		yes
yes	yes	yes	no	yes	yes	yes		yes
yes	yes	yes	no	yes	yes	yes		yes
yes	yes	yes	no		no	no		yes
yes	yes	yes	no	yes	yes	yes		
yes	yes	yes	no	yes	yes	yes	no	yes
yes	yes	yes	no	yes	yes	yes		yes
yes	yes	yes	no	yes	yes	yes		yes
yes	yes	yes	no	yes	yes	yes		yes
yes	yes	yes	no		yes	yes		yes
yes	no	no	no	yes	yes	yes		yes
yes	yes	yes	no		yes	yes		yes
yes	yes	yes	no	yes	yes	yes		yes
yes	yes	yes	no		yes	yes		yes
yes	yes	yes	no		yes	yes		yes
yes	yes	yes	no		yes	yes		yes
yes	yes	yes	no		yes	yes		yes
yes	yes	yes	no	yes	yes	yes		yes
yes	yes	yes	no	yes	yes	yes		no
yes	yes	yes	no	yes	yes	yes		yes
yes	yes	yes	yes	yes	yes	yes		yes
yes	no	no	no	yes	yes	yes		yes
yes	no	no	no	yes	yes	no		yes
yes	yes	yes	no	yes	yes	yes		no
yes	yes	yes	yes	yes	yes	yes		yes
yes	yes	yes	no	yes	yes	yes		no
yes	yes	yes	no	yes	yes	no	yes	yes
yes	no	no	yes	yes	yes	yes		yes
yes	no	no	no		no	no		yes
yes	yes	yes	no		yes	yes		yes
yes	yes	yes	no		yes	yes	yes	yes
yes	yes	yes	no		yes	yes		yes
yes	yes	yes	no		yes	yes		yes
yes	yes	yes	no		yes	yes		yes
yes	no	no	no		yes	yes		yes
yes	yes	yes	no	yes	yes	yes		yes
yes	yes	yes	no	yes	yes	yes		yes
yes	yes	yes	yes	yes	yes	yes		yes
yes	yes	yes	no	yes	yes	yes		yes
yes	yes	yes	yes	yes	yes	yes		yes
yes	yes	yes	no	yes	yes	no	yes	yes
yes	yes	yes	yes	yes	yes	yes		yes
yes	yes	yes	no	yes	yes	yes		yes
yes	yes	yes	no	yes	yes	yes		yes
yes	yes	yes	yes	yes	yes	yes	no	yes
yes	yes	yes	no	yes	no	yes		yes

yes	yes	yes	no	yes	yes	yes		yes
yes	yes	no	no	yes	yes	yes		
yes	yes	yes	no	yes	yes	no		yes
yes	yes	yes	yes	yes	yes	yes	yes	yes
yes	yes	yes	no	yes	yes	yes		yes
yes	no	no	no	yes	yes	yes		no
yes	yes	yes	yes	yes	yes	yes		yes
yes	yes	yes	yes	yes	yes	yes	yes	yes
yes	yes	yes	yes	yes	yes	yes		
yes	yes	yes	yes	yes	yes	yes		yes
yes	yes	yes	yes	yes	yes	yes	yes	yes
yes	no	no	no	no	yes	yes	no	no
yes	yes	yes	yes	yes	yes	yes		
yes	yes	yes	yes	yes	yes	yes		
yes	yes	yes	yes	yes	yes	yes	no	yes
yes	yes	yes	yes	yes	yes	yes		no
yes	yes	yes	no	yes	yes	yes	no	no
yes	yes	yes	no	yes	yes	yes		yes
yes	yes	yes	no	yes	yes	yes		no
yes	yes	yes	no	yes	yes	yes		yes
yes	yes	yes	no	yes	yes	yes		
yes	yes	yes	no	yes	yes	yes		yes
yes	yes	yes	no	yes	yes	yes		
yes	yes	yes	no	yes	yes	yes		yes
yes	yes	yes	no	yes	yes	yes		
yes	yes	yes	no		yes	yes		yes
yes	yes	yes	no	yes	yes	yes		yes
yes	no	no	no	yes	yes	yes		no
yes	yes	yes	no	yes	yes	yes		yes
yes	yes	yes	yes	yes	yes	yes		
yes	yes	yes	yes	yes	yes	yes		
yes	yes	yes	no	yes	yes	yes		yes
yes	yes	yes	no	yes	yes	yes		yes
yes	yes	yes	no	yes	yes	yes		no
yes	yes	yes	no	yes	yes	yes		no
yes	yes	yes	yes	yes	yes	yes	no	no
yes	yes	yes	yes	yes	yes	yes		no
yes	yes	yes	no	yes	yes	yes		no
yes	yes	yes	no	yes	yes	yes		yes
yes	yes	yes	yes	yes	yes	yes		yes
yes	yes	yes	yes	yes	yes	yes		yes
yes	no	no	yes	yes	yes	yes		yes
yes	no	no	yes	yes	yes	yes		yes
yes	yes	yes	no	yes	yes	yes		yes
yes	yes	yes	no	yes	yes	yes		
yes	yes	yes	yes	yes	yes	yes		no
yes	yes	yes	no	yes	yes	yes		
yes	yes	yes	no	yes	yes	yes		
yes	yes	yes	no	yes	yes	yes		yes
yes	yes	yes	no	yes	yes	yes		yes
yes	yes	yes	no	yes	yes	yes		yes
yes	no	yes	no	yes	yes	yes		yes

yes	yes	yes	no	yes	yes	yes		yes
yes	no	no	no	yes	yes	yes		
yes	yes	yes	no	yes	yes	yes		
yes	yes	yes	no	yes	yes	yes		
yes	no	yes	no	yes	yes	yes		
yes	yes	yes	no	yes	yes	yes		
yes	yes	yes	no	yes	yes	yes		
yes	yes	yes	no	yes	yes	yes		
yes	yes	yes	no	yes	yes	yes		
yes	yes	yes	no	yes	yes	yes		
yes	yes	yes	no	yes	yes	yes		
no	no	no	no	yes	yes	yes		yes
yes	yes	yes	no	yes	yes	yes		yes
yes	yes	yes	yes	yes	yes	yes		yes
yes	yes	yes	no	yes	yes	yes		no
yes	yes	yes	yes	yes	yes	yes		yes
yes	yes	yes	no	yes	yes	yes		
yes	yes	yes	yes	yes	yes	yes	yes	yes
yes	yes	yes	no	yes	yes	yes		yes
yes	yes	yes	no	yes	no	yes		yes
yes	yes	yes	no	yes	yes	yes		yes
yes	yes	yes	no	yes	yes	no		yes
yes	yes	yes	no	yes	yes	yes	yes	yes
yes	yes	yes	no	yes	yes	yes	no	yes
yes	yes	yes	no	yes	yes	yes		yes
yes	yes	yes	no	yes	yes	yes		yes
yes	yes	yes	no	yes	yes	yes		yes
no	yes	yes	yes	yes	yes	yes		yes
yes	yes	yes	yes	yes	yes	yes		yes
no	yes	yes	no	yes	yes	yes		yes
yes	yes	yes	yes	no	yes	yes		yes
no	no	yes	yes	yes	yes	yes		yes
yes	yes	yes	yes	yes	yes	yes		yes
yes	no	no	no	yes	yes	yes	no	yes
yes	yes	yes	no	yes	yes	yes		yes
yes	yes	yes	no	yes	yes	yes	yes	yes
yes	yes	yes	no	yes	yes	yes		yes
yes	yes	yes	no	yes	yes	yes		yes
yes	no	no	no	yes	yes	yes	yes	yes
yes	yes	yes	no	yes	yes	yes		yes
yes	no	no	no	yes	yes	yes		
yes	no	no	no	yes	yes	yes		yes
yes	yes	yes	no	yes	yes	yes		yes
yes	no	no	no	yes	yes	yes		yes
yes	yes	yes	no	yes	yes	yes		yes
yes	yes	yes	no	yes	yes	yes		yes
yes	no	no	no	yes	yes	yes		yes
yes	yes	yes	no	yes	yes	yes		yes
yes	yes	yes	no	yes	yes	yes		
yes	yes	yes	no	yes	yes	yes		yes
yes	yes	yes	no	yes	yes	yes		yes
yes	yes	yes	no	yes	yes	yes	no	yes

yes	no	no	no	yes	yes	yes	no	yes
yes	yes	yes	no	yes	yes	yes		yes
yes	no	no	no	yes	yes	yes	no	yes
yes	yes	yes	no	yes	yes	yes		
yes	yes	yes	no	yes	yes	yes		yes
yes	yes	yes	no	yes	yes	yes		
yes	yes	yes	no	yes	yes	yes		yes
yes	yes	yes	no	yes	yes	yes		yes
yes	yes	yes	no	yes	yes	yes		yes
yes	no	no	no	yes	yes	yes		yes
yes	yes	yes	no	yes	yes	yes		
yes	yes	yes	no	yes	yes	yes		
yes	yes	yes	no	yes	yes	yes		yes
yes	yes	yes	no	yes	yes	yes	no	yes
yes	yes	yes	no	yes	yes	yes		yes
yes	yes	yes	no	yes	yes	yes		
yes	yes	yes	no	yes	yes	yes		yes
yes	no	no	no	yes	yes	yes		yes
no	yes	yes	no	yes	yes	yes		yes
yes	yes	yes	no	yes	yes	yes	yes	yes
yes	yes	yes	no	yes	yes	yes		
yes	yes	yes	no	yes	yes	yes		yes
yes	yes	no	no	yes	yes	yes	no	yes
yes	yes	yes	no	yes	yes	yes		yes
yes	no	no	no	yes	yes	yes		yes
yes	yes	yes	no	yes	yes	yes		
yes	yes	yes	no	yes	yes	yes		
yes	yes	yes	no	yes	yes	yes		yes
yes	yes	yes	no	yes	yes	yes		yes
yes	yes	yes	no	yes	yes	yes		yes
yes	yes	yes	no	yes	yes	yes		
yes	yes	yes	no	yes	yes	yes		
yes	yes	yes	no	yes	yes	yes		
yes	yes	yes	no	yes	yes	yes		yes
yes	yes	yes	no	yes	yes	yes		yes
yes	yes	yes	no	yes	yes	yes		
yes	yes	yes	no	yes	yes	yes		yes
yes	yes	yes	no	yes	yes	yes		yes
yes	yes	yes	no	yes	yes	yes		
yes	yes	yes	no	yes	yes	yes		
yes	yes	yes	no	yes	yes	yes		yes
yes	yes	yes	no	yes	yes	yes	yes	yes
yes	no	no	no	yes	yes	yes		
yes	yes	yes	yes	yes	yes	yes		yes
yes	yes	yes	yes	yes	yes	yes		
yes	yes	yes	no	yes	yes	yes	no	yes
yes	yes	yes	no	yes	yes	yes		no
yes	yes	yes	yes	yes	yes	yes		no
yes	yes	yes	no	yes	yes	yes		
yes	yes	yes	no	yes	yes	yes		
yes	yes	yes	no	yes	yes	yes		yes
yes	yes	yes	yes	yes	yes	yes		yes

yes	yes	yes	no	yes	yes	yes		yes
yes	yes	yes	no	yes	yes	yes		yes
yes	yes	yes	no	yes	yes	yes		
yes	yes	yes	yes	yes	yes	yes		yes
yes	yes	yes	no	yes	yes	yes	no	no
yes	yes	yes	no	yes	yes	yes		
yes	yes	yes	no	yes	yes	yes	no	yes
yes	yes	yes	yes	yes	yes	yes		yes
yes	yes	yes	yes	yes	yes	yes		yes
yes	yes	yes	yes	yes	yes	yes		yes
yes	no	no	no	yes	yes	yes		yes
yes	yes	yes	no	yes	yes	yes		yes
yes	yes	no	yes	yes	yes	yes		
yes	yes	yes	yes	yes	yes	yes		
yes	yes	yes	yes	yes	yes	yes		yes
yes	yes	no	no	yes	yes	yes	no	yes
yes	yes	yes	no	yes	yes	yes		
yes	yes	yes	yes	yes	yes	yes		yes
yes	yes	yes	no	yes	yes	yes	no	yes
yes	yes	yes	yes	yes	yes	yes		yes
yes	yes	yes	yes	yes	yes	yes		yes
yes	no	no	yes	yes	yes	yes	no	no
yes	yes	yes	yes	yes	yes	yes		no
yes	yes	yes	no	yes	yes	yes	yes	no
yes	yes	yes	yes	yes	yes	yes		yes
yes	yes	yes	yes	yes	yes	yes		no
yes	yes	yes	yes	yes	yes	yes		yes
yes	yes	yes	yes	yes	yes	yes		yes
yes	yes	yes	yes	yes	yes	yes		
yes	yes	yes	no	yes	yes	yes		yes
yes	yes	yes	no	yes	yes	yes		yes
yes	yes	yes	no	yes	yes	yes		
yes	yes	yes	no	yes	yes	yes		yes
yes	yes	yes	no	yes	yes	yes		yes
yes	yes	yes	no	yes	yes	yes		yes
yes	yes	yes	yes	yes	yes	yes		
yes	yes	yes	yes	yes	yes	yes		yes
yes	yes	yes	yes	yes	yes	yes		
yes	yes	yes	yes	yes	yes	yes	no	yes
yes	yes	yes	yes	yes	yes	yes		yes
yes	yes	yes	no	yes	yes	yes	no	yes
yes	yes	yes	no	yes	yes	yes		
yes	yes	yes	no	yes	yes	yes		yes
yes	yes	yes	no	yes	yes	yes		
yes	yes	yes	no	yes	yes	yes		yes
yes	yes	yes	no	yes	yes	yes		yes
yes	no	no	no	yes	yes	yes		no
yes	yes	yes	no	yes	yes	yes		yes
yes	yes	yes	no	yes	yes	yes		yes
yes	yes	yes	no	yes	yes	yes		
yes	yes	yes	no	yes	yes	yes		yes
yes	yes	yes	no	yes	yes	yes		yes

yes	yes	yes	no	yes	yes	yes		
yes	yes	yes	no	yes	yes	yes		yes
yes	yes	yes	no	yes	yes	yes	no	yes
yes	yes	yes	no	yes	yes	yes		yes
yes	yes	yes	no	yes	yes	yes		yes
yes	yes	yes	no	yes	yes	yes		yes
yes	yes	yes	no	yes	yes	yes		yes
yes	yes	yes	no	yes	yes	yes		yes
yes	yes	yes	no	yes	yes	yes		yes
yes	yes	yes	no	yes	yes	yes		yes
yes	yes	yes	no	yes	yes	yes		
yes	yes	yes	no	yes	yes	yes		yes
yes	yes	yes	no	yes	yes	yes		no
yes	yes	yes	no	yes	yes	yes		no
yes	yes	yes	no	yes	yes	yes	no	yes
yes	yes	yes	no	yes	yes	yes		yes
yes	yes	yes	no	yes	yes	yes		yes
yes	yes	no	no	yes	yes	yes		yes
yes	yes	yes	no	yes	yes	yes		yes
yes	yes	yes	yes	yes	yes	yes		yes
yes	yes	yes	yes	yes	yes	yes		
yes	yes	yes	yes	yes	yes	yes		yes
yes	yes	yes	no	yes	yes	yes		yes
yes	yes	yes	yes	yes	yes	yes	no	yes
yes	yes	yes	no	yes	yes	yes		yes
yes	yes	yes	no	yes	yes	yes	no	no

SUM	Respected	AGEOFMA	PARITYCA	TIMELABC	ANCCAT	LENGTHC	EXPERIAN	WORKHRI
13	respected	20-29	2-3	1.1-2	5above	2-3	6-10	8
9	disrespecte	20-29	1	2.1above	3-4	2-3	5	8
11	disrespecte	20-29	1	2.1above	5above	1	5	8
11	disrespecte	20-29	1	2.1above	3-4	4above	5	13above
9	disrespecte	20-29	2-3	1.1-2	3-4	1	5	8
12	disrespecte	20-29	1	2.1above	3-4	1	5	8
11	disrespecte	30above	2-3	2.1above	3-4	1	5	8
12	disrespecte	30above	1	1.1-2	3-4	1	5	8
11	disrespecte	30above	2-3	1	3-4	1	6-10	13above
13	respected	30above	2-3	2.1above	3-4	1	5	8
11	disrespecte	20-29	2-3	2.1above	3-4	2-3	5	8
12	disrespecte	30above	2-3	2.1above	3-4	1	5	13above
12	disrespecte	30above	2-3	2.1above	1-2	1	5	8
11	disrespecte	20-29	1	2.1above	3-4	1	5	8
12	disrespecte	20-29	1	2.1above	5above	1	5	13above
10	disrespecte	20-29	1	2.1above	3-4	1	5	8
13	respected	20-29	2-3	1	3-4	1	5	8
11	disrespecte	30above	2-3	2.1above	3-4	1	5	8
11	disrespecte	20-29	1	1.1-2	3-4	2-3	5	13above
12	disrespecte	20-29	2-3	2.1above	5above	2-3	5	8
12	disrespecte	30above	2-3	2.1above	3-4	1	5	8
13	respected	20-29	1	2.1above	5above	4above	5	13above
13	respected	30above	4above	2.1above	3-4	2-3	5	13above
13	respected	30above	2-3	1	5above	2-3	5	13above
12	disrespecte	20-29	1	2.1above	3-4	1	5	8
10	disrespecte	20-29	1	2.1above	3-4	1	5	8
8	disrespecte	30above	4above	2.1above	1-2	1	5	8
12	disrespecte	30above	2-3	2.1above	1-2	1	6-10	9-12
11	disrespecte	20-29	1	2.1above	3-4	2-3	5	8
12	disrespecte	20-29	2-3	2.1above	3-4	2-3	5	8
10	disrespecte	30above	2-3	2.1above	3-4	1	5	8
11	disrespecte	20-29	1	2.1above	3-4	1	5	8
11	disrespecte	20-29	1	2.1above	3-4	2-3	5	8
10	disrespecte	20-29	1	2.1above	3-4	2-3	5	8
11	disrespecte	20-29	1	2.1above	1-2	2-3	5	8
12	disrespecte	20-29	2-3	2.1above	3-4	2-3	5	13above
11	disrespecte	20-29	1	2.1above	3-4	2-3	5	8
12	disrespecte	30above	2-3	2.1above	3-4	1	5	13above
10	disrespecte	20-29	1	2.1above		1	5	8
12	disrespecte	20-29	2-3	1.1-2	5above	4above	5	13above
7	disrespecte	20-29	1	1	3-4	2-3	5	13above
12	disrespecte	20-29	1	1	3-4	1	5	8
13	respected	20-29	1	2.1above	3-4	1	5	8
12	disrespecte	20-29	1	2.1above	3-4	2-3	5	13above
12	disrespecte	20-29	1	2.1above	3-4	2-3	5	13above
11	disrespecte	20-29	1	1	5above	4above	5	8
7	disrespecte	20-29	1	1	1-2	2-3	5	8
8	disrespecte	20-29	2-3	2.1above	5above	1	6-10	8
13	respected	20-29	1	2.1above	3-4	1	6-10	8
10	disrespecte	20-29	2-3	1	5above	2-3	5	13above
10	disrespecte	20-29	2-3	2.1above	3-4	2-3	5	13above

10	disrespecte	30above	1	2.1above	3-4	1	6-10	13above
12	disrespecte	20-29	2-3	1.1-2	3-4	4above	6-10	13above
13	respected	30above	2-3	1.1-2	3-4	2-3	6-10	13above
11	disrespecte	30above	1	2.1above	3-4	2-3	5	8
10	disrespecte	20-29	1	1.1-2	3-4	1	6-10	8
13	respected	20-29	1	1	5above	1	6-10	13above
13	respected	20-29	2-3	2.1above	3-4	1	5	13above
13	respected	20-29	1	2.1above	3-4	1	5	13above
13	respected	20-29	2-3	2.1above	3-4	1	5	13above
13	respected	20-29	1	2.1above	3-4	1	5	13above
13	respected	20-29	2-3	1.1-2	3-4	1	5	13above
13	respected	20-29	1	2.1above	3-4	1	5	13above
13	respected	20-29	4above	2.1above	3-4	1	6-10	9-12
11	disrespecte	20-29	2-3	2.1above	3-4	1	5	8
12	disrespecte	30above	1	1.1-2	3-4	2-3	5	13above
11	disrespecte	20-29	2-3	2.1above	1-2	2-3	5	13above
6	disrespecte	20-29	1	2.1above	3-4	2-3	5	9-12
11	disrespecte	30above	2-3	2.1above	3-4	2-3	5	8
13	respected	20-29	1	2.1above	3-4	2-3	5	13above
12	disrespecte	20-29	2-3	2.1above	3-4	2-3	5	8
13	respected	20-29	1	2.1above	3-4	1	5	8
13	respected	20-29	2-3	1.1-2	3-4	1	6-10	8
12	disrespecte	30above	2-3	2.1above	3-4	1	5	13above
13	respected	30above	2-3	1	3-4	1	6-10	13above
13	respected	20-29	2-3	1.1-2	3-4	1	5	8
9	disrespecte	20-29	2-3	2.1above	1-2	1	5	8
13	respected	20-29	2-3	1	3-4	1	6-10	9-12
12	disrespecte	20-29	2-3	2.1above	1-2	1	6-10	8
12	disrespecte	30above	2-3	2.1above	3-4	1	5	8
11	disrespecte	20-29	1	1.1-2	3-4	2-3	5	8
13	respected	20-29	1	2.1above	3-4	1	5	8
13	respected	20-29	2-3	1	3-4	4above	6-10	13above
12	disrespecte	20-29	1	1	3-4	1	6-10	8
12	disrespecte	20-29	1	2.1above	3-4	2-3	6-10	8
11	disrespecte	30above	4above	2.1above	3-4	2-3	5	8
13	respected	30above	2-3	1	1-2	2-3	5	8
12	disrespecte	30above	4above	2.1above	3-4	2-3	5	8
11	disrespecte	20-29	1	2.1above	5above	4above	6-10	13above
11	disrespecte	20-29	1	1	3-4	2-3	5	8
11	disrespecte	20-29	2-3	2.1above	3-4	2-3	5	8
8	disrespecte	20-29	1	2.1above	1-2	2-3	5	8
11	disrespecte	20-29	1	2.1above	3-4	2-3	5	8
11	disrespecte	20-29	1	2.1above	3-4	1	5	8
13	respected	20-29	1	1	3-4	1	5	8
13	respected	20-29	2-3	2.1above	3-4	1	6-10	13above
9	disrespecte	30above	1	2.1above	1-2	2-3	5	8
13	respected	20-29	1	2.1above	3-4	2-3	5	13above
13	respected	20-29	2-3	1	3-4	1	5	8
12	disrespecte	20-29	2-3	1	3-4	1	5	8
10	disrespecte	15-19	1	1	3-4	1	5	8
12	disrespecte	30above	4above		3-4	4above	5	13above
10	disrespecte	30above	2-3	1.1-2	3-4	1	5	13above

11	disrespecte	30above	4above	2.1above	3-4	1	5	8
11	disrespecte	30above	2-3	2.1above	3-4	1	5	8
11	disrespecte	30above	2-3	2.1above	3-4	1	5	8
11	disrespecte	20-29	2-3	1.1-2	3-4	1	6-10	8
13	respected	20-29	2-3	2.1above	3-4	2-3	5	8
13	respected	20-29	2-3	2.1above	5above	2-3	5	8
12	disrespecte	15-19	1	2.1above	3-4	2-3	6-10	13above
11	disrespecte	20-29	4above	1.1-2	1-2	4above	5	8
12	disrespecte	20-29	2-3		3-4	2-3	6-10	13above
9	disrespecte	30above	4above	2.1above	3-4	2-3	5	8
13	respected	20-29	2-3	2.1above	3-4	1	5	8
13	respected	20-29	2-3	2.1above	3-4	1	5	8
12	disrespecte	30above	4above		3-4	4above	6-10	13above
13	respected	20-29	2-3	1	1-2	2-3	5	13above
12	disrespecte	20-29	1	2.1above	3-4	2-3	6-10	13above
12	disrespecte	20-29	1		1-2	4above	5	8
8	disrespecte	20-29	2-3		5above	4above	5	13above
11	disrespecte	20-29	2-3	2.1above	3-4	4above	6-10	13above
12	disrespecte	30above	1	2.1above	5above	2-3	5	13above
11	disrespecte	20-29	2-3	2.1above	1-2	4above	5	13above
13	respected	20-29	2-3		3-4	4above	5	13above
13	respected	20-29	4above		1-2	4above	5	13above
11	disrespecte	20-29	1	2.1above		4above	5	8
13	respected	20-29	2-3	1	3-4	1	6-10	8
13	respected	30above	2-3	1	3-4	2-3	6-10	9-12
13	respected	20-29	2-3	1	3-4	1	5	8
13	respected	30above	4above	1	1-2	2-3	6-10	13above
10	disrespecte	20-29	4above	1	3-4	1	6-10	8
10	disrespecte	30above	4above	1		1	5	9-12
11	disrespecte	20-29	1	2.1above	3-4	1	6-10	8
13	respected	30above	1	2.1above	3-4	1	5	8
12	disrespecte	30above	4above	2.1above	3-4	2-3	5	9-12
11	disrespecte	30above	4above	2.1above	3-4	2-3	5	9-12
9	disrespecte	20-29	1	1	3-4	1	5	8
9	disrespecte	30above	2-3		3-4	4above	6-10	13above
12	disrespecte	20-29	1	2.1above	3-4	2-3	5	13above
12	disrespecte	20-29	1			2-3	6-10	13above
12	disrespecte	30above	4above	2.1above	3-4	4above	5	13above
11	disrespecte	30above	2-3	2.1above	3-4	2-3	5	13above
13	respected	20-29	2-3	2.1above	3-4	1	5	8
11	disrespecte	20-29	2-3	2.1above	3-4	2-3	5	13above
13	respected	20-29	1		3-4	4above	5	13above
13	respected	20-29	1	2.1above	1-2	2-3	5	8
13	respected	20-29	1	2.1above	3-4	2-3	5	13above
9	disrespecte	20-29	1	2.1above	3-4	2-3	5	13above
13	respected	30above	4above	2.1above	3-4	2-3	5	13above
10	disrespecte	20-29	1	2.1above	3-4	1	6-10	8
13	respected	20-29	2-3	2.1above	3-4	2-3	5	9-12
13	respected	20-29	2-3	2.1above	3-4	2-3	5	9-12
13	respected	20-29	2-3	2.1above	1-2	2-3	6-10	8
13	respected	30above	2-3	2.1above	3-4	2-3	5	8
12	disrespecte	20-29	1	2.1above	3-4	2-3	5	8

12	disrespecte	20-29	2-3		3-4	4above	5	13above
10	disrespecte	30above	2-3	2.1above	1-2	4above	6-10	13above
8	disrespecte	20-29	1		3-4	2-3	6-10	13above
13	respected	30above	2-3	1.1-2	3-4	2-3	6-10	13above
13	respected	30above	2-3	2.1above	3-4	1	5	13above
9	disrespecte	30above	2-3	1	5above	2-3	5	13above
13	respected	20-29	2-3	2.1above	3-4	2-3	5	13above
13	respected	20-29	2-3	1	3-4	1	5	13above
13	respected	20-29	1	2.1above	1-2	1	6-10	8
12	disrespecte	20-29	2-3	1	3-4	1	6-10	8
13	respected	20-29	2-3	1.1-2	3-4	1	5	9-12
11	disrespecte	20-29	1	1	3-4	1	6-10	8
13	respected	30above	2-3	1	3-4	1	6-10	8
13	respected	30above	4above	2.1above	3-4	1	6-10	8
13	respected	20-29	1	1.1-2	3-4	1	5	8
12	disrespecte	30above	4above		3-4	2-3	5	8
13	respected	30above	2-3	2.1above	3-4	1	5	8
12	disrespecte	20-29	2-3		3-4	4above	5	13above
10	disrespecte	20-29	1	2.1above	3-4	4above	5	13above
11	disrespecte	20-29	2-3		3-4	2-3	5	13above
12	disrespecte	30above	2-3	2.1above	5above	2-3	5	13above
13	respected	20-29	1	2.1above	3-4	2-3	5	13above
13	respected	20-29	4above	2.1above	3-4	2-3	5	9-12
13	respected	20-29	1	2.1above	3-4	4above	5	13above
13	respected	30above	2-3	2.1above	3-4	2-3	5	13above
12	disrespecte	15-19	1		3-4	2-3	5	9-12
13	respected	15-19	1		5above	2-3	5	13above
8	disrespecte	20-29	1	2.1above	5above	4above	5	13above
13	respected	20-29	1		5above	4above	5	13above
10	disrespecte	20-29	2-3	1.1-2	3-4	1	5	8
10	disrespecte	20-29	1	1.1-2	3-4	1	5	8
13	respected	20-29	1	2.1above	3-4	4above	6-10	8
13	respected	20-29	1	1	3-4	1	6-10	8
13	respected	20-29	1	2.1above	3-4	1	6-10	8
13	respected	20-29	1	1.1-2	3-4	2-3	6-10	13above
13	respected	30above	1	1	3-4	1	6-10	13above
13	respected	20-29	1	1	3-4	1	6-10	13above
13	respected	20-29	1	1	3-4	1	6-10	13above
12	disrespecte	15-19	1	1	3-4	1	6-10	13above
12	disrespecte	20-29	1	1.1-2	1-2	1	6-10	13above
13	respected	30above	1	2.1above	3-4	1	6-10	13above
7	disrespecte	30above	2-3	1.1-2	1-2	1	6-10	8
8	disrespecte	30above	2-3	1.1-2	3-4	2-3	5	8
13	respected	20-29	2-3	2.1above	3-4	2-3	5	13above
13	respected	20-29	1	2.1above		1	5	8
13	respected	30above	4above	2.1above	3-4	1	6-10	13above
11	disrespecte	30above	4above	1	3-4	1	5	9-12
10	disrespecte	20-29	1	2.1above	5above	1	6-10	13above
11	disrespecte	20-29	1	1.1-2	3-4	4above	6-10	13above
13	respected	20-29	2-3	2.1above	3-4	4above	6-10	13above
13	respected	30above	1	1.1-2	3-4	4above	5	13above
10	disrespecte	20-29	2-3	1.1-2	3-4	4above	6-10	13above

10	disrespecte	30above	2-3	1.1-2	5above	1	6-10	13above
9	disrespecte	20-29	1	2.1above	5above	1	5	8
11	disrespecte	20-29	1	2.1above	3-4	1	5	8
13	respected	30above	4above	2.1above	3-4	1	5	8
9	disrespecte	15-19	1	2.1above		1	5	8
13	respected	30above	4above	2.1above	5above	1	5	8
13	respected	30above	4above	1	3-4	4above	5	8
13	respected	20-29	1	2.1above	3-4	1	5	8
13	respected	20-29	2-3	2.1above	3-4	1	5	8
13	respected	30above	2-3	2.1above	3-4	1	5	8
13	respected	20-29	2-3	2.1above	3-4	1	5	13above
7	disrespecte	30above	4above	2.1above	3-4	2-3	5	13above
12	disrespecte	20-29	1	1.1-2	3-4	1	5	8
12	disrespecte	20-29	1	2.1above	1-2	1	5	8
10	disrespecte	30above	4above	2.1above	5above	1	5	8
13	respected	15-19	1	2.1above	3-4	1	5	8
12	disrespecte	20-29	2-3	2.1above	3-4	1	6-10	8
13	respected	20-29	2-3	2.1above	3-4	4above	5	13above
13	respected	20-29	4above	2.1above		1	5	8
12	disrespecte	30above	4above	2.1above		2-3	5	8
13	respected	30above	1	1.1-2	3-4	2-3	5	8
12	disrespecte	30above	4above	1		2-3	5	8
12	disrespecte	20-29	2-3	1.1-2	1-2	4above	5	8
12	disrespecte	30above	2-3	2.1above	3-4	4above	5	8
12	disrespecte	20-29	2-3	1	3-4	1	6-10	8
13	respected	20-29	1	2.1above	3-4	1	5	8
11	disrespecte	30above	2-3	2.1above	3-4	4above	5	8
7	disrespecte	30above	2-3	2.1above	3-4	2-3	5	8
13	respected	20-29	1	2.1above	3-4	4above	6-10	9-12
11	disrespecte	20-29	1	2.1above	3-4	4above	5	8
11	disrespecte	30above	4above	2.1above	3-4	2-3	5	8
9	disrespecte	30above	2-3	2.1above		2-3	5	8
10	disrespecte	20-29	2-3	1	3-4	1	5	8
10	disrespecte	20-29	2-3	2.1above	3-4	2-3	6-10	13above
13	respected	20-29	1	1.1-2	3-4	1	5	8
13	respected	20-29	1	2.1above	1-2	2-3	5	8
13	respected	20-29	1	2.1above	3-4	1	5	8
13	respected	20-29	1	2.1above	3-4	2-3	5	8
10	disrespecte	15-19	1	2.1above		2-3	5	13above
13	respected	30above	1	2.1above	3-4	4above	6-10	13above
8	disrespecte	30above	2-3	2.1above	1-2	2-3	5	9-12
9	disrespecte	30above	2-3	2.1above	1-2	4above	5	13above
12	disrespecte	30above	2-3	1.1-2	3-4	1	5	8
7	disrespecte	30above	2-3	2.1above		4above	5	13above
13	respected	20-29	1	1	3-4	1	5	8
13	respected	20-29	1	2.1above	1-2	2-3	5	8
8	disrespecte	20-29	1	2.1above	3-4	4above	5	13above
13	respected	20-29	1	1	3-4	1	5	8
12	disrespecte	30above	4above	2.1above	1-2	1	5	13above
13	respected	30above	2-3	1.1-2	3-4	1	5	8
11	disrespecte	20-29	2-3	1.1-2	3-4	4above	5	13above
12	disrespecte	30above	2-3	2.1above	1-2	2-3	5	13above

8	disrespecte	20-29	1	2.1above	1-2	2-3	5	13above
13	respected	20-29	1	2.1above	3-4	1	5	8
8	disrespecte	20-29	1	2.1above	3-4	2-3	5	8
12	disrespecte	30above	4above	2.1above	3-4	2-3	5	13above
13	respected	30above	4above	2.1above	3-4	4above	5	13above
13	respected	30above	2-3	1	3-4	1	5	8
13	respected	30above	4above	2.1above	3-4	4above	6-10	13above
13	respected	20-29	1	2.1above	1-2	2-3	5	8
11	disrespecte	20-29	1	2.1above		4above	6-10	13above
8	disrespecte	30above	4above	2.1above	1-2	4above	6-10	13above
13	respected	30above	2-3	1.1-2	3-4	2-3	6-10	8
11	disrespecte	30above	2-3	2.1above	5above	2-3	5	8
13	respected	20-29	2-3	2.1above	3-4	4above	6-10	13above
12	disrespecte	20-29	1	2.1above	3-4	2-3	5	8
12	disrespecte	15-19	1	2.1above	3-4	4above	6-10	13above
13	respected	30above	4above	1	3-4	1	5	8
11	disrespecte	30above	4above	2.1above	3-4	4above	6-10	13above
8	disrespecte	20-29	1	2.1above	1-2	4above	6-10	13above
11	disrespecte	20-29	2-3	2.1above	1-2	4above	6-10	9-12
13	respected	20-29	1	1.1-2	5above	2-3	5	8
13	respected	15-19	1	2.1above	1-2	2-3	5	9-12
13	respected	30above	2-3	2.1above	5above	4above	6-10	13above
9	disrespecte	15-19	1	2.1above	3-4	2-3	6-10	8
12	disrespecte	20-29	1	2.1above	1-2	2-3	5	13above
9	disrespecte	30above	2-3		5above	4above	5	13above
13	respected	20-29	1	1.1-2	3-4	1	5	8
13	respected	20-29	2-3	2.1above	3-4	2-3	5	8
12	disrespecte	20-29	2-3	2.1above	1-2	2-3	5	8
13	respected	20-29	1	2.1above	3-4	2-3	6-10	13above
10	disrespecte	30above	2-3	2.1above	3-4	4above	5	13above
13	respected	20-29	1	2.1above	3-4	1	5	8
13	respected	30above	1	2.1above	3-4	1	5	13above
13	respected	30above	2-3	1	3-4	1	5	8
12	disrespecte	30above	4above	2.1above	1-2	4above	5	13above
13	respected	30above	4above	2.1above	3-4	4above	5	13above
13	respected	30above	2-3	2.1above	3-4	1	5	8
10	disrespecte	20-29	1	2.1above	1-2	2-3	5	8
12	disrespecte	30above	2-3	1.1-2	1-2	2-3	5	13above
13	respected	30above	2-3	1	3-4	1	5	8
13	respected	30above	2-3	1.1-2	3-4	1	5	9-12
12	disrespecte	30above	4above	2.1above	3-4	4above	5	9-12
13	respected	20-29	2-3	1.1-2	3-4	1	6-10	13above
5	disrespecte	20-29	1	2.1above	3-4	1	5	13above
13	respected	20-29	2-3	2.1above	1-2	1	5	8
11	disrespecte	20-29	1	1	3-4	1	6-10	8
13	respected	20-29	1	1	3-4	1	5	13above
12	disrespecte	30above	2-3	1	3-4	1	6-10	8
13	respected	30above	2-3	2.1above	3-4	1	5	8
12	disrespecte	20-29	1	1.1-2	3-4	1	5	8
13	respected	20-29	4above	1	3-4	1	5	8
11	disrespecte	30above	4above	1.1-2	3-4	1	5	8
13	respected	20-29	1	1	1-2	1	5	13above

13	respected	20-29	2-3	2.1above	3-4	1	5	13above
13	respected	20-29	2-3	2.1above	3-4	1	5	8
13	respected	30above	2-3	2.1above	1-2	1	5	13above
13	respected	20-29	2-3	2.1above	3-4	1	5	13above
13	respected	30above	2-3	1.1-2	3-4	1	5	8
13	respected	20-29	2-3	2.1above	1-2	1	5	8
13	respected	20-29	1	2.1above	3-4	1	5	13above
13	respected	20-29	1	2.1above	1-2	1	5	8
13	respected	20-29	2-3	2.1above	3-4	1	5	8
13	respected	20-29	2-3	2.1above	1-2	1	6-10	13above
10	disrespecte	20-29	1	1	3-4	1	6-10	8
13	respected	20-29	1	1	3-4	1	6-10	13above
12	disrespecte	20-29	2-3	1.1-2	1-2	1	6-10	13above
13	respected	30above	2-3	2.1above	3-4	1	5	8
13	respected	20-29	1	1.1-2	1-2	1	6-10	8
8	disrespecte	20-29	2-3	1.1-2	3-4	1	5	13above
13	respected	15-19	1	1.1-2	3-4	1	6-10	8
13	respected	20-29	1	1	3-4	1	5	13above
11	disrespecte	20-29	1	1.1-2	1-2	1	5	13above
13	respected	30above	2-3	1.1-2	1-2	1	6-10	8
13	respected	20-29	1	1	3-4	1	5	13above
11	disrespecte	30above	2-3	1	3-4	1	6-10	8
13	respected	20-29	2-3	1	3-4	1	5	13above
13	respected	20-29	1	1	3-4	1	5	13above
13	respected	30above	2-3	2.1above	3-4	1	5	8
12	disrespecte	20-29	2-3	2.1above	3-4	1	5	13above
13	respected	20-29	2-3	1	1-2	2-3	5	13above
13	respected	20-29	2-3	2.1above	1-2	1	6-10	8
13	respected	30above	2-3	2.1above	1-2	1	5	13above
10	disrespecte	20-29	1	1.1-2	5above	1	5	13above
13	respected	15-19	1	1.1-2	3-4	1	5	8
11	disrespecte	20-29	2-3	1	3-4	1	5	8
12	disrespecte	20-29	2-3	1	3-4	1	5	8
11	disrespecte	20-29	1	2.1above	1-2	4above	5	8
11	disrespecte	20-29	1	2.1above	1-2	2-3	5	9-12
13	respected	20-29	1	1.1-2	3-4	1	5	8
13	respected	20-29	1	1.1-2	1-2	1	5	8
13	respected	30above	4above	2.1above	3-4	1	5	13above
13	respected	20-29	2-3	1.1-2	1-2	1	5	8
13	respected	20-29	2-3	2.1above	1-2	1	5	13above
11	disrespecte	20-29	1	1	3-4	1	5	8
11	disrespecte	20-29	2-3	2.1above	1-2	1	5	8
11	disrespecte	30above	2-3	2.1above	1-2	4above	5	9-12
11	disrespecte	30above	2-3	1.1-2	3-4	1	5	8
11	disrespecte	30above	4above	2.1above	1-2	4above	6-10	9-12
11	disrespecte	30above	2-3	2.1above	3-4	4above	5	9-12
9	disrespecte	20-29	2-3	2.1above	1-2	1	5	13above
13	respected	20-29	1	1	3-4	1	5	8
13	respected	20-29	1	1	3-4	1	5	8
12	disrespecte	30above	4above	2.1above	1-2	2-3	5	13above
10	disrespecte	30above	2-3	1	3-4	1	5	13above
12	disrespecte	20-29	2-3	2.1above	1-2	1	5	13above

11	disrespecte	20-29	1	1.1-2	1-2	1	5	13above
13	respected	20-29	1	1.1-2	1-2	1	5	8
11	disrespecte	20-29	1	2.1above	1-2	2-3	5	9-12
10	disrespecte	30above	2-3	2.1above	3-4	2-3	5	9-12
11	disrespecte	20-29	2-3	2.1above	1-2	1	5	8
11	disrespecte	20-29	2-3	2.1above	1-2	2-3	5	8
11	disrespecte	30above	2-3	1	5above	1	5	8
11	disrespecte	20-29	2-3	1.1-2	3-4	1	5	9-12
13	respected	20-29	2-3	1.1-2	5above	1	5	13above
10	disrespecte	20-29	2-3	2.1above	1-2	1	5	8
10	disrespecte	30above	2-3	2.1above	1-2	2-3	5	8
11	disrespecte	20-29	2-3	2.1above	1-2	4above	5	9-12
11	disrespecte	30above	2-3	1	3-4	1	6-10	8
11	disrespecte	20-29	2-3	1	3-4	1	5	8
11	disrespecte	20-29	2-3	2.1above	3-4	2-3	5	13above
13	respected	30above	4above	1.1-2	1-2	1	5	8
13	respected	20-29	2-3	1.1-2	3-4	1	5	8
11	disrespecte	20-29	1	1	3-4	1	5	13above
13	respected	20-29	2-3	2.1above	1-2	1	5	8
13	respected	15-19	1	1.1-2	1-2	1	5	8
13	respected	20-29	2-3	1	3-4	1	5	8
13	respected	20-29	1	1	3-4	1	5	13above
13	respected	20-29	2-3	1.1-2	3-4	1	5	8
12	disrespecte	20-29	1	2.1above	5above	2-3	6-10	8
12	disrespecte	15-19	1	1.1-2	3-4	1	5	8
11	disrespecte	20-29	1	1	3-4	1	5	8

AGEATTC.	ANYCOMT	WEALTHII	AGENEWV	SUMDIGNI	Dignity	SUMCONS	Consent	SUMCONF
31above	yes	3	3	3	1	2	1	2
26-30	yes	2	2	2	0	1	0	1
26-30	no	3	2	3	1	1	0	1
26-30	no	2	2	3	1	1	0	1
26-30	yes	4	2	2	0	1	0	1
26-30	no	3	2	3	1	1	0	2
26-30	no	5	2	3	1	1	0	1
26-30	no	3	2	3	1	2	1	1
31above	no	2	4	3	1	2	1	1
26-30	no	2	2	3	1	2	1	2
26-30	yes	4	2	3	1	2	1	1
26-30	yes	5	2	3	1	2	1	1
26-30	no	4	2	3	1	2	1	1
26-30	no	2	2	3	1	1	0	1
26-30	no	4	2	3	1	2	1	1
26-30	yes	5	2	3	1	1	0	0
26-30	no	1	2	3	1	2	1	2
26-30	no	3	1	2	0	2	1	1
26-30	yes	2	2	3	1	2	1	1
26-30	yes	4	1	3	1	2	1	1
26-30	no	4	2	3	1	2	1	1
26-30	no	1	1	3	1	2	1	2
26-30	yes	3	2	3	1	2	1	2
26-30	no	5	2	3	1	2	1	2
26-30	no	1	1	2	0	2	1	2
26-30	no	1	2	2	0	2	1	0
26-30	yes	5	1	0	0	1	0	2
26-30	yes	2	2	2	0	2	1	2
26-30	no	5	2	3	1	2	1	0
26-30	yes	4	1	3	1	2	1	1
31above	no	2	2	3	1	1	0	0
31above	no	1	2	2	0	2	1	1
26-30	no	2	2	3	1	1	0	1
26-30	no	2	2	2	0	1	0	1
26-30	no	1	2	3	1	1	0	1
26-30	yes	5	2	3	1	2	1	1
26-30	yes	2	2	3	1	1	0	1
26-30	no	2	2	3	1	2	1	1
26-30	no	3	2	3	1	1	0	1
26-30	yes	3	2	3	1	2	1	1
26-30	yes	2	2	3	1	1	0	0
26-30	no	1	1	3	1	2	1	2
26-30	no	1	1	3	1	2	1	2
26-30	no	3	2	2	0	2	1	2
31above	yes	5	3	3	1	2	1	1
31above	yes	1	3	3	1	1	0	1
26-30	yes	5	2	0	0	0	0	1
31above	no	1	5	2	0	1	0	1
26-30	yes	2	2	3	1	2	1	2
26-30	no	4	2	3	1	2	1	1
31above	no	5	4	3	1	0	0	1

31above	yes	4	3	3	1	1	0	0
31above	yes	3	3	3	1	2	1	1
31above	no	4	5	3	1	2	1	2
26-30	yes	2	2	3	1	2	1	1
31above	yes	1	3	3	1	1	0	0
31above	no	4	3	3	1	2	1	2
31above	no	5	4	3	1	2	1	2
31above	yes	5	4	3	1	2	1	2
31above	no	4	4	3	1	2	1	2
25	no	4	1	3	1	2	1	2
25	no	2	1	3	1	2	1	2
31above	yes	2	4	3	1	2	1	2
26-30	no	4	2	3	1	2	1	2
31above	yes	2	3	3	1	1	0	1
26-30	yes	3	2	3	1	2	1	1
31above	yes	4	3	3	1	1	0	1
31above	yes	2	3	1	0	0	0	1
26-30	yes	3	2	3	1	1	0	1
26-30	yes	2	2	3	1	2	1	2
31above	no	2	3	3	1	2	1	1
31above	no	1	4	3	1	2	1	2
31above	no	1	3	3	1	2	1	2
31above	no	4	4	3	1	2	1	1
31above	yes	1	4	3	1	2	1	2
26-30	no	1	2	3	1	2	1	2
26-30	no	1	2	3	1	0	0	2
31above	no	1	3	3	1	2	1	2
31above	yes	1	3	3	1	1	0	2
25	no	5	1	3	1	1	0	2
25	no	2	1	3	1	0	0	2
26-30	no	5	2	3	1	2	1	2
31above	yes	3	4	3	1	2	1	2
26-30	no	2	2	3	1	1	0	2
26-30	no	3	2	3	1	2	1	1
25	no	4	1	3	1	2	1	2
31above	no	1	3	3	1	2	1	2
25	no	3	1	3	1	1	0	2
31above	yes	1	3	3	1	1	0	1
26-30	no	2	1	3	1	0	0	2
26-30	yes	4	1	3	1	1	0	1
26-30	yes	2	2	2	0	1	0	1
31above	no	1	3	3	1	2	1	2
26-30	no	2	1	3	1	2	1	1
26-30	no	1	2	3	1	2	1	2
31above	yes	5	4	3	1	2	1	2
26-30	yes	5	2	2	0	0	0	2
26-30	no	3	2	3	1	2	1	2
26-30	no	4	2	3	1	2	1	2
31above	no	4	3	3	1	2	1	2
26-30	no	1	1	2	0	1	0	1
26-30	yes	1	2	3	1	2	1	1
26-30	no	1	1	3	1	0	0	1

26-30	no	4	2	3	1	1	0	1
31above	no	5	3	3	1	1	0	1
26-30	no	5	2	3	1	1	0	1
31above	no	5	3	3	1	1	0	1
26-30	no	3	2	3	1	2	1	2
26-30	no	1	2	3	1	2	1	2
31above	yes	5	4	3	1	1	0	2
26-30	no	4	2	3	1	0	0	2
31above	no	1	4	3	1	2	1	1
26-30	yes	4	2	2	0	1	0	2
26-30	no	3	2	3	1	2	1	2
26-30	no	5	2	3	1	2	1	2
31above	yes	3	5	3	1	1	0	2
31above	yes	2	3	3	1	2	1	2
31above	yes	3	5	3	1	2	1	1
26-30	yes	1	2	3	1	1	0	2
31above	yes	4	3	2	0	2	1	0
31above	yes	4	5	3	1	1	0	1
26-30	yes	3	2	3	1	1	0	2
31above	yes	4	2	3	1	2	1	0
31above	yes	5	3	3	1	2	1	2
31above	yes	5	3	3	1	2	1	2
26-30	yes	3	2	3	1	0	0	2
31above	no	3	3	3	1	2	1	2
31above	no	2	3	3	1	2	1	2
31above	no	5	3	3	1	2	1	2
31above	no	5	4	3	1	2	1	2
31above	yes	3	3	3	1	1	0	2
31above	no	5	3	3	1	2	1	2
26-30	no	4	2	3	1	1	0	1
26-30	no	5	2	3	1	2	1	2
26-30	yes	3	2	3	1	2	1	1
25	yes	2	1	3	1	1	0	2
26-30	no	3	2	3	1	2	1	1
31above	yes	4	3	3	1	2	1	2
31above	yes	3	3	3	1	2	1	1
31above	yes	1	3	3	1	1	0	2
26-30	yes	5	2	3	1	1	0	2
31above	yes	1	3	2	0	2	1	1
26-30	yes	1	2	3	1	2	1	2
26-30	yes	5	2	3	1	2	1	2
26-30	yes	3	2	3	1	2	1	2
31above	yes	1	3	3	1	2	1	2
26-30	no	5	2	3	1	2	1	2
26-30	yes	2	2	3	1	1	0	0
26-30	no	2	2	3	1	2	1	2
26-30	yes	4	2	1	0	2	1	2
26-30	no	3	2	3	1	2	1	2
31above	no	5	3	3	1	2	1	2
31above	no	3	3	3	1	2	1	2
26-30	no	5	2	3	1	2	1	2
26-30	no	5	2	3	1	2	1	2

26-30	yes	3	2	3	1	2	1	1
31above	yes	4	3	3	1	0	0	2
31above	yes	1	3	1	0	1	0	1
26-30	no	2	2	3	1	2	1	2
26-30	yes	2	1	3	1	2	1	2
26-30	no	5	2	3	1	0	0	2
26-30	no	3	2	3	1	2	1	2
26-30	yes	3	2	3	1	2	1	2
26-30	no	3	2	3	1	2	1	2
26-30	no	1	2	3	1	2	1	1
26-30	no	4	1	3	1	2	1	2
31above	no	1	3	3	1	2	1	2
26-30	no	5	2	3	1	2	1	2
26-30	no	1	2	3	1	2	1	2
25	no	3	1	3	1	2	1	2
26-30	no	3	2	3	1	2	1	1
26-30	no	2	2	3	1	2	1	2
26-30	yes	3	2	3	1	2	1	1
26-30	yes	4	2	3	1	1	0	0
26-30	no	5	2	2	0	2	1	1
31above	yes	4	3	3	1	1	0	2
26-30	yes	1	2	3	1	2	1	2
26-30	no	5	2	3	1	2	1	2
31above	yes	3	3	3	1	2	1	2
31above	no	4	3	3	1	2	1	2
31above	yes	5	3	3	1	1	0	2
26-30	yes	5	2	3	1	2	1	2
31above	yes	5	2	2	0	0	0	2
26-30	yes	1	2	3	1	2	1	2
26-30	no	4	2	3	1	2	1	0
26-30	no	2	1	3	1	2	1	0
26-30	yes	2	2	3	1	2	1	2
26-30	no	1	2	3	1	2	1	2
26-30	no	5	2	3	1	2	1	2
31above	yes	5	4	3	1	2	1	2
26-30	no	4	2	3	1	2	1	2
26-30	no	2	2	3	1	2	1	2
31above	no	2	3	3	1	2	1	2
31above	no	4	3	3	1	2	1	1
26-30	yes	1	2	3	1	1	0	2
31above	no	3	3	3	1	2	1	2
26-30	no	5	2	1	0	0	0	2
26-30	yes	5	2	2	0	0	0	2
26-30	no	1	2	3	1	2	1	2
26-30	no	3	1	3	1	2	1	2
26-30	no	4	2	3	1	2	1	2
26-30	yes	3	1	3	1	2	1	1
26-30	no	2	1	3	1	1	0	1
31above	no	3	3	3	1	2	1	1
31above	no	1	3	3	1	2	1	2
31above	no	2	3	3	1	2	1	2
31above	no	5	3	3	1	2	1	1

26-30	no	2	1	3	1	1	0	1
26-30	no	4	2	3	1	1	0	2
25	no	4	1	3	1	1	0	2
25	no	3	1	3	1	2	1	2
25	no	5	1	2	0	2	1	1
26-30	no	2	2	3	1	2	1	2
26-30	no	4	2	3	1	2	1	2
26-30	no	5	1	3	1	2	1	2
26-30	no	4	1	3	1	2	1	2
26-30	no	5	1	3	1	2	1	2
26-30	no	3	2	3	1	2	1	2
26-30	yes	1	2	2	0	1	0	1
26-30	no	4	2	3	1	2	1	1
26-30	no	2	2	2	0	2	1	2
26-30	no	3	1	3	1	0	0	1
26-30	no	5	2	3	1	2	1	2
31above	yes	1	3	3	1	2	1	1
26-30	no	5	2	3	1	2	1	2
26-30	no	5	2	3	1	2	1	2
26-30	no	5	1	3	1	2	1	2
26-30	yes	3	2	3	1	2	1	2
26-30	no	5	1	3	1	2	1	2
26-30	no	5	1	3	1	1	0	2
26-30	no	2	2	3	1	1	0	2
31above	no	1	3	3	1	1	0	2
26-30	yes	5	2	3	1	2	1	2
26-30	yes	5	2	2	0	1	0	2
26-30	no	5	2	2	0	0	0	0
31above	yes	1	3	3	1	2	1	2
26-30	yes	1	1	3	1	1	0	2
26-30	no	3	1	3	1	0	0	2
26-30	no	1	2	2	0	1	0	2
26-30	no	4	1	3	1	1	0	0
31above	yes	1	3	2	0	2	1	2
25	no	4	1	3	1	2	1	2
25	no	2	1	3	1	2	1	2
25	yes	1	1	3	1	2	1	2
25	no	2	1	3	1	2	1	2
25	yes	1	1	2	0	2	1	2
31above	yes	1	2	3	1	2	1	2
26-30	yes	1	2	2	0	0	0	2
26-30	yes	4	2	3	1	1	0	1
26-30	no	3	2	2	0	2	1	2
26-30	yes	1	2	1	0	0	0	2
26-30	no	4	2	3	1	2	1	2
26-30	no	1	2	3	1	2	1	2
26-30	yes	4	2	2	0	0	0	2
26-30	no	4	2	3	1	2	1	2
26-30	no	4	2	3	1	2	1	1
25	no	4	1	3	1	2	1	2
31above	yes	3	3	3	1	0	0	2
31above	yes	3	3	3	1	2	1	1

26-30	yes	1	2	2	0	1	0	1
25	no	3	1	3	1	2	1	2
26-30	no	5	2	2	0	0	0	2
31above	no	4	3	3	1	2	1	1
31above	yes	3	3	3	1	2	1	2
26-30	no	1	2	3	1	2	1	2
31above	yes	4	4	3	1	2	1	2
26-30	yes	5	2	3	1	2	1	2
31above	yes	3	3	2	0	1	0	2
31above	yes	1	3	2	0	0	0	2
31above	yes	5	3	3	1	2	1	2
25	yes	5	1	3	1	1	0	1
31above	yes	3	3	3	1	2	1	2
26-30	no	5	2	3	1	2	1	1
31above	yes	1	3	3	1	2	1	1
26-30	no	2	2	3	1	2	1	2
31above	yes	4	3	3	1	1	0	1
31above	yes	1	3	1	0	1	0	2
31above	yes	4	3	3	1	2	1	1
26-30	no	1	2	3	1	2	1	2
26-30	yes	4	2	3	1	2	1	2
31above	yes	2	3	3	1	2	1	2
26-30	yes	3	2	2	0	0	0	2
26-30	yes	2	2	3	1	2	1	1
26-30	no	2	2	2	0	2	1	1
26-30	no	1	2	3	1	2	1	2
26-30	yes	4	2	3	1	2	1	2
26-30	no	2	2	3	1	2	1	1
26-30	yes	1	2	3	1	2	1	2
26-30	yes	2	2	3	1	0	0	1
26-30	no	4	2	3	1	2	1	2
26-30	no	3	2	3	1	2	1	2
26-30	no	4	2	3	1	2	1	2
26-30	yes	2	2	3	1	2	1	1
26-30	yes	4	2	3	1	2	1	2
26-30	no	5	1	3	1	2	1	2
26-30	yes	2	1	3	1	0	0	1
26-30	yes	3	2	3	1	2	1	1
26-30	no	4	1	3	1	2	1	2
26-30	no	3	2	3	1	2	1	2
26-30	yes	3	2	3	1	1	0	2
26-30	no	3	2	3	1	2	1	2
26-30	no	1	2	0	0	0	0	1
26-30	no	1	2	3	1	2	1	2
26-30	no	3	2	3	1	0	0	2
26-30	no	2	2	3	1	2	1	2
26-30	no	5	2	3	1	1	0	2
25	no	5	1	3	1	2	1	2
25	no	2	1	3	1	1	0	2
25	no	2	1	3	1	2	1	2
25	no	3	1	3	1	1	0	2
25	no	1	1	3	1	2	1	2

26-30	no	2	2	3	1	2	1	2
26-30	no	3	2	3	1	2	1	2
26-30	no	3	2	3	1	2	1	2
26-30	no	2	2	3	1	2	1	2
26-30	no	3	2	3	1	2	1	2
26-30	yes	2	2	3	1	2	1	2
26-30	no	2	2	3	1	2	1	2
26-30	no	2	2	3	1	2	1	2
26-30	no	2	2	3	1	2	1	2
26-30	yes	2	2	3	1	2	1	2
26-30	no	1	2	3	1	1	0	2
26-30	no	1	2	3	1	2	1	2
26-30	no	3	2	3	1	2	1	2
26-30	no	5	1	3	1	2	1	2
26-30	no	2	2	3	1	2	1	2
31above	no	2	2	1	0	0	0	2
26-30	no	4	2	3	1	2	1	2
26-30	no	4	2	3	1	2	1	2
26-30	no	5	2	3	1	0	0	2
26-30	no	4	2	3	1	2	1	2
26-30	no	1	1	3	1	2	1	2
26-30	yes	1	2	3	1	2	1	2
26-30	no	5	1	3	1	2	1	2
25	no	4	1	3	1	2	1	2
25	no	2	1	3	1	2	1	2
25	no	4	1	3	1	1	0	2
26-30	no	2	1	3	1	2	1	2
31above	no	2	3	3	1	2	1	2
26-30	no	3	2	3	1	2	1	2
26-30	no	2	2	2	0	0	0	2
26-30	no	5	2	3	1	2	1	2
26-30	yes	4	2	3	1	1	0	1
26-30	no	4	2	3	1	2	1	2
26-30	yes	4	2	3	1	1	0	1
26-30	yes	2	2	3	1	1	0	1
26-30	no	4	2	3	1	2	1	2
31above	no	1	2	3	1	2	1	2
26-30	no	4	2	3	1	2	1	2
26-30	no	2	2	3	1	2	1	2
26-30	no	4	2	3	1	2	1	2
26-30	yes	3	2	3	1	1	0	1
26-30	yes	3	2	3	1	1	0	1
26-30	yes	5	2	3	1	1	0	1
26-30	yes	4	2	3	1	1	0	1
31above	yes	5	2	3	1	1	0	1
26-30	yes	2	2	3	1	1	0	1
26-30	yes	1	2	3	1	1	0	1
26-30	no	4	2	3	1	2	1	2
26-30	no	1	2	3	1	2	1	2
26-30	yes	3	2	3	1	2	1	1
26-30	no	2	2	3	1	0	0	1
31above	yes	3	3	3	1	2	1	1

31above	yes	2	3	2	0	2	1	1
26-30	yes	1	1	3	1	2	1	2
26-30	yes	3	2	3	1	1	0	1
26-30	yes	3	2	3	1	0	0	1
26-30	yes	5	2	3	1	1	0	1
26-30	yes	1	2	3	1	1	0	1
26-30	no	3	2	3	1	1	0	1
26-30	yes	5	2	3	1	1	0	1
26-30	no	3	2	3	1	2	1	2
26-30	yes	3	2	3	1	0	0	1
26-30	yes	5	2	3	1	0	0	1
31above	yes	5	3	3	1	1	0	1
26-30	no	3	2	3	1	1	0	1
26-30	no	2	2	3	1	1	0	1
26-30	no	1	2	3	1	1	0	1
26-30	no	5	2	3	1	2	1	2
26-30	no	3	2	3	1	2	1	2
26-30	no	1	2	3	1	1	0	2
26-30	no	4	1	3	1	2	1	2
26-30	no	1	1	3	1	2	1	2
26-30	no	4	1	3	1	2	1	2
26-30	no	3	1	3	1	2	1	2
31above	no	3	2	3	1	2	1	2
31above	yes	5	3	3	1	1	0	2
26-30	no	5	2	3	1	1	0	2
26-30	no	2	2	3	1	1	0	1

Confidentia	SUMNOND	NONDISCI	SUMNONA	NONABAN
1	2	1	2	1
0	1	0	2	1
0	2	1	2	1
0	2	1	2	1
0	2	1	1	0
1	2	1	2	1
0	2	1	2	1
0	2	1	2	1
0	2	1	1	0
1	2	1	2	1
0	1	0	2	1
0	2	1	2	1
0	2	1	2	1
0	2	1	2	1
0	2	1	2	1
0	2	1	2	1
1	2	1	2	1
0	2	1	2	1
0	2	1	1	0
0	2	1	2	1
0	2	1	2	1
1	2	1	2	1
1	2	1	2	1
1	2	1	2	1
1	2	1	2	1
0	2	1	2	1
1	2	1	1	0
1	2	1	2	1
0	2	1	2	1
0	2	1	2	1
0	2	1	2	1
0	2	1	2	1
0	2	1	2	1
0	2	1	2	1
0	2	1	2	1
0	2	1	2	1
0	2	1	2	1
0	2	1	2	1
0	1	0	2	1
0	2	1	2	1
0	1	0	0	0
1	1	0	2	1
1	2	1	2	1
1	2	1	2	1
0	2	1	2	1
0	2	1	2	1
0	2	1	2	1
0	2	1	0	0
1	2	1	2	1
0	1	0	1	0
0	2	1	2	1

0	2	1	2	1
0	2	1	2	1
1	2	1	2	1
0	1	0	2	1
0	2	1	2	1
1	2	1	2	1
1	2	1	2	1
1	2	1	2	1
1	2	1	2	1
1	2	1	2	1
1	2	1	2	1
1	2	1	2	1
1	2	1	2	1
0	2	1	2	1
0	2	1	2	1
0	2	1	2	1
0	2	1	0	0
0	2	1	2	1
1	2	1	2	1
0	2	1	2	1
1	2	1	2	1
1	2	1	2	1
0	2	1	2	1
1	2	1	2	1
1	2	1	2	1
1	1	0	1	0
1	2	1	2	1
1	2	1	2	1
1	2	1	2	1
1	2	1	2	1
1	2	1	2	1
1	2	1	2	1
1	2	1	2	1
0	2	1	2	1
1	2	1	0	0
1	2	1	2	1
1	2	1	2	1
0	2	1	2	1
1	2	1	2	1
0	2	1	2	1
0	2	1	2	1
1	2	1	2	1
0	2	1	2	1
1	2	1	2	1
1	2	1	2	1
1	2	1	2	1
1	2	1	2	1
1	2	1	2	1
1	1	0	2	1
0	2	1	2	1
0	2	1	2	1
0	2	1	2	1

0	2	1	2	1
0	2	1	2	1
0	2	1	2	1
0	2	1	2	1
1	2	1	2	1
1	2	1	2	1
1	2	1	2	1
1	2	1	2	1
0	2	1	2	1
1	2	1	2	1
1	2	1	2	1
1	2	1	2	1
1	2	1	2	1
1	2	1	2	1
0	2	1	2	1
1	2	1	2	1
0	2	1	0	0
0	2	1	2	1
1	2	1	2	1
0	2	1	2	1
1	2	1	2	1
1	2	1	2	1
1	2	1	2	1
1	2	1	2	1
1	2	1	2	1
1	2	1	2	1
1	2	1	2	1
1	2	1	0	0
1	2	1	0	0
0	2	1	2	1
1	2	1	2	1
0	2	1	2	1
1	2	1	2	1
0	1	0	0	0
1	2	1	0	0
0	2	1	2	1
1	2	1	2	1
1	2	1	2	1
0	2	1	2	1
1	2	1	2	1
1	2	1	0	0
1	2	1	2	1
1	2	1	2	1
1	2	1	2	1
0	1	0	2	1
1	2	1	2	1
1	2	1	2	1
1	2	1	2	1
1	2	1	2	1
1	2	1	2	1
1	2	1	2	1
1	2	1	2	1

0	2	1	2	1
1	2	1	1	0
0	2	1	2	1
1	2	1	2	1
1	2	1	2	1
1	2	1	0	0
1	2	1	2	1
1	2	1	2	1
1	2	1	2	1
0	2	1	2	1
1	2	1	2	1
1	2	1	0	0
1	2	1	2	1
1	2	1	2	1
1	2	1	2	1
0	2	1	2	1
1	2	1	2	1
0	2	1	2	1
0	2	1	2	1
0	2	1	2	1
1	2	1	2	1
1	2	1	2	1
1	2	1	2	1
1	2	1	2	1
1	2	1	2	1
1	2	1	2	1
1	2	1	2	1
1	2	1	0	0
1	2	1	2	1
0	1	0	2	1
0	1	0	2	1
1	2	1	2	1
1	2	1	2	1
1	2	1	2	1
1	2	1	2	1
1	2	1	2	1
1	2	1	2	1
1	2	1	2	1
0	2	1	2	1
1	2	1	2	1
1	2	1	2	1
1	2	1	0	0
1	2	1	0	0
1	2	1	2	1
1	2	1	2	1
1	2	1	2	1
0	1	0	2	1
0	1	0	2	1
0	1	0	2	1
1	2	1	2	1
1	2	1	2	1
0	1	0	1	0

0	1	0	2	1
1	1	0	0	0
1	1	0	2	1
1	2	1	2	1
0	1	0	1	0
1	2	1	2	1
1	2	1	2	1
1	2	1	2	1
1	2	1	2	1
1	2	1	2	1
1	2	1	2	1
0	1	0	0	0
0	2	1	2	1
1	2	1	2	1
0	2	1	2	1
1	2	1	2	1
0	2	1	2	1
1	2	1	2	1
1	2	1	2	1
1	2	1	2	1
1	2	1	2	1
1	2	1	2	1
1	2	1	2	1
1	2	1	2	1
1	2	1	2	1
1	2	1	2	1
1	2	1	2	1
0	1	0	2	1
1	2	1	2	1
1	1	0	2	1
1	2	1	2	1
1	1	0	1	0
0	2	1	2	1
1	2	1	0	0
1	2	1	2	1
1	2	1	2	1
1	2	1	2	1
1	2	1	2	1
1	2	1	0	0
1	2	1	2	1
1	2	1	0	0
0	2	1	0	0
1	2	1	2	1
1	2	1	0	0
1	2	1	2	1
1	2	1	2	1
1	2	1	0	0
1	2	1	2	1
0	2	1	2	1
1	2	1	2	1
1	2	1	2	1
0	2	1	2	1

0	2	1	0	0
1	2	1	2	1
1	2	1	0	0
0	2	1	2	1
1	2	1	2	1
1	2	1	2	1
1	2	1	2	1
1	2	1	2	1
1	2	1	2	1
1	2	1	0	0
1	2	1	2	1
0	2	1	2	1
1	2	1	2	1
0	2	1	2	1
0	2	1	2	1
1	2	1	2	1
0	2	1	2	1
1	2	1	0	0
0	1	0	2	1
1	2	1	2	1
1	2	1	2	1
1	2	1	2	1
1	2	1	1	0
0	2	1	2	1
0	2	1	0	0
1	2	1	2	1
1	2	1	2	1
0	2	1	2	1
1	2	1	2	1
0	2	1	2	1
1	2	1	2	1
1	2	1	2	1
1	2	1	2	1
0	2	1	2	1
1	2	1	2	1
1	2	1	2	1
0	2	1	2	1
0	2	1	2	1
1	2	1	2	1
1	2	1	2	1
1	2	1	2	1
1	2	1	2	1
0	2	1	0	0
1	2	1	2	1
1	2	1	2	1
1	2	1	2	1
1	2	1	2	1
1	2	1	2	1
1	2	1	2	1
1	2	1	2	1
1	1	0	2	1
1	2	1	2	1

1	2	1	2	1
1	2	1	2	1
1	2	1	2	1
1	2	1	2	1
1	2	1	2	1
1	2	1	2	1
1	2	1	2	1
1	2	1	2	1
1	2	1	2	1
1	2	1	2	1
1	2	1	0	0
1	2	1	2	1
1	2	1	1	0
1	2	1	2	1
1	2	1	2	1
1	2	1	1	0
1	2	1	2	1
1	2	1	2	1
1	2	1	2	1
1	2	1	2	1
1	2	1	2	1
1	2	1	0	0
1	2	1	2	1
1	2	1	2	1
1	2	1	2	1
1	2	1	2	1
1	2	1	2	1
1	2	1	2	1
1	2	1	2	1
1	2	1	2	1
1	2	1	2	1
0	2	1	2	1
1	1	0	2	1
0	2	1	2	1
0	2	1	2	1
1	2	1	2	1
1	2	1	2	1
1	2	1	2	1
1	2	1	2	1
1	2	1	2	1
0	2	1	2	1
0	2	1	2	1
0	2	1	2	1
0	2	1	2	1
0	2	1	2	1
0	2	1	2	1
0	2	1	0	0
1	2	1	2	1
1	2	1	2	1
0	2	1	2	1
0	2	1	2	1
0	2	1	2	1

0	2	1	2	1
1	2	1	2	1
0	2	1	2	1
0	2	1	2	1
0	2	1	2	1
0	2	1	2	1
0	2	1	2	1
0	2	1	2	1
1	2	1	2	1
0	2	1	2	1
0	2	1	2	1
0	2	1	2	1
0	2	1	2	1
0	2	1	2	1
0	2	1	2	1
1	2	1	2	1
1	2	1	2	1
1	2	1	1	0
1	2	1	2	1
1	2	1	2	1
1	2	1	2	1
1	2	1	2	1
1	2	1	2	1
1	2	1	2	1
1	2	1	2	1
0	2	1	2	1
