# Supplementary material for: Respectful delivery care and associated factors among mothers delivered in public health facilities of Dessie city, Northeast Ethiopia: a cross-sectional study
Source: BMC Womens Health. 2022 Apr 21;22:127. doi: 10.1186/s12905-022-01713-1 (PMC9026676; doi:10.1186/s12905-022-01713-1)
Supplement: Supplementary file 2 — Additional file 2: The English version questionaries. [file 12905_2022_1713_MOESM2_ESM.pdf]

Annexes

Annex I: Information sheet and consent form in English

1. **Title of the research;** respectful delivery care and its associated factors among mothers delivered in health facilities of Dessie city, Ethiopia, 2018.

Name of Principal Investigator: Melaku Yalew

Name of Co-Investigator Dabere Nigatu, Toyeb Yasin, Bereket Kefale and Yitayish Damtie

Name of the organization: Wollo University

Introduction: This information sheet and consent form is prepared by research investigators from Wollo and Bahir Dar University, School of Public health. The aim of this study is to assess respectful delivery care and its associated factors among mothers delivered in health facilities of Dessie city, Ethiopia.

Risk and Confidentiality: There is no any risk or discomfort that you will face by participating in this research except dedication of 30 minute time for responding to the written questionnaires. Your name is not written on the questionnaire and all what you say have no any means to transferred to any other individual or institution. The information collected from this research project was kept safely and confidentially and it will be accessed by the researcher and research assistant only.

Incentives and Rights for Participating; Even though, there is no incentive or payment that you will obtain by participating in this project, you may get indirect benefit from the findings of the study that is help full with no doubt to improve quality of care in delivery services. You were not facing any problem if you are not voluntary and even, you have also the right to with draw from the study at any time without any need to explain the reason to anyone.

2. **Informed Consent**

Dear respondent! Good morning/good afternoon my name is -------------------------------- and I am collecting the data on respectful deliver care and its associated factors among mothers delivered in Health facilities of Dessie city. You are selected randomly and your name was not being written in this form. Whatever information you provide will be kept strictly confidential, and will not be shared with anyone else. I am going to ask you some questions that are not

difficult to answer which lasts about 30 minute and you have a right to participate or not, or discontinue at any time. However, your honest answers to these questions will be very helpful for the success of the research.

Would you be willing to participate [indicate by ticking the appropriate responses]?

Yes, I want to participate in the study (please go to the next page) ☐

No, I don't want to participate. ☐ (Stop) Thank you very much!

Name of the Interviewer ______________ Signature_________ date _________

For more information use the following address and please contact him;(Melaku Yalew= **0906122424**)

Annex III: Questionnaire in English

Patient ID number/ Code number _______________ woreda _______________ kebele ___________

Date of interview……………… Time started……………: ……… Time finished………. ………:

Part I: Socio-demographic data

No	Question	Option/Code	Skip
101	How old are you in completed years? ------------------------		
102	What is your current place of residence	Rural-------1<br>Urban-------2	
103	What is your current marital status	Single-------1<br>Currently married-------2<br>divorced-------3<br>widowed-------4	
104	What is the highest level of education you have completed?	Unable to read and write-----1<br>Only read and write------ 2<br>Primary school (1-8)------- 3<br>Secondary school (9-12)---- 4<br>College and above--------- 5	
105	What is your religion?	Orthodox-------1	

		Muslim-------2<br>Protestant-------3<br>Others specify-------4	
106	What is your ethnicity?	Amhara-------1Oromo-------2Tigray-------3Others specify-------4	
107	What is your current occupation?	Government employee------- 1<br>merchant………………....2<br>Private employee------- 3<br>Student------- 4<br>House wife-------5<br>Others specify-------6	
**Urban wealth index**			

	What is the source of your drinking water? (more than one answer is possible)			
108	1.houseline water	Yes ... 1	No ... 0	
109	2. pull and push/sway common water	Yes ... 1	No ... 0	
110	3. bono water	Yes ... 1	No ... 0	
111	4.protective pond water	Yes ... 1	No ... 0	
112	5.un protective pond water	Yes ... 1	No ... 0	
113	6.stream water	Yes ... 1	No ... 0	
114	7. if others list……..			
115	What type of toilet do you use?			
	1.Water flush	Yes ... 1	No ... 0	
	2. Traditional toilet	Yes ... 1	No ... 0	
	3. Ventilated improved pit latrine	Yes ... 1	No ... 0	
	4. Open field	Yes ... 1	No ... 0	
	5. Others (list)…….			

116	Who is the owner of your living house? 1. My own 0. rent house	
117	Does your living house have dividing class? Yes ... 1 No ... 0	
118	Do you have separated bedroom? Yes ... 1 No ... 0	
119	Do you have separated kitchen? Yes ... 1 No ... 0	
	From which material your house floor is made? (more than one answer is possible)	
120	1. Natural ground Yes ... 1 No ... 0	
121	2. Muck/smooth by cows faces Yes ... 1 No ... 0	
122	3. Wood Yes ... 1 No ... 0	
123	4. Cement Yes ... 1 No ... 0	
124	5. if others list ……..	
125	From which material your house roof is made?<br>0. Grass/ leaf 1. corrugated iron	
	From which material your house wall is made? (more than one answer is possible)	
126	1. Wood but not have mod Yes ... 1 No ... 0	
127	2. Wood with mod Yes ... 1 No ... 0	
128	3. Wood and cement Yes ... 1 No ... 0	
129	4. Blocket Yes ... 1 No ... 0	
130	5. if others list….	
	What is your energy source for food cooking? (more than one answer is possible)	
131	1. Electric city system Yes ... 1 No ... 0	
132	2. Gas /kerosene Yes ... 1 No ... 0	
133	3. Wood /leaf Yes ... 1 No ... 0	
134	4. Charcoal Yes ... 1 No ... 0	
135	5. Animal faeces Yes ... 1 No ... 0	
136	6. If other list…	

	Among the following materials, which one do you own? (more than one answer is possible)	
137	1. Radio Yes ... 1 No ... 0	
138	2. Television Yes ... 1 No ... 0	
139	3. House phone Yes ... 1 No ... 0	
140	4. Fridge Yes ... 1 No ... 0	
141	5. Chair Yes ... 1 No ... 0	
142	6. Table Yes ... 1 No ... 0	
143	7. Bed and mattress which made from cotton spring Yes ... 1 No ... 0	
144	8. Mobile Yes ... 1 No ... 0	
145	9. Cycle Yes ... 1 No ... 0	
146	10. Motor cycle Yes ... 1 No ... 0	
147	11. Horse’s cart Yes ... 1 No ... 0	
148	12. Bajaj/car Yes ... 1 No ... 0	
149	13.Bank book Yes ... 1 No ... 0	
150	14. If other list ….	
	Rural wealth index	
151	Watch Yes ... 1 No ... 0	
152	Sofa Yes ... 1 No ... 0	
153	Chair Yes ... 1 No ... 0	
154	Table Yes ... 1 No ... 0	
155	Bed and mattress which made from cotton spring Yes ... 1 No ... 0	
156	Horse’s Cart Yes ... 1 No ... 0	
157	If others specify...	
158	Do you have your own farm for the purpose of agriculture/cropping? Yes ... 1 No ... 0	

	From the following household animal do you have? (more than one answer is possible)	
159	1. Ox/ cow Yes ...1 No ... 0	
160	2. Horse/donkey/ mule Yes ... 1 No ... 0	
161	3. Goat Yes ... 1 No ... 0	
162	4. Sheep Yes ... 1 No ... 0	
163	5. Hen Yes ... 1 No ... 0	
164	6. Beehive Yes ...1 No ... 0	
165	7. Others..................	

Part II: Client related variable

No	Question	Option	skip
201	Did you have companion/supporter while you was in labour?	Yes-------1<br>No-------0	
202	Did the provider have any attempt for one or more of the following during your ANC?<br>1.pinch Yes…..1 No…..0<br>2.Kick Yes…..1 No…..0<br>3.Rape Yes…..1 No…..0<br>4. If other specify……..		
203	Did you have previously delivered at this facility?	Yes-------1<br>No-------0	
204	Did you have previously using the facility other than delivery?	Yes-------1<br>No-------0	
205	Are you referred from another health facility?	Yes-------1<br>No-------0	
206	Have you been screened for HIV during antenatal care or delivery?	Yes-------1	If no skip to

		No-------0	QNO 301
207	If yes what was your test result? See her delivery cared?	Reactive------1<br>Non-reactive---2<br>Invalid test-----3	

Part **III: Obstetric related data**

No	Question	Option/ Code	Skip
301	How many total births did you give so far (including still birth and current pregnancy?-------------in number		
302	Did you have antenatal care visit for the current pregnancy?	Yes-------1<br>No-------0	If no skip to QNO 304
303	How many visit did you have?---------------------in number		
304	At what time you arrived in health facility after the labour started?---------in hour		
305	What was her stage of labour while she arrived in health facility? See her delivery card	First------1<br>Second-----2	
306	Have you faced any complications during labour/post-partum?	Yes-------1<br>No-------0	If no skip to QNO 307
307	If yes what was the complication that you face? See her document	Post-partum hemorrhage------- 1<br>Ante-partum hemorrhage------- 2<br>Preeclampsia/Eclampsia------- 3<br>3rd/4th tear------- 4<br>Retained placenta ------- 5<br>Others specify-------6	
308	Has your neonate faced any complications?	Yes-------1No-------0	If no skip to

			QNO 310
309	If yes what was the complication that your neonate face?	Distress-------1<br>Mal-presentation------- 2<br>Physical injury------- 3<br>Others specify-------4	
310	What was the outcome of your current pregnancy?	Alive -------1<br>Dead -------2	
311	What was the time you give birth?	Day-------1<br>Night-------2	
312	What was your mode of delivery? (see the document)	Vaginal-------1<br>Episiotomy--------2<br>instrumental-------3<br>Cesarean section----4<br>Others specify-------5	
313	For how many days did you stay in this facility?-------------------in days		

Part **IV: Health facility and providers related data**

No	Question	Option/ Code	Skip
401	What type of health facility did you give birth?	Health center-------1<br>Hospital-------2	
402	What was the sex of professionals that assist you?(see the document)	Male-------1<br>Female-------2	
403	Profession of her birth attendant?(see the document)	Nurse------- 1<br>Midwifery------- 2<br>Health officer----3<br>Doctor------- 4	

		Others specify-------5	
404	Her birth attendant service experience in the current position?------------in completed years		
405	Her birth attendant estimated work hour per day?------------in hours		

Part V: procedures/ Actions done for the women

No	Procedure/Action done	Option	not applicable
Dignified care			
501	Did the provider actively listen you?	Yes……1<br>No……..0	
502	Did the provider insult/threat/ you?	Yes……1<br>No……..0	
503	Did the provider respond promptly, politely professionally when you ask for help?	Yes……1<br>No……..0	
Consented care			
504	Did the provider obtain consent from you before vaginal examination/procedure?	Yes……1<br>No……..0	
505	Did the provider give information regarding status and progress of labour?	Yes……1<br>No……..0	
Confidential care			
506	Did the provider use visual barriers to protect you while you were in labour?	Yes……1<br>No……..0	
507	Did the provider ensure confidentiality of your information?	Yes……1<br>No……..0	

Non discriminated care			
508	Did the provider discriminate you based on your religion, ethnicity, age, language, HIV/AIDS status?	Yes……1<br>No……..0	
509	Did the provider speak to you in a language that you can understand?	Yes……1<br>No……..0	
Non abandonment care			
510	Did the provider encourage you to call if needed?	Yes……1<br>No……0	
511	Did the provider come quickly when you called him/her?	Yes ……1<br>No……..0	
Non detention care			
512	Did the provider detain you because of payment?	Yes……1<br>No……..0	
Non abused care			
513	Did the provider have any attempt for one or more of the following during and after delivery?<br>1.pinch Yes…..1 No…..0<br>2.Kick Yes…..1 No…..0<br>3.Rape Yes…..1 No…..0<br>4. If other specify……..		
